# SMOTIF: efficient structured pattern and profile motif search

**DOI:** 10.1186/1748-7188-1-22

**Published:** 2006-11-21

**Authors:** Yongqiang Zhang, Mohammed J Zaki

**Affiliations:** 1 Department of Computer Science, Rensselaer Polytechnic Institute, Troy, New York 12180, USA

## Abstract

**Background:**

A structured motif allows variable length gaps between several components, where each component is a simple motif, which allows either no gaps or only fixed length gaps. The motif can either be represented as a *pattern *or a *profile *(also called positional weight matrix). We propose an efficient algorithm, called SMOTIF, to solve the structured motif search problem, i.e., given one or more sequences and a structured motif, SMOTIF searches the sequences for all occurrences of the motif. Potential applications include searching for *long terminal repeat (LTR) retrotransposons *and composite regulatory binding sites in DNA sequences.

**Results:**

SMOTIF can search for both pattern and profile motifs, and it is efficient in terms of both time and space; it outperforms SMARTFINDER, a state-of-the-art algorithm for structured motif search. Experimental results show that SMOTIF is about 7 times faster and consumes 100 times less memory than SMARTFINDER. It can effectively search for LTR retrotransposons and is well suited to searching for motifs with long range gaps. It is also successful in finding potential composite transcription factor binding sites.

**Conclusion:**

SMOTIF is a useful and efficient tool in searching for structured pattern and profile motifs. The algorithm is available as open-source at: .

## Background

Searching biological sequence(s) for motifs is a fundamental task in bioinformatics. Motifs can be represented as either patterns over a specific alphabet, or *profiles *(also called *positional weight matrix *(PWM)), which give the probability of observing each symbol in each position. Motifs can be classified into two main types. If no variable gaps are allowed in the motif, it is called a *simple motif*. For example, in the genome of *Saccharomyces cerevisiae*, the binding sites of transcription factor, GAL4 [1], can be characterized by the simple motif shown in Table 1, which illustrates the pattern over the IUPAC alphabet (Σ_IUPAC_; see Table 2), as well as its profile (which gives the frequency of each DNA base at each position). The motif in Table 1 only consists of one component and thus is a simple motif. Since the symbols in the first 3 positions (CGS) and in the last 3 positions (SCG) are well conserved, we can also represent this motif as CGS[11,11]SCG, where [11,11] means that there is a fixed "gap" of length 11 between the two components. If variable gaps are allowed in a motif, it is called a *structured motif*. A structured motif can be regarded as an ordered collection of simple motifs with gap constraints between each pair of adjacent simple motifs. For example, the LTR retrotransposons from the *Copia *group, corresponding to genes encoding *reverse transcriptase*, in *A. thaliana *can be characterized by the structured motif *M*_1 _[2,5] *M*_2 _[6,7] *M*_3_, as shown in Table 3[2]. Here *M*_1_, *M*_2 _and *M*_3 _are three simple motifs; [2,5] and [6,7] are variable gap constraints ([minimum gap, maximum gap]) allowed between the adjacent simple motifs. Note that each simple motif *M*_*i *_(with 1 ≤ *i *≤ 3) can either be a pattern over Σ_IUPAC _or a profile over Σ_DNA_. Searching for structured motifs is more complicated than searching for simple motifs, and is an ongoing research area [3-7]. The sequence to be searched can be very long, e.g., chromosome 1 of *Homo Sapiens *contains 245 million (245M) base pairs. The structured motif can also be as long as several kilobases. All these factors need to be considered when designing an efficient structured motif search algorithm.

**Table 1:** A Simple Motif

**Symbols**	**Motif**																
A	0	0	0	4	1	1	7	0	5	1	0	2	0	2	0	0	0
C	10	0	1	2	3	5	0	7	0	4	2	5	5	1	9	10	0
G	0	10	9	4	5	3	2	3	0	3	1	1	4	1	1	0	10
T	0	0	0	0	1	1	1	0	5	2	7	2	1	6	0	0	0

IUPAC	C	G	S	V	N	N	D	S	W	N	B	N	B	N	S	C	G

The binding sites for the transcription factor GAL4 in *S. cerevisiae *satisfy this motif [1]. Rows 2–5 show the profile (the frequency of observing a DNA base in a given position). The last row shows the corresponding pattern over the IUPAC alphabet.

**Table 2:** IUPAC Alphabet (Σ_IUPAC_)

Symbol	A	C	G	T	U	R	Y	K
Bases	A	C	G	T	U	A,G	C,T	G,T
Symbol	M	S	W	B	D	H	V	N
Bases	A,C	G,C	A,T	C,G,T	A,G,T	A,C,T	A,C,G	A,C,G,T

**Table 3:** A Structured Motif

**Symbols**	*M*_1_										*M*_2_			*M*_3_		

A	2	12	17	1	11	1	35	0	24		1	0		3	1	35
C	0	10	8	5	2	0	0	19	0		0	25		5	35	1
G	2	5	5	2	10	34	1	0	0		26	11		0	0	0
T	32	9	6	28	13	1	0	17	12		9	0		28	0	0

IUPAC	D	N	N	N	N	D	R	Y	W		D	S		H	M	M

We aligned the 36 *A. thaliana *LTR retrotransposons from Repbase Update [2] database which belong to *Copia *group corresponding to genes encoding *reverse transcriptase *to obtain the structured motif *M*_1 _[2,5] *M*_2 _[6,7] *M*_3_. Rows 2–5 show the profile (the frequency of observing a DNA base in a given position), and the last row shows the corresponding pattern over the IUPAC alphabet.

More formally, a structured motif ℳ
 MathType@MTEF@5@5@+=feaafiart1ev1aaatCvAUfKttLearuWrP9MDH5MBPbIqV92AaeXatLxBI9gBamrtHrhAL1wy0L2yHvtyaeHbnfgDOvwBHrxAJfwnaebbnrfifHhDYfgasaacH8akY=wiFfYdH8Gipec8Eeeu0xXdbba9frFj0=OqFfea0dXdd9vqai=hGuQ8kuc9pgc9s8qqaq=dirpe0xb9q8qiLsFr0=vr0=vr0dc8meaabaqaciaacaGaaeqabaWaaeGaeaaakeaaimaacqWFZestaaa@3790@, is specified in the form:  *M*_1 _[*l*_1_, *u*_1_] *M*_2 _[*l*_2_, *u*_2_] *M*_3 _... *M*_*k*-1 _[*l*_*k*-1_, *u*_*k*-1_] *M*_*k*_, where *M*_*i*_, 1 ≤ *i *≤ *k*, is a simple motif *component*; and *l*_*i *_and *u*_*i *_(with 0 ≤ *l*_*i *_≤ *u*_*i*_), 1 ≤ *i *<*k*, are the minimum and maximum length of the gap allowed between *M*_*i *_and *M*_*i*+1_, respectively. Note that a gap is defined to be the number of intervening positions after *M*_*i *_but before *M*_*i*+1_. In other words if *s*_*i *_and *e*_*i *_represent the start and end positions of component *M*_*i*_, then for *i *∈ [1, *k *- 1], the length of the gap between *M*_*i *_and *M*_*i*+1 _is given as *g*_*i *_= *e*_*i*+1 _- *s*_*i *_- 1, and we require that *g*_*i *_∈ [*l*_*i*_, *u*_*i*_]. We use |*M*_*i*_| to denote the number of symbols/positions in component *M*_*i*_, also called the *length *of the component, and we use |ℳ|=∑i=1k|ℳi|
 MathType@MTEF@5@5@+=feaafiart1ev1aaatCvAUfKttLearuWrP9MDH5MBPbIqV92AaeXatLxBI9gBamrtHrhAL1wy0L2yHvtyaeHbnfgDOvwBHrxAJfwnaebbnrfifHhDYfgasaacH8akY=wiFfYdH8Gipec8Eeeu0xXdbba9frFj0=OqFfea0dXdd9vqai=hGuQ8kuc9pgc9s8qqaq=dirpe0xb9q8qiLsFr0=vr0=vr0dc8meaabaqaciaacaGaaeqabaWaaeGaeaaakeaacqGG8baFimaacqWFZestcqGG8baFcqGH9aqpdaaeWaqaaiabcYha8jab=ntinnaaBaaaleaacqWGPbqAaeqaaOGaeiiFaWhaleaacqWGPbqAcqGH9aqpcqaIXaqmaeaacqWGRbWAa0GaeyyeIuoaaaa@47FF@ to denote the total *length *of the structured motif ℳ
 MathType@MTEF@5@5@+=feaafiart1ev1aaatCvAUfKttLearuWrP9MDH5MBPbIqV92AaeXatLxBI9gBamrtHrhAL1wy0L2yHvtyaeHbnfgDOvwBHrxAJfwnaebbnrfifHhDYfgasaacH8akY=wiFfYdH8Gipec8Eeeu0xXdbba9frFj0=OqFfea0dXdd9vqai=hGuQ8kuc9pgc9s8qqaq=dirpe0xb9q8qiLsFr0=vr0=vr0dc8meaabaqaciaacaGaaeqabaWaaeGaeaaakeaaimaacqWFZestaaa@3790@ (not counting gaps). The *maximum span*, *L*, of the motif ℳ
 MathType@MTEF@5@5@+=feaafiart1ev1aaatCvAUfKttLearuWrP9MDH5MBPbIqV92AaeXatLxBI9gBamrtHrhAL1wy0L2yHvtyaeHbnfgDOvwBHrxAJfwnaebbnrfifHhDYfgasaacH8akY=wiFfYdH8Gipec8Eeeu0xXdbba9frFj0=OqFfea0dXdd9vqai=hGuQ8kuc9pgc9s8qqaq=dirpe0xb9q8qiLsFr0=vr0=vr0dc8meaabaqaciaacaGaaeqabaWaaeGaeaaakeaaimaacqWFZestaaa@3790@ is the maximum number of positions that can be occupied by the structured motif, which is given as L=∑i=1k|Mi|+∑i=1k−1ui
 MathType@MTEF@5@5@+=feaafiart1ev1aaatCvAUfKttLearuWrP9MDH5MBPbIqV92AaeXatLxBI9gBaebbnrfifHhDYfgasaacH8akY=wiFfYdH8Gipec8Eeeu0xXdbba9frFj0=OqFfea0dXdd9vqai=hGuQ8kuc9pgc9s8qqaq=dirpe0xb9q8qiLsFr0=vr0=vr0dc8meaabaqaciaacaGaaeqabaqabeGadaaakeaacqWGmbatcqGH9aqpdaaeWaqaaiabcYha8jabd2eannaaBaaaleaacqWGPbqAaeqaaOGaeiiFaWNaey4kaSYaaabmaeaacqWG1bqDdaWgaaWcbaGaemyAaKgabeaaaeaacqWGPbqAcqGH9aqpcqaIXaqmaeaacqWGRbWAcqGHsislcqaIXaqma0GaeyyeIuoaaSqaaiabdMgaPjabg2da9iabigdaXaqaaiabdUgaRbqdcqGHris5aaaa@4799@. The structured motif ℳ
 MathType@MTEF@5@5@+=feaafiart1ev1aaatCvAUfKttLearuWrP9MDH5MBPbIqV92AaeXatLxBI9gBamrtHrhAL1wy0L2yHvtyaeHbnfgDOvwBHrxAJfwnaebbnrfifHhDYfgasaacH8akY=wiFfYdH8Gipec8Eeeu0xXdbba9frFj0=OqFfea0dXdd9vqai=hGuQ8kuc9pgc9s8qqaq=dirpe0xb9q8qiLsFr0=vr0=vr0dc8meaabaqaciaacaGaaeqabaWaaeGaeaaakeaaimaacqWFZestaaa@3790@ can be either specified as a pattern or a profile. When ℳ
 MathType@MTEF@5@5@+=feaafiart1ev1aaatCvAUfKttLearuWrP9MDH5MBPbIqV92AaeXatLxBI9gBamrtHrhAL1wy0L2yHvtyaeHbnfgDOvwBHrxAJfwnaebbnrfifHhDYfgasaacH8akY=wiFfYdH8Gipec8Eeeu0xXdbba9frFj0=OqFfea0dXdd9vqai=hGuQ8kuc9pgc9s8qqaq=dirpe0xb9q8qiLsFr0=vr0=vr0dc8meaabaqaciaacaGaaeqabaWaaeGaeaaakeaaimaacqWFZestaaa@3790@ denotes a pattern, we use the notation ℳ
 MathType@MTEF@5@5@+=feaafiart1ev1aaatCvAUfKttLearuWrP9MDH5MBPbIqV92AaeXatLxBI9gBamrtHrhAL1wy0L2yHvtyaeHbnfgDOvwBHrxAJfwnaebbnrfifHhDYfgasaacH8akY=wiFfYdH8Gipec8Eeeu0xXdbba9frFj0=OqFfea0dXdd9vqai=hGuQ8kuc9pgc9s8qqaq=dirpe0xb9q8qiLsFr0=vr0=vr0dc8meaabaqaciaacaGaaeqabaWaaeGaeaaakeaaimaacqWFZestaaa@3790@_*j *_to denote the symbol at position *j *in the motif, and when ℳ
 MathType@MTEF@5@5@+=feaafiart1ev1aaatCvAUfKttLearuWrP9MDH5MBPbIqV92AaeXatLxBI9gBamrtHrhAL1wy0L2yHvtyaeHbnfgDOvwBHrxAJfwnaebbnrfifHhDYfgasaacH8akY=wiFfYdH8Gipec8Eeeu0xXdbba9frFj0=OqFfea0dXdd9vqai=hGuQ8kuc9pgc9s8qqaq=dirpe0xb9q8qiLsFr0=vr0=vr0dc8meaabaqaciaacaGaaeqabaWaaeGaeaaakeaaimaacqWFZestaaa@3790@ denotes a profile, we use the notation ℳ
 MathType@MTEF@5@5@+=feaafiart1ev1aaatCvAUfKttLearuWrP9MDH5MBPbIqV92AaeXatLxBI9gBamrtHrhAL1wy0L2yHvtyaeHbnfgDOvwBHrxAJfwnaebbnrfifHhDYfgasaacH8akY=wiFfYdH8Gipec8Eeeu0xXdbba9frFj0=OqFfea0dXdd9vqai=hGuQ8kuc9pgc9s8qqaq=dirpe0xb9q8qiLsFr0=vr0=vr0dc8meaabaqaciaacaGaaeqabaWaaeGaeaaakeaaimaacqWFZestaaa@3790@_*xj *_to denote the frequency of symbol *x *∈ Σ_DNA _at position *j*, where *j *∈ [1, |ℳ
 MathType@MTEF@5@5@+=feaafiart1ev1aaatCvAUfKttLearuWrP9MDH5MBPbIqV92AaeXatLxBI9gBamrtHrhAL1wy0L2yHvtyaeHbnfgDOvwBHrxAJfwnaebbnrfifHhDYfgasaacH8akY=wiFfYdH8Gipec8Eeeu0xXdbba9frFj0=OqFfea0dXdd9vqai=hGuQ8kuc9pgc9s8qqaq=dirpe0xb9q8qiLsFr0=vr0=vr0dc8meaabaqaciaacaGaaeqabaWaaeGaeaaakeaaimaacqWFZestaaa@3790@|]. Table 3 shows both the pattern and profile representation of an example structured motif with three components.

Given a collection of sequences, S
 MathType@MTEF@5@5@+=feaafiart1ev1aaatCvAUfKttLearuWrP9MDH5MBPbIqV92AaeXatLxBI9gBamrtHrhAL1wy0L2yHvtyaeHbnfgDOvwBHrxAJfwnaebbnrfifHhDYfgasaacH8akY=wiFfYdH8Gipec8Eeeu0xXdbba9frFj0=OqFfea0dXdd9vqai=hGuQ8kuc9pgc9s8qqaq=dirpe0xb9q8qiLsFr0=vr0=vr0dc8meaabaqaciaacaGaaeqabaWaaeGaeaaakeaaimaacqWFse=uaaa@3845@, over the DNA alphabet Σ_DNA _= {A,C,G,T}, and a structured motif, ℳ
 MathType@MTEF@5@5@+=feaafiart1ev1aaatCvAUfKttLearuWrP9MDH5MBPbIqV92AaeXatLxBI9gBamrtHrhAL1wy0L2yHvtyaeHbnfgDOvwBHrxAJfwnaebbnrfifHhDYfgasaacH8akY=wiFfYdH8Gipec8Eeeu0xXdbba9frFj0=OqFfea0dXdd9vqai=hGuQ8kuc9pgc9s8qqaq=dirpe0xb9q8qiLsFr0=vr0=vr0dc8meaabaqaciaacaGaaeqabaWaaeGaeaaakeaaimaacqWFZestaaa@3790@, the structured motif search problem is to report all the occurrences (or matches) of ℳ
 MathType@MTEF@5@5@+=feaafiart1ev1aaatCvAUfKttLearuWrP9MDH5MBPbIqV92AaeXatLxBI9gBamrtHrhAL1wy0L2yHvtyaeHbnfgDOvwBHrxAJfwnaebbnrfifHhDYfgasaacH8akY=wiFfYdH8Gipec8Eeeu0xXdbba9frFj0=OqFfea0dXdd9vqai=hGuQ8kuc9pgc9s8qqaq=dirpe0xb9q8qiLsFr0=vr0=vr0dc8meaabaqaciaacaGaaeqabaWaaeGaeaaakeaaimaacqWFZestaaa@3790@ in S
 MathType@MTEF@5@5@+=feaafiart1ev1aaatCvAUfKttLearuWrP9MDH5MBPbIqV92AaeXatLxBI9gBamrtHrhAL1wy0L2yHvtyaeHbnfgDOvwBHrxAJfwnaebbnrfifHhDYfgasaacH8akY=wiFfYdH8Gipec8Eeeu0xXdbba9frFj0=OqFfea0dXdd9vqai=hGuQ8kuc9pgc9s8qqaq=dirpe0xb9q8qiLsFr0=vr0=vr0dc8meaabaqaciaacaGaaeqabaWaaeGaeaaakeaaimaacqWFse=uaaa@3845@. The occurrence set of the structured motif, given as O
 MathType@MTEF@5@5@+=feaafiart1ev1aaatCvAUfKttLearuWrP9MDH5MBPbIqV92AaeXatLxBI9gBamrtHrhAL1wy0L2yHvtyaeHbnfgDOvwBHrxAJfwnaebbnrfifHhDYfgasaacH8akY=wiFfYdH8Gipec8Eeeu0xXdbba9frFj0=OqFfea0dXdd9vqai=hGuQ8kuc9pgc9s8qqaq=dirpe0xb9q8qiLsFr0=vr0=vr0dc8meaabaqaciaacaGaaeqabaWaaeGaeaaakeaaimaacqWFoe=taaa@383D@, can be reported in two forms: a) *full positions*: list of the positions for each symbol in ℳ
 MathType@MTEF@5@5@+=feaafiart1ev1aaatCvAUfKttLearuWrP9MDH5MBPbIqV92AaeXatLxBI9gBamrtHrhAL1wy0L2yHvtyaeHbnfgDOvwBHrxAJfwnaebbnrfifHhDYfgasaacH8akY=wiFfYdH8Gipec8Eeeu0xXdbba9frFj0=OqFfea0dXdd9vqai=hGuQ8kuc9pgc9s8qqaq=dirpe0xb9q8qiLsFr0=vr0=vr0dc8meaabaqaciaacaGaaeqabaWaaeGaeaaakeaaimaacqWFZestaaa@3790@, for all possible matches in S
 MathType@MTEF@5@5@+=feaafiart1ev1aaatCvAUfKttLearuWrP9MDH5MBPbIqV92AaeXatLxBI9gBamrtHrhAL1wy0L2yHvtyaeHbnfgDOvwBHrxAJfwnaebbnrfifHhDYfgasaacH8akY=wiFfYdH8Gipec8Eeeu0xXdbba9frFj0=OqFfea0dXdd9vqai=hGuQ8kuc9pgc9s8qqaq=dirpe0xb9q8qiLsFr0=vr0=vr0dc8meaabaqaciaacaGaaeqabaWaaeGaeaaakeaaimaacqWFse=uaaa@3845@, or b) *start positions*: list of the starting positions of ℳ
 MathType@MTEF@5@5@+=feaafiart1ev1aaatCvAUfKttLearuWrP9MDH5MBPbIqV92AaeXatLxBI9gBamrtHrhAL1wy0L2yHvtyaeHbnfgDOvwBHrxAJfwnaebbnrfifHhDYfgasaacH8akY=wiFfYdH8Gipec8Eeeu0xXdbba9frFj0=OqFfea0dXdd9vqai=hGuQ8kuc9pgc9s8qqaq=dirpe0xb9q8qiLsFr0=vr0=vr0dc8meaabaqaciaacaGaaeqabaWaaeGaeaaakeaaimaacqWFZestaaa@3790@ for each match in S
 MathType@MTEF@5@5@+=feaafiart1ev1aaatCvAUfKttLearuWrP9MDH5MBPbIqV92AaeXatLxBI9gBamrtHrhAL1wy0L2yHvtyaeHbnfgDOvwBHrxAJfwnaebbnrfifHhDYfgasaacH8akY=wiFfYdH8Gipec8Eeeu0xXdbba9frFj0=OqFfea0dXdd9vqai=hGuQ8kuc9pgc9s8qqaq=dirpe0xb9q8qiLsFr0=vr0=vr0dc8meaabaqaciaacaGaaeqabaWaaeGaeaaakeaaimaacqWFse=uaaa@3845@. Table 4 shows an example sequence S
 MathType@MTEF@5@5@+=feaafiart1ev1aaatCvAUfKttLearuWrP9MDH5MBPbIqV92AaeXatLxBI9gBamrtHrhAL1wy0L2yHvtyaeHbnfgDOvwBHrxAJfwnaebbnrfifHhDYfgasaacH8akY=wiFfYdH8Gipec8Eeeu0xXdbba9frFj0=OqFfea0dXdd9vqai=hGuQ8kuc9pgc9s8qqaq=dirpe0xb9q8qiLsFr0=vr0=vr0dc8meaabaqaciaacaGaaeqabaWaaeGaeaaakeaaimaacqWFse=uaaa@3845@ and a structured motif ℳ
 MathType@MTEF@5@5@+=feaafiart1ev1aaatCvAUfKttLearuWrP9MDH5MBPbIqV92AaeXatLxBI9gBamrtHrhAL1wy0L2yHvtyaeHbnfgDOvwBHrxAJfwnaebbnrfifHhDYfgasaacH8akY=wiFfYdH8Gipec8Eeeu0xXdbba9frFj0=OqFfea0dXdd9vqai=hGuQ8kuc9pgc9s8qqaq=dirpe0xb9q8qiLsFr0=vr0=vr0dc8meaabaqaciaacaGaaeqabaWaaeGaeaaakeaaimaacqWFZestaaa@3790@, where *M*_1 _= CG, *M*_2 _= TTA and *M*_3 _= CAT, and [0, 1] and [1,4] are the intervening gap ranges between *M*_1 _and *M*_2_, and *M*_2 _and *M*_3_, respectively. The motif has *k *= 3 components, it length is |ℳ
 MathType@MTEF@5@5@+=feaafiart1ev1aaatCvAUfKttLearuWrP9MDH5MBPbIqV92AaeXatLxBI9gBamrtHrhAL1wy0L2yHvtyaeHbnfgDOvwBHrxAJfwnaebbnrfifHhDYfgasaacH8akY=wiFfYdH8Gipec8Eeeu0xXdbba9frFj0=OqFfea0dXdd9vqai=hGuQ8kuc9pgc9s8qqaq=dirpe0xb9q8qiLsFr0=vr0=vr0dc8meaabaqaciaacaGaaeqabaWaaeGaeaaakeaaimaacqWFZestaaa@3790@| = 8 and its maximum span is *L *= 13. The occurrence set of full positions is O
 MathType@MTEF@5@5@+=feaafiart1ev1aaatCvAUfKttLearuWrP9MDH5MBPbIqV92AaeXatLxBI9gBamrtHrhAL1wy0L2yHvtyaeHbnfgDOvwBHrxAJfwnaebbnrfifHhDYfgasaacH8akY=wiFfYdH8Gipec8Eeeu0xXdbba9frFj0=OqFfea0dXdd9vqai=hGuQ8kuc9pgc9s8qqaq=dirpe0xb9q8qiLsFr0=vr0=vr0dc8meaabaqaciaacaGaaeqabaWaaeGaeaaakeaaimaacqWFoe=taaa@383D@ = {(5,6,8,9,10,12,13,14), (5,6,8,9,10,15,16,17)}, and of start positions is O
 MathType@MTEF@5@5@+=feaafiart1ev1aaatCvAUfKttLearuWrP9MDH5MBPbIqV92AaeXatLxBI9gBamrtHrhAL1wy0L2yHvtyaeHbnfgDOvwBHrxAJfwnaebbnrfifHhDYfgasaacH8akY=wiFfYdH8Gipec8Eeeu0xXdbba9frFj0=OqFfea0dXdd9vqai=hGuQ8kuc9pgc9s8qqaq=dirpe0xb9q8qiLsFr0=vr0=vr0dc8meaabaqaciaacaGaaeqabaWaaeGaeaaakeaaimaacqWFoe=taaa@383D@ = {5}.

Depending on the application, the structured motif search problem can have several variations:

**Table 4:** Structured Motif Search

Sequence (*S *∈ S MathType@MTEF@5@5@+=feaafiart1ev1aaatCvAUfKttLearuWrP9MDH5MBPbIqV92AaeXatLxBI9gBamrtHrhAL1wy0L2yHvtyaeHbnfgDOvwBHrxAJfwnaebbnrfifHhDYfgasaacH8akY=wiFfYdH8Gipec8Eeeu0xXdbba9frFj0=OqFfea0dXdd9vqai=hGuQ8kuc9pgc9s8qqaq=dirpe0xb9q8qiLsFr0=vr0=vr0dc8meaabaqaciaacaGaaeqabaWaaeGaeaaakeaaimaacqWFse=uaaa@3845@):	GCAT**GC**G**TTA**G**CATCAT**C
Structured Motif (ℳ MathType@MTEF@5@5@+=feaafiart1ev1aaatCvAUfKttLearuWrP9MDH5MBPbIqV92AaeXatLxBI9gBamrtHrhAL1wy0L2yHvtyaeHbnfgDOvwBHrxAJfwnaebbnrfifHhDYfgasaacH8akY=wiFfYdH8Gipec8Eeeu0xXdbba9frFj0=OqFfea0dXdd9vqai=hGuQ8kuc9pgc9s8qqaq=dirpe0xb9q8qiLsFr0=vr0=vr0dc8meaabaqaciaacaGaaeqabaWaaeGaeaaakeaaimaacqWFZestaaa@3790@):	GC[0,1]TTA[1,4]CAT

Occurrences of ℳ
 MathType@MTEF@5@5@+=feaafiart1ev1aaatCvAUfKttLearuWrP9MDH5MBPbIqV92AaeXatLxBI9gBamrtHrhAL1wy0L2yHvtyaeHbnfgDOvwBHrxAJfwnaebbnrfifHhDYfgasaacH8akY=wiFfYdH8Gipec8Eeeu0xXdbba9frFj0=OqFfea0dXdd9vqai=hGuQ8kuc9pgc9s8qqaq=dirpe0xb9q8qiLsFr0=vr0=vr0dc8meaabaqaciaacaGaaeqabaWaaeGaeaaakeaaimaacqWFZestaaa@3790@ in S
 MathType@MTEF@5@5@+=feaafiart1ev1aaatCvAUfKttLearuWrP9MDH5MBPbIqV92AaeXatLxBI9gBamrtHrhAL1wy0L2yHvtyaeHbnfgDOvwBHrxAJfwnaebbnrfifHhDYfgasaacH8akY=wiFfYdH8Gipec8Eeeu0xXdbba9frFj0=OqFfea0dXdd9vqai=hGuQ8kuc9pgc9s8qqaq=dirpe0xb9q8qiLsFr0=vr0=vr0dc8meaabaqaciaacaGaaeqabaWaaeGaeaaakeaaimaacqWFse=uaaa@3845@ are marked in bold.

• *Missing Components: *The matching motifs can consist of *some*, instead of *all*, the simple motifs in ℳ
 MathType@MTEF@5@5@+=feaafiart1ev1aaatCvAUfKttLearuWrP9MDH5MBPbIqV92AaeXatLxBI9gBamrtHrhAL1wy0L2yHvtyaeHbnfgDOvwBHrxAJfwnaebbnrfifHhDYfgasaacH8akY=wiFfYdH8Gipec8Eeeu0xXdbba9frFj0=OqFfea0dXdd9vqai=hGuQ8kuc9pgc9s8qqaq=dirpe0xb9q8qiLsFr0=vr0=vr0dc8meaabaqaciaacaGaaeqabaWaaeGaeaaakeaaimaacqWFZestaaa@3790@, allowing for at most *q *missing components.

• *Approximate Matches: *The matching motifs may consist of similar motifs (as measured by *Hamming *or *Levenshtein *distance [8]), instead of exact matches, to the simple motifs in ℳ
 MathType@MTEF@5@5@+=feaafiart1ev1aaatCvAUfKttLearuWrP9MDH5MBPbIqV92AaeXatLxBI9gBamrtHrhAL1wy0L2yHvtyaeHbnfgDOvwBHrxAJfwnaebbnrfifHhDYfgasaacH8akY=wiFfYdH8Gipec8Eeeu0xXdbba9frFj0=OqFfea0dXdd9vqai=hGuQ8kuc9pgc9s8qqaq=dirpe0xb9q8qiLsFr0=vr0=vr0dc8meaabaqaciaacaGaaeqabaWaaeGaeaaakeaaimaacqWFZestaaa@3790@, allowing for at most *ε*_*i *_errors for simple motif *M*_*i *_(when ℳ
 MathType@MTEF@5@5@+=feaafiart1ev1aaatCvAUfKttLearuWrP9MDH5MBPbIqV92AaeXatLxBI9gBamrtHrhAL1wy0L2yHvtyaeHbnfgDOvwBHrxAJfwnaebbnrfifHhDYfgasaacH8akY=wiFfYdH8Gipec8Eeeu0xXdbba9frFj0=OqFfea0dXdd9vqai=hGuQ8kuc9pgc9s8qqaq=dirpe0xb9q8qiLsFr0=vr0=vr0dc8meaabaqaciaacaGaaeqabaWaaeGaeaaakeaaimaacqWFZestaaa@3790@ is expressed as a pattern).

• *Overlapping Components: *The variable gap constraints (*l*_*i *_and *u*_*i*_) can take on a limited range of *negative *values, allowing search for overlapping simple motifs. We allow two adjacent components *M*_*i *_and *M*_*i*+1 _to overlap, but we require that *M*_*i*+1 _does not precede *M*_*i*_. This condition can be satisfied by the following constraints on the gap range [*l*_*i*_, *u*_*i*_]: - |*M*_*i*_| ≤ *l*_*i *_≤ *u*_*i*_, for *i *∈ [1, *k*). For example the search for motif ACG[-2,2]CGA, can discover the overlapped occurrence ACGA, as well as the non-overlapped occurrence ACG- -CGA, at the two extremes of the gap range.

• *Profile Search: *The components of the motif ℳ
 MathType@MTEF@5@5@+=feaafiart1ev1aaatCvAUfKttLearuWrP9MDH5MBPbIqV92AaeXatLxBI9gBamrtHrhAL1wy0L2yHvtyaeHbnfgDOvwBHrxAJfwnaebbnrfifHhDYfgasaacH8akY=wiFfYdH8Gipec8Eeeu0xXdbba9frFj0=OqFfea0dXdd9vqai=hGuQ8kuc9pgc9s8qqaq=dirpe0xb9q8qiLsFr0=vr0=vr0dc8meaabaqaciaacaGaaeqabaWaaeGaeaaakeaaimaacqWFZestaaa@3790@ can be specified as a pattern in either the DNA (Σ_DNA_) or IUPAC (Σ_IUPAC_) alphabets, or as a profile over Σ_DNA_.

In this paper, we focus on the problem of searching for a given structured motif in one or more sequences. We propose SMOTIF, an efficient algorithm for structured motif searches. It uses an inverted index of symbol positions, and it finds all occurrences by *positional joins *over this index. For structured pattern search problem, we propose two main variants of our approach: i) a direct search for simple motifs and the structured motif via positional joins, and ii) a two-step approach, where we use a suffix tree to search for simple motifs and then use positional joins for the structured motif. For structured profile search problem, we first search each simple motif by aligning its profile with the sequences, and then search structured motifs with positional joins. SMOTIF allows missing components, overlapping motifs, and also approximate matches (when using the two-step approach). SMOTIF also allows flexible matches using IUPAC symbols.

We apply SMOTIF for searching long DNA sequences for *LTR retrotransposons*, which constitute a substantial fraction of most Eukaryotic genomes and are believed to have a significant impact on genome structure and function [9,10]. We show that SMOTIF is effective in searching for composite regulatory patterns, and it can also suggest potentially new binding sites. We experimentally demonstrate the superiority of SMOTIF over SMARTFINDER, a state-of-the-art method for structured motif search, both in terms of time and space; SMOTIF can be up to 7 times faster and can consume 100 times less space.

### Related work

Many existing pattern matching algorithms [8,11-18] can be used to solve the simple pattern search problem. Given the sequence length, *n*, and the pattern length, *m*, exact matching algorithm can run in *O *(*n *+ *m*) [11]; approximate matching algorithm can run in *O *(*rn*) [16,17] or *O *(*nm/w*) [18], where *r *is the error threshold and *w *is the size of a computer word. The space complexity is *O *(*n*) for both exact matching and approximate matching.

Several previous efforts have focused on the structured pattern search problem. Anrep [3,4] provides a unified biosequence pattern representation by using *network expressions with spacers*, where a *network expression *is a regular expression without Kleene closure. With network expressions, one can specify the scoring scheme and the threshold of approximate matching for each simple motif separately, the positional weights which express the relative importance of different parts of a motif, and whether a simple motif is optional. Anrep introduces a two-step approach: first it searches for simple motifs by a threshold-sensitive motif matching algorithm and then it finds the structured motif by an optimized backtracking matching algorithm. However, as compared by [6], Anrep is much slower than SMARTFINDER.

In [5], the structured motif is called a *Classes of Characters and Bounded Gaps *(CBG) expression and is represented by a non-deterministic *ε*-automaton with bit parallelism. Two algorithms are proposed for CBG expression search: forward search and backward search. Bit parallelism speeds up the search, but is adequate only for a pattern whose maximum span is smaller than the length of the computer word. Also the implementation of CBG can only handle such pattern. This limits the application of CBG to searching for patterns with small number of symbols and gaps.

SMARTFINDER [6,7], which is currently the most efficient method for structured motif search, is also a two-step approach. In the first step each simple motif is searched separately by building a suffix tree for the sequence. This step outputs the ordered occurrence lists of all simple motifs. The second step solves a *constraint satisfaction problem *by considering constraints individually in three sub-steps. First it considers the gap constraints and builds a constraint graph whose nodes are the simple motif occurrences and edges connect all possible pairs of nodes that locally satisfy the gap constraints. It then considers the constraint for the maximum number of missing components and shrinks the graph to contain only the nodes that can be in the structured motif occurrences. Finally it enumerates all the valid occurrences by a depth first search (DFS). Notable differences in SMOTIF and SMARTFINDER are as follows: we search patterns directly by positional joins over an inverted index, we consider variable gap constraints during the positional joins as opposed to building a constraint graph, and we handle missing components more efficiently by considering them over *patterns *instead of over each *occurrence *as in SMARTFINDER. Note also that like Anrep and SMARTFINDER, SMOTIF can also mine approximate patterns, when using the two-step approach, which we describe later.

For profile search, MATCH [19], P-Match [20], and MatInspector [21,22] search DNA sequences against a position weight matrix library (such as TRANSFAC database [23]) and report the occurrences that satisfy given score thresholds. They compute the matrix score by multiplying the base frequency with the information content value at each position, in order to emphasize the fact that mismatches at less conserved positions are more easily tolerated than mismatches at highly conserved positions. Besides the matrix score, they define a *core *region, which is usually the first 4–5 most conserved consecutive positions of the matrix, and perform the core score threshold check. Then they align the matrix to each position of the sequence and calculate the core score and matrix score. However, these algorithm don't consider the prior probability of each base when calculating the matrix (or core) score, and the core region is required to be consecutive. They need to check all positions of each subsequence (at least all the core positions) in order to calculate the matrix (core) score. Moreover, these algorithms only work on simple profile with one single matrix component. For structured profile search, only Anrep [3,4] provides the capability to model structured profiles, with its general network expressions. However, Anrep doesn't give a solution on score calculation and fast search for structured profiles. Moreover, its implementation doesn't support structured profile search. To our knowledge, SMOTIF is the only implemented method that can handle structured profile search.

## Methods

We first introduce our basic approach for structured pattern search, and successively optimize it for various practical scenarios. We then present our approach for structured profile search.

### Structured pattern search: basic approach

Let us assume that we are searching for a structured motif ℳ
 MathType@MTEF@5@5@+=feaafiart1ev1aaatCvAUfKttLearuWrP9MDH5MBPbIqV92AaeXatLxBI9gBamrtHrhAL1wy0L2yHvtyaeHbnfgDOvwBHrxAJfwnaebbnrfifHhDYfgasaacH8akY=wiFfYdH8Gipec8Eeeu0xXdbba9frFj0=OqFfea0dXdd9vqai=hGuQ8kuc9pgc9s8qqaq=dirpe0xb9q8qiLsFr0=vr0=vr0dc8meaabaqaciaacaGaaeqabaWaaeGaeaaakeaaimaacqWFZestaaa@3790@ over a single sequence *S *∈ S
 MathType@MTEF@5@5@+=feaafiart1ev1aaatCvAUfKttLearuWrP9MDH5MBPbIqV92AaeXatLxBI9gBamrtHrhAL1wy0L2yHvtyaeHbnfgDOvwBHrxAJfwnaebbnrfifHhDYfgasaacH8akY=wiFfYdH8Gipec8Eeeu0xXdbba9frFj0=OqFfea0dXdd9vqai=hGuQ8kuc9pgc9s8qqaq=dirpe0xb9q8qiLsFr0=vr0=vr0dc8meaabaqaciaacaGaaeqabaWaaeGaeaaakeaaimaacqWFse=uaaa@3845@. We assume that *S *is over Σ_DNA_, whereas, ℳ
 MathType@MTEF@5@5@+=feaafiart1ev1aaatCvAUfKttLearuWrP9MDH5MBPbIqV92AaeXatLxBI9gBamrtHrhAL1wy0L2yHvtyaeHbnfgDOvwBHrxAJfwnaebbnrfifHhDYfgasaacH8akY=wiFfYdH8Gipec8Eeeu0xXdbba9frFj0=OqFfea0dXdd9vqai=hGuQ8kuc9pgc9s8qqaq=dirpe0xb9q8qiLsFr0=vr0=vr0dc8meaabaqaciaacaGaaeqabaWaaeGaeaaakeaaimaacqWFZestaaa@3790@ is over Σ_IUPAC _to allow for more flexible matches. SMOTIF first converts *S *into an equivalent inverted format [24,25], where we associate with each symbol in the sequence its *pos-list*, a *sorted *list of the positions where the symbol occurs in *S*. More formally, for a symbol *X *∈ Σ_IUPAC_, its pos-list is given as P
 MathType@MTEF@5@5@+=feaafiart1ev1aaatCvAUfKttLearuWrP9MDH5MBPbIqV92AaeXatLxBI9gBamrtHrhAL1wy0L2yHvtyaeHbnfgDOvwBHrxAJfwnaebbnrfifHhDYfgasaacH8akY=wiFfYdH8Gipec8Eeeu0xXdbba9frFj0=OqFfea0dXdd9vqai=hGuQ8kuc9pgc9s8qqaq=dirpe0xb9q8qiLsFr0=vr0=vr0dc8meaabaqaciaacaGaaeqabaWaaeGaeaaakeaacqqGqbauaaa@3786@(*X, S*) = {*i *| *S *[*i*] = *X*, *i *∈ [1, |*S*|]}, where *S *[*i*] is the symbol at position *i *in *S*, and |*S*| denotes the length of *S*. When *S *is obvious, we drop it, and denote the pos-list as P
 MathType@MTEF@5@5@+=feaafiart1ev1aaatCvAUfKttLearuWrP9MDH5MBPbIqV92AaeXatLxBI9gBamrtHrhAL1wy0L2yHvtyaeHbnfgDOvwBHrxAJfwnaebbnrfifHhDYfgasaacH8akY=wiFfYdH8Gipec8Eeeu0xXdbba9frFj0=OqFfea0dXdd9vqai=hGuQ8kuc9pgc9s8qqaq=dirpe0xb9q8qiLsFr0=vr0=vr0dc8meaabaqaciaacaGaaeqabaWaaeGaeaaakeaacqqGqbauaaa@3786@(*X*). For our example sequence *S *in Table 4, the pos-lists for *X *∈ Σ_DNA _are given in Table 5.

Depending on whether we compute the pos-lists for IUPAC symbols or not, SMOTIF uses two approaches: (a) *DNA pos-lists*: Here we keep (in memory) the pos-lists only for the four DNA symbols. For the other IUPAC symbols, we obtain their pos-lists by taking a union over the pos-lists of their constituting DNA symbols, e.g., P
 MathType@MTEF@5@5@+=feaafiart1ev1aaatCvAUfKttLearuWrP9MDH5MBPbIqV92AaeXatLxBI9gBamrtHrhAL1wy0L2yHvtyaeHbnfgDOvwBHrxAJfwnaebbnrfifHhDYfgasaacH8akY=wiFfYdH8Gipec8Eeeu0xXdbba9frFj0=OqFfea0dXdd9vqai=hGuQ8kuc9pgc9s8qqaq=dirpe0xb9q8qiLsFr0=vr0=vr0dc8meaabaqaciaacaGaaeqabaWaaeGaeaaakeaacqqGqbauaaa@3786@(*R*) = P
 MathType@MTEF@5@5@+=feaafiart1ev1aaatCvAUfKttLearuWrP9MDH5MBPbIqV92AaeXatLxBI9gBamrtHrhAL1wy0L2yHvtyaeHbnfgDOvwBHrxAJfwnaebbnrfifHhDYfgasaacH8akY=wiFfYdH8Gipec8Eeeu0xXdbba9frFj0=OqFfea0dXdd9vqai=hGuQ8kuc9pgc9s8qqaq=dirpe0xb9q8qiLsFr0=vr0=vr0dc8meaabaqaciaacaGaaeqabaWaaeGaeaaakeaacqqGqbauaaa@3786@(*A*) ∪ P
 MathType@MTEF@5@5@+=feaafiart1ev1aaatCvAUfKttLearuWrP9MDH5MBPbIqV92AaeXatLxBI9gBamrtHrhAL1wy0L2yHvtyaeHbnfgDOvwBHrxAJfwnaebbnrfifHhDYfgasaacH8akY=wiFfYdH8Gipec8Eeeu0xXdbba9frFj0=OqFfea0dXdd9vqai=hGuQ8kuc9pgc9s8qqaq=dirpe0xb9q8qiLsFr0=vr0=vr0dc8meaabaqaciaacaGaaeqabaWaaeGaeaaakeaacqqGqbauaaa@3786@(*G*) = {1, 3, 5, 7, 10, 11, 13, 16}. (b) *IUPAC pos-lists*: Here we keep (in memory) the pos-lists for the IUPAC symbols that actually appear in ℳ
 MathType@MTEF@5@5@+=feaafiart1ev1aaatCvAUfKttLearuWrP9MDH5MBPbIqV92AaeXatLxBI9gBamrtHrhAL1wy0L2yHvtyaeHbnfgDOvwBHrxAJfwnaebbnrfifHhDYfgasaacH8akY=wiFfYdH8Gipec8Eeeu0xXdbba9frFj0=OqFfea0dXdd9vqai=hGuQ8kuc9pgc9s8qqaq=dirpe0xb9q8qiLsFr0=vr0=vr0dc8meaabaqaciaacaGaaeqabaWaaeGaeaaakeaaimaacqWFZestaaa@3790@. These pos-lists are computed directly by scanning *S *once.

**Table 5:** Pos-lists

A	C	G	T
3	2	1	4
10	6	5	8
13	12	7	9
16	15	11	14
	18		17

#### Positional joins

**Figure 1:**
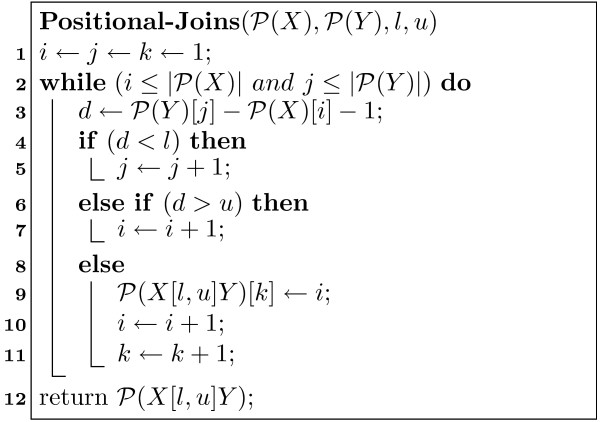
Positional Joins Algorithm.

We first extend the notion of pos-lists to cover structured motifs. The pos-list of ℳ
 MathType@MTEF@5@5@+=feaafiart1ev1aaatCvAUfKttLearuWrP9MDH5MBPbIqV92AaeXatLxBI9gBamrtHrhAL1wy0L2yHvtyaeHbnfgDOvwBHrxAJfwnaebbnrfifHhDYfgasaacH8akY=wiFfYdH8Gipec8Eeeu0xXdbba9frFj0=OqFfea0dXdd9vqai=hGuQ8kuc9pgc9s8qqaq=dirpe0xb9q8qiLsFr0=vr0=vr0dc8meaabaqaciaacaGaaeqabaWaaeGaeaaakeaaimaacqWFZestaaa@3790@ is given as the set of start positions of all the matches of ℳ
 MathType@MTEF@5@5@+=feaafiart1ev1aaatCvAUfKttLearuWrP9MDH5MBPbIqV92AaeXatLxBI9gBamrtHrhAL1wy0L2yHvtyaeHbnfgDOvwBHrxAJfwnaebbnrfifHhDYfgasaacH8akY=wiFfYdH8Gipec8Eeeu0xXdbba9frFj0=OqFfea0dXdd9vqai=hGuQ8kuc9pgc9s8qqaq=dirpe0xb9q8qiLsFr0=vr0=vr0dc8meaabaqaciaacaGaaeqabaWaaeGaeaaakeaaimaacqWFZestaaa@3790@ in *S*. Let *X*, *Y *∈ Σ_IUPAC _be any two symbols, and let ℳ
 MathType@MTEF@5@5@+=feaafiart1ev1aaatCvAUfKttLearuWrP9MDH5MBPbIqV92AaeXatLxBI9gBamrtHrhAL1wy0L2yHvtyaeHbnfgDOvwBHrxAJfwnaebbnrfifHhDYfgasaacH8akY=wiFfYdH8Gipec8Eeeu0xXdbba9frFj0=OqFfea0dXdd9vqai=hGuQ8kuc9pgc9s8qqaq=dirpe0xb9q8qiLsFr0=vr0=vr0dc8meaabaqaciaacaGaaeqabaWaaeGaeaaakeaaimaacqWFZestaaa@3790@ = *X *[*l*, *u*] *Y *be a structured motif. Given the pos-lists of *X *and *Y*, namely, P
 MathType@MTEF@5@5@+=feaafiart1ev1aaatCvAUfKttLearuWrP9MDH5MBPbIqV92AaeXatLxBI9gBamrtHrhAL1wy0L2yHvtyaeHbnfgDOvwBHrxAJfwnaebbnrfifHhDYfgasaacH8akY=wiFfYdH8Gipec8Eeeu0xXdbba9frFj0=OqFfea0dXdd9vqai=hGuQ8kuc9pgc9s8qqaq=dirpe0xb9q8qiLsFr0=vr0=vr0dc8meaabaqaciaacaGaaeqabaWaaeGaeaaakeaacqqGqbauaaa@3786@(*X*) and P
 MathType@MTEF@5@5@+=feaafiart1ev1aaatCvAUfKttLearuWrP9MDH5MBPbIqV92AaeXatLxBI9gBamrtHrhAL1wy0L2yHvtyaeHbnfgDOvwBHrxAJfwnaebbnrfifHhDYfgasaacH8akY=wiFfYdH8Gipec8Eeeu0xXdbba9frFj0=OqFfea0dXdd9vqai=hGuQ8kuc9pgc9s8qqaq=dirpe0xb9q8qiLsFr0=vr0=vr0dc8meaabaqaciaacaGaaeqabaWaaeGaeaaakeaacqqGqbauaaa@3786@(*Y*), the pos-list for ℳ
 MathType@MTEF@5@5@+=feaafiart1ev1aaatCvAUfKttLearuWrP9MDH5MBPbIqV92AaeXatLxBI9gBamrtHrhAL1wy0L2yHvtyaeHbnfgDOvwBHrxAJfwnaebbnrfifHhDYfgasaacH8akY=wiFfYdH8Gipec8Eeeu0xXdbba9frFj0=OqFfea0dXdd9vqai=hGuQ8kuc9pgc9s8qqaq=dirpe0xb9q8qiLsFr0=vr0=vr0dc8meaabaqaciaacaGaaeqabaWaaeGaeaaakeaaimaacqWFZestaaa@3790@ can be obtained by a positional join as follows: For a position *x *∈ P
 MathType@MTEF@5@5@+=feaafiart1ev1aaatCvAUfKttLearuWrP9MDH5MBPbIqV92AaeXatLxBI9gBamrtHrhAL1wy0L2yHvtyaeHbnfgDOvwBHrxAJfwnaebbnrfifHhDYfgasaacH8akY=wiFfYdH8Gipec8Eeeu0xXdbba9frFj0=OqFfea0dXdd9vqai=hGuQ8kuc9pgc9s8qqaq=dirpe0xb9q8qiLsFr0=vr0=vr0dc8meaabaqaciaacaGaaeqabaWaaeGaeaaakeaacqqGqbauaaa@3786@(*X*), if there exists a position *y *∈ P
 MathType@MTEF@5@5@+=feaafiart1ev1aaatCvAUfKttLearuWrP9MDH5MBPbIqV92AaeXatLxBI9gBamrtHrhAL1wy0L2yHvtyaeHbnfgDOvwBHrxAJfwnaebbnrfifHhDYfgasaacH8akY=wiFfYdH8Gipec8Eeeu0xXdbba9frFj0=OqFfea0dXdd9vqai=hGuQ8kuc9pgc9s8qqaq=dirpe0xb9q8qiLsFr0=vr0=vr0dc8meaabaqaciaacaGaaeqabaWaaeGaeaaakeaacqqGqbauaaa@3786@(*Y*), such that *l *≤ *y *- *x *- 1 ≤ *u*, it means that *Y *follows *X *within the variable gap range [*l*, *u*] in the sequence *S*, and thus we can add *x *to the pos-list of motif *X *[*l*, *u*] *Y*. Let *d *be the length of the gap between *x *∈ P
 MathType@MTEF@5@5@+=feaafiart1ev1aaatCvAUfKttLearuWrP9MDH5MBPbIqV92AaeXatLxBI9gBamrtHrhAL1wy0L2yHvtyaeHbnfgDOvwBHrxAJfwnaebbnrfifHhDYfgasaacH8akY=wiFfYdH8Gipec8Eeeu0xXdbba9frFj0=OqFfea0dXdd9vqai=hGuQ8kuc9pgc9s8qqaq=dirpe0xb9q8qiLsFr0=vr0=vr0dc8meaabaqaciaacaGaaeqabaWaaeGaeaaakeaacqqGqbauaaa@3786@(*X*) and *y *∈ P
 MathType@MTEF@5@5@+=feaafiart1ev1aaatCvAUfKttLearuWrP9MDH5MBPbIqV92AaeXatLxBI9gBamrtHrhAL1wy0L2yHvtyaeHbnfgDOvwBHrxAJfwnaebbnrfifHhDYfgasaacH8akY=wiFfYdH8Gipec8Eeeu0xXdbba9frFj0=OqFfea0dXdd9vqai=hGuQ8kuc9pgc9s8qqaq=dirpe0xb9q8qiLsFr0=vr0=vr0dc8meaabaqaciaacaGaaeqabaWaaeGaeaaakeaacqqGqbauaaa@3786@(*Y*), given as *d *= *y *- *x *- 1. Then, in general, there are three cases to consider in the positional join algorithm, as shown in Figure 1,

• *d *<*l*: Advance *y *to the next element in P
 MathType@MTEF@5@5@+=feaafiart1ev1aaatCvAUfKttLearuWrP9MDH5MBPbIqV92AaeXatLxBI9gBamrtHrhAL1wy0L2yHvtyaeHbnfgDOvwBHrxAJfwnaebbnrfifHhDYfgasaacH8akY=wiFfYdH8Gipec8Eeeu0xXdbba9frFj0=OqFfea0dXdd9vqai=hGuQ8kuc9pgc9s8qqaq=dirpe0xb9q8qiLsFr0=vr0=vr0dc8meaabaqaciaacaGaaeqabaWaaeGaeaaakeaacqqGqbauaaa@3786@(*Y*).

• *d *> *u*: Advance *x *to the next element in P
 MathType@MTEF@5@5@+=feaafiart1ev1aaatCvAUfKttLearuWrP9MDH5MBPbIqV92AaeXatLxBI9gBamrtHrhAL1wy0L2yHvtyaeHbnfgDOvwBHrxAJfwnaebbnrfifHhDYfgasaacH8akY=wiFfYdH8Gipec8Eeeu0xXdbba9frFj0=OqFfea0dXdd9vqai=hGuQ8kuc9pgc9s8qqaq=dirpe0xb9q8qiLsFr0=vr0=vr0dc8meaabaqaciaacaGaaeqabaWaaeGaeaaakeaacqqGqbauaaa@3786@(*X*).

• *l *≤ *d *≤ *u*: Save this occurrence in P
 MathType@MTEF@5@5@+=feaafiart1ev1aaatCvAUfKttLearuWrP9MDH5MBPbIqV92AaeXatLxBI9gBamrtHrhAL1wy0L2yHvtyaeHbnfgDOvwBHrxAJfwnaebbnrfifHhDYfgasaacH8akY=wiFfYdH8Gipec8Eeeu0xXdbba9frFj0=OqFfea0dXdd9vqai=hGuQ8kuc9pgc9s8qqaq=dirpe0xb9q8qiLsFr0=vr0=vr0dc8meaabaqaciaacaGaaeqabaWaaeGaeaaakeaacqqGqbauaaa@3786@(*X *[*l*, *u*] *Y*), and then advance *x*.

The pos-list for *X *[*l*, *u*] *Y *can be computed in time linear in the lengths of P
 MathType@MTEF@5@5@+=feaafiart1ev1aaatCvAUfKttLearuWrP9MDH5MBPbIqV92AaeXatLxBI9gBamrtHrhAL1wy0L2yHvtyaeHbnfgDOvwBHrxAJfwnaebbnrfifHhDYfgasaacH8akY=wiFfYdH8Gipec8Eeeu0xXdbba9frFj0=OqFfea0dXdd9vqai=hGuQ8kuc9pgc9s8qqaq=dirpe0xb9q8qiLsFr0=vr0=vr0dc8meaabaqaciaacaGaaeqabaWaaeGaeaaakeaacqqGqbauaaa@3786@(*X*) and P
 MathType@MTEF@5@5@+=feaafiart1ev1aaatCvAUfKttLearuWrP9MDH5MBPbIqV92AaeXatLxBI9gBamrtHrhAL1wy0L2yHvtyaeHbnfgDOvwBHrxAJfwnaebbnrfifHhDYfgasaacH8akY=wiFfYdH8Gipec8Eeeu0xXdbba9frFj0=OqFfea0dXdd9vqai=hGuQ8kuc9pgc9s8qqaq=dirpe0xb9q8qiLsFr0=vr0=vr0dc8meaabaqaciaacaGaaeqabaWaaeGaeaaakeaacqqGqbauaaa@3786@(*Y*). In essence, each time we advance *x *∈ P
 MathType@MTEF@5@5@+=feaafiart1ev1aaatCvAUfKttLearuWrP9MDH5MBPbIqV92AaeXatLxBI9gBamrtHrhAL1wy0L2yHvtyaeHbnfgDOvwBHrxAJfwnaebbnrfifHhDYfgasaacH8akY=wiFfYdH8Gipec8Eeeu0xXdbba9frFj0=OqFfea0dXdd9vqai=hGuQ8kuc9pgc9s8qqaq=dirpe0xb9q8qiLsFr0=vr0=vr0dc8meaabaqaciaacaGaaeqabaWaaeGaeaaakeaacqqGqbauaaa@3786@(*X*), we check if there exists a *y *∈ P
 MathType@MTEF@5@5@+=feaafiart1ev1aaatCvAUfKttLearuWrP9MDH5MBPbIqV92AaeXatLxBI9gBamrtHrhAL1wy0L2yHvtyaeHbnfgDOvwBHrxAJfwnaebbnrfifHhDYfgasaacH8akY=wiFfYdH8Gipec8Eeeu0xXdbba9frFj0=OqFfea0dXdd9vqai=hGuQ8kuc9pgc9s8qqaq=dirpe0xb9q8qiLsFr0=vr0=vr0dc8meaabaqaciaacaGaaeqabaWaaeGaeaaakeaacqqGqbauaaa@3786@(*Y*) that satisfies the given gap constraint. Instead of searching for the matching *y *from the beginning of the pos-list each time, we search from the last position used to compare with *x*. This results in fast positional joins, and also allows overlaps among occurrences. For example, during the positional join for the motif T[0, 1]A, with *l *= 0 and *u *= 1, we scan the pos-lists of T and A in Table 5. Initially, *x *= 4 and *y *= 3. This gives *d *= 3 - 4 - 1 = -2 <*l*, thus we advance *y *to 10. Next, *d *= 10 - 4 - 1 = 5 > *u*, thus we advance *x *to 8. Next, *d *= 10 - 8 - 1 = 1 ∈ [0, 1]. So we store *x *= 8 in P
 MathType@MTEF@5@5@+=feaafiart1ev1aaatCvAUfKttLearuWrP9MDH5MBPbIqV92AaeXatLxBI9gBamrtHrhAL1wy0L2yHvtyaeHbnfgDOvwBHrxAJfwnaebbnrfifHhDYfgasaacH8akY=wiFfYdH8Gipec8Eeeu0xXdbba9frFj0=OqFfea0dXdd9vqai=hGuQ8kuc9pgc9s8qqaq=dirpe0xb9q8qiLsFr0=vr0=vr0dc8meaabaqaciaacaGaaeqabaWaaeGaeaaakeaacqqGqbauaaa@3786@(*T*[0, 1] *A*) and advance *x *to 9. By continuing the process, we get the final pos-list as P
 MathType@MTEF@5@5@+=feaafiart1ev1aaatCvAUfKttLearuWrP9MDH5MBPbIqV92AaeXatLxBI9gBamrtHrhAL1wy0L2yHvtyaeHbnfgDOvwBHrxAJfwnaebbnrfifHhDYfgasaacH8akY=wiFfYdH8Gipec8Eeeu0xXdbba9frFj0=OqFfea0dXdd9vqai=hGuQ8kuc9pgc9s8qqaq=dirpe0xb9q8qiLsFr0=vr0=vr0dc8meaabaqaciaacaGaaeqabaWaaeGaeaaakeaacqqGqbauaaa@3786@(*T*[0,l] *A*) = {8, 9, 14}.

Given a longer motif ℳ
 MathType@MTEF@5@5@+=feaafiart1ev1aaatCvAUfKttLearuWrP9MDH5MBPbIqV92AaeXatLxBI9gBamrtHrhAL1wy0L2yHvtyaeHbnfgDOvwBHrxAJfwnaebbnrfifHhDYfgasaacH8akY=wiFfYdH8Gipec8Eeeu0xXdbba9frFj0=OqFfea0dXdd9vqai=hGuQ8kuc9pgc9s8qqaq=dirpe0xb9q8qiLsFr0=vr0=vr0dc8meaabaqaciaacaGaaeqabaWaaeGaeaaakeaaimaacqWFZestaaa@3790@, the positional joins start with the last two symbols, and proceed by successively joining the pos-list of the current symbol with the intermediate pos-list of the suffix. Formally, let *H *[*l*, *u*] *T *be an intermediate pattern, with symbol *H *as the *head symbol*, and a suffix structured motif *T *as *tail*. The pos-list of *H *[*l*, *u*] *T *is obtained by doing positional join on the pos-list of *H *and the pos-list of *T*. As the computation progresses the previous tail pos-lists are discarded. Combined with the fact that only start positions are kept in a pos-list, this saves both time and space.

SMOTIF handles both simple and structured motifs uniformly, by adding the gap range [0, 0] between adjacent symbols within each simple motif *M*_*i*_. For our example in Table 4, the structured motif ℳ
 MathType@MTEF@5@5@+=feaafiart1ev1aaatCvAUfKttLearuWrP9MDH5MBPbIqV92AaeXatLxBI9gBamrtHrhAL1wy0L2yHvtyaeHbnfgDOvwBHrxAJfwnaebbnrfifHhDYfgasaacH8akY=wiFfYdH8Gipec8Eeeu0xXdbba9frFj0=OqFfea0dXdd9vqai=hGuQ8kuc9pgc9s8qqaq=dirpe0xb9q8qiLsFr0=vr0=vr0dc8meaabaqaciaacaGaaeqabaWaaeGaeaaakeaaimaacqWFZestaaa@3790@ becomes: G[0,0]C[0,1]T[0,0]T[0,0]A[1,4]C[0,0]A[0,0]T. Furthermore, SMOTIF treats the IUPAC symbol N (which stands for any of the four bases: A,C,G,T) as a gap, [1,1], and merges it with adjacent gaps in the motif. For example, the motif A[0,0]N[0,0]N[0,0]C will be first converted to A[0, 0][1,1][0,0][1,1][0,0]C, and then the adjacent gaps will be combined to obtain A[2,2]C as the final motif.

**Figure 2:**
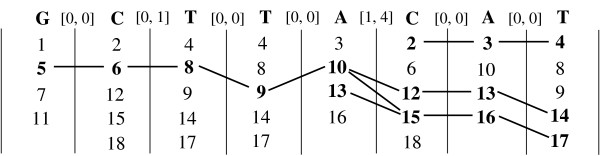
**Positional Joins Example**. The figure shows how the positional joins work for the (expanded) motif from Table 4.

Figure 2 shows how the positional joins work for our (expanded) motif from Table 4. At any stage in the process, the head symbol's pos-list corresponds to the full list of positions shown, whereas the tail's pos-list consists only of the positions shown in bold. For example, when computing A[1,4]CAT, the pos-list of the tail CAT is {2, 12, 15}, and that of the head symbol A is {3, 10, 13, 16}. Also, for illustration, we add a link between any two positions (*x *and *y*) in adjacent columns if their difference (*d *= *y *- *x *- 1) falls within the corresponding gap range. The joins begin with the last two symbols, with A as the head and T as the tail with a gap of [0,0]. The only positions *x *∈ P
 MathType@MTEF@5@5@+=feaafiart1ev1aaatCvAUfKttLearuWrP9MDH5MBPbIqV92AaeXatLxBI9gBamrtHrhAL1wy0L2yHvtyaeHbnfgDOvwBHrxAJfwnaebbnrfifHhDYfgasaacH8akY=wiFfYdH8Gipec8Eeeu0xXdbba9frFj0=OqFfea0dXdd9vqai=hGuQ8kuc9pgc9s8qqaq=dirpe0xb9q8qiLsFr0=vr0=vr0dc8meaabaqaciaacaGaaeqabaWaaeGaeaaakeaacqqGqbauaaa@3786@(*A*) that satisfy the adjacent gap constraint are 3, 13 and 16 (marked in bold), that form the pos-list of A[0,0]T. Next we join P
 MathType@MTEF@5@5@+=feaafiart1ev1aaatCvAUfKttLearuWrP9MDH5MBPbIqV92AaeXatLxBI9gBamrtHrhAL1wy0L2yHvtyaeHbnfgDOvwBHrxAJfwnaebbnrfifHhDYfgasaacH8akY=wiFfYdH8Gipec8Eeeu0xXdbba9frFj0=OqFfea0dXdd9vqai=hGuQ8kuc9pgc9s8qqaq=dirpe0xb9q8qiLsFr0=vr0=vr0dc8meaabaqaciaacaGaaeqabaWaaeGaeaaakeaacqqGqbauaaa@3786@(*C*) with P
 MathType@MTEF@5@5@+=feaafiart1ev1aaatCvAUfKttLearuWrP9MDH5MBPbIqV92AaeXatLxBI9gBamrtHrhAL1wy0L2yHvtyaeHbnfgDOvwBHrxAJfwnaebbnrfifHhDYfgasaacH8akY=wiFfYdH8Gipec8Eeeu0xXdbba9frFj0=OqFfea0dXdd9vqai=hGuQ8kuc9pgc9s8qqaq=dirpe0xb9q8qiLsFr0=vr0=vr0dc8meaabaqaciaacaGaaeqabaWaaeGaeaaakeaacqqGqbauaaa@3786@(*A*[0,0] *T*) to get the valid positions 2,12 and 15. At the next step we need to consider *A *as the head, with constraint [1,4], followed by the tail *C *[0,0] *A *[0,0] *T*. The only positions in P
 MathType@MTEF@5@5@+=feaafiart1ev1aaatCvAUfKttLearuWrP9MDH5MBPbIqV92AaeXatLxBI9gBamrtHrhAL1wy0L2yHvtyaeHbnfgDOvwBHrxAJfwnaebbnrfifHhDYfgasaacH8akY=wiFfYdH8Gipec8Eeeu0xXdbba9frFj0=OqFfea0dXdd9vqai=hGuQ8kuc9pgc9s8qqaq=dirpe0xb9q8qiLsFr0=vr0=vr0dc8meaabaqaciaacaGaaeqabaWaaeGaeaaakeaacqqGqbauaaa@3786@(*A*) that satisfy the gap constraint of *l *= 1 and *u *= 4 are 10 and 13. Note also how position 2 that was in the tail's pos-list cannot be extended, since there is no position where *A *occurs within a gap of [1,4] before the tail CAT. The join process continues until the first symbol, and we finally get 5 as the only start occurrence for the full structured motif.

#### Full position recovery

In our positional join approach, to save time and space we retain only the motif start positions, however, in some applications, we may need to know the full position of each occurrence, i.e., the set of matching positions for each symbol in the motif. We describe two approaches to recover the full positions: recomputed or indexed full-position recovery.

##### Recomputed full position recovery

**Figure 3:**
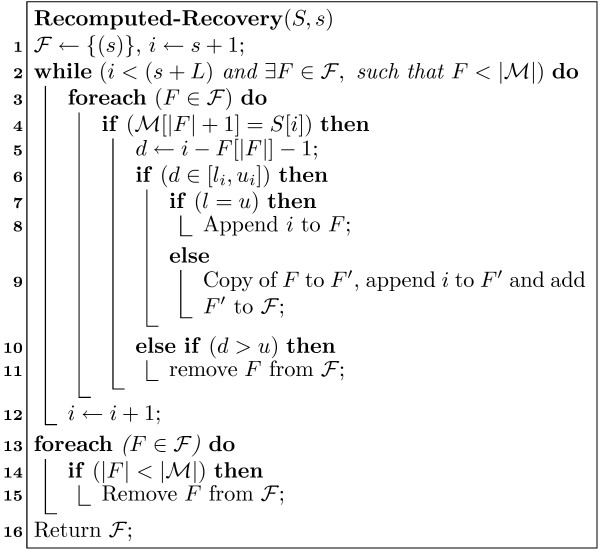
Recomputed Full Position Recovery Algorithm.

For recomputing the full positions SMOTIF needs access to only the sequence *S *and the post-list P
 MathType@MTEF@5@5@+=feaafiart1ev1aaatCvAUfKttLearuWrP9MDH5MBPbIqV92AaeXatLxBI9gBamrtHrhAL1wy0L2yHvtyaeHbnfgDOvwBHrxAJfwnaebbnrfifHhDYfgasaacH8akY=wiFfYdH8Gipec8Eeeu0xXdbba9frFj0=OqFfea0dXdd9vqai=hGuQ8kuc9pgc9s8qqaq=dirpe0xb9q8qiLsFr0=vr0=vr0dc8meaabaqaciaacaGaaeqabaWaaeGaeaaakeaacqqGqbauaaa@3786@(ℳ
 MathType@MTEF@5@5@+=feaafiart1ev1aaatCvAUfKttLearuWrP9MDH5MBPbIqV92AaeXatLxBI9gBamrtHrhAL1wy0L2yHvtyaeHbnfgDOvwBHrxAJfwnaebbnrfifHhDYfgasaacH8akY=wiFfYdH8Gipec8Eeeu0xXdbba9frFj0=OqFfea0dXdd9vqai=hGuQ8kuc9pgc9s8qqaq=dirpe0xb9q8qiLsFr0=vr0=vr0dc8meaabaqaciaacaGaaeqabaWaaeGaeaaakeaaimaacqWFZestaaa@3790@). Let *s *∈ P
 MathType@MTEF@5@5@+=feaafiart1ev1aaatCvAUfKttLearuWrP9MDH5MBPbIqV92AaeXatLxBI9gBamrtHrhAL1wy0L2yHvtyaeHbnfgDOvwBHrxAJfwnaebbnrfifHhDYfgasaacH8akY=wiFfYdH8Gipec8Eeeu0xXdbba9frFj0=OqFfea0dXdd9vqai=hGuQ8kuc9pgc9s8qqaq=dirpe0xb9q8qiLsFr0=vr0=vr0dc8meaabaqaciaacaGaaeqabaWaaeGaeaaakeaacqqGqbauaaa@3786@(ℳ
 MathType@MTEF@5@5@+=feaafiart1ev1aaatCvAUfKttLearuWrP9MDH5MBPbIqV92AaeXatLxBI9gBamrtHrhAL1wy0L2yHvtyaeHbnfgDOvwBHrxAJfwnaebbnrfifHhDYfgasaacH8akY=wiFfYdH8Gipec8Eeeu0xXdbba9frFj0=OqFfea0dXdd9vqai=hGuQ8kuc9pgc9s8qqaq=dirpe0xb9q8qiLsFr0=vr0=vr0dc8meaabaqaciaacaGaaeqabaWaaeGaeaaakeaaimaacqWFZestaaa@3790@) be a start position for the structured motif in sequence *S*. Figure 3 shows how to recompute the full positions starting from *s*. Note that a structured motif with maximum span *L *must be found within position range [*s*, *s *+ *L *- 1], so we can stop searching from *s *after the maximum span is reached. During the full position recovery, we maintain a list of intermediate position prefixes ℱ
 MathType@MTEF@5@5@+=feaafiart1ev1aaatCvAUfKttLearuWrP9MDH5MBPbIqV92AaeXatLxBI9gBamrtHrhAL1wy0L2yHvtyaeHbnfgDOvwBHrxAJfwnaebbnrfifHhDYfgasaacH8akY=wiFfYdH8Gipec8Eeeu0xXdbba9frFj0=OqFfea0dXdd9vqai=hGuQ8kuc9pgc9s8qqaq=dirpe0xb9q8qiLsFr0=vr0=vr0dc8meaabaqaciaacaGaaeqabaWaaeGaeaaakeaaimaacqWFXeIraaa@3787@ that match the prefix of ℳ
 MathType@MTEF@5@5@+=feaafiart1ev1aaatCvAUfKttLearuWrP9MDH5MBPbIqV92AaeXatLxBI9gBamrtHrhAL1wy0L2yHvtyaeHbnfgDOvwBHrxAJfwnaebbnrfifHhDYfgasaacH8akY=wiFfYdH8Gipec8Eeeu0xXdbba9frFj0=OqFfea0dXdd9vqai=hGuQ8kuc9pgc9s8qqaq=dirpe0xb9q8qiLsFr0=vr0=vr0dc8meaabaqaciaacaGaaeqabaWaaeGaeaaakeaaimaacqWFZestaaa@3790@. For an intermediate prefix *F *∈ ℱ
 MathType@MTEF@5@5@+=feaafiart1ev1aaatCvAUfKttLearuWrP9MDH5MBPbIqV92AaeXatLxBI9gBamrtHrhAL1wy0L2yHvtyaeHbnfgDOvwBHrxAJfwnaebbnrfifHhDYfgasaacH8akY=wiFfYdH8Gipec8Eeeu0xXdbba9frFj0=OqFfea0dXdd9vqai=hGuQ8kuc9pgc9s8qqaq=dirpe0xb9q8qiLsFr0=vr0=vr0dc8meaabaqaciaacaGaaeqabaWaaeGaeaaakeaaimaacqWFXeIraaa@3787@, let |*F*| denote the number of positions in *F*. Initially ℱ
 MathType@MTEF@5@5@+=feaafiart1ev1aaatCvAUfKttLearuWrP9MDH5MBPbIqV92AaeXatLxBI9gBamrtHrhAL1wy0L2yHvtyaeHbnfgDOvwBHrxAJfwnaebbnrfifHhDYfgasaacH8akY=wiFfYdH8Gipec8Eeeu0xXdbba9frFj0=OqFfea0dXdd9vqai=hGuQ8kuc9pgc9s8qqaq=dirpe0xb9q8qiLsFr0=vr0=vr0dc8meaabaqaciaacaGaaeqabaWaaeGaeaaakeaaimaacqWFXeIraaa@3787@ = {(s)}. For each symbol *S *[*i*] with *i *∈ [*s *+ 1, *s *+ *L *- 1] in sequence *S*, we consider each candidate prefix *F *∈ ℱ
 MathType@MTEF@5@5@+=feaafiart1ev1aaatCvAUfKttLearuWrP9MDH5MBPbIqV92AaeXatLxBI9gBamrtHrhAL1wy0L2yHvtyaeHbnfgDOvwBHrxAJfwnaebbnrfifHhDYfgasaacH8akY=wiFfYdH8Gipec8Eeeu0xXdbba9frFj0=OqFfea0dXdd9vqai=hGuQ8kuc9pgc9s8qqaq=dirpe0xb9q8qiLsFr0=vr0=vr0dc8meaabaqaciaacaGaaeqabaWaaeGaeaaakeaaimaacqWFXeIraaa@3787@ and check whether ℳ
 MathType@MTEF@5@5@+=feaafiart1ev1aaatCvAUfKttLearuWrP9MDH5MBPbIqV92AaeXatLxBI9gBamrtHrhAL1wy0L2yHvtyaeHbnfgDOvwBHrxAJfwnaebbnrfifHhDYfgasaacH8akY=wiFfYdH8Gipec8Eeeu0xXdbba9frFj0=OqFfea0dXdd9vqai=hGuQ8kuc9pgc9s8qqaq=dirpe0xb9q8qiLsFr0=vr0=vr0dc8meaabaqaciaacaGaaeqabaWaaeGaeaaakeaaimaacqWFZestaaa@3790@[|*F*| + 1] = *S *[*i*] and *d *= *i *- *F *[|*F*|] ∈ [*l*, *u*]. If yes, *S *[*i*] is a valid occurrence of ℳ
 MathType@MTEF@5@5@+=feaafiart1ev1aaatCvAUfKttLearuWrP9MDH5MBPbIqV92AaeXatLxBI9gBamrtHrhAL1wy0L2yHvtyaeHbnfgDOvwBHrxAJfwnaebbnrfifHhDYfgasaacH8akY=wiFfYdH8Gipec8Eeeu0xXdbba9frFj0=OqFfea0dXdd9vqai=hGuQ8kuc9pgc9s8qqaq=dirpe0xb9q8qiLsFr0=vr0=vr0dc8meaabaqaciaacaGaaeqabaWaaeGaeaaakeaaimaacqWFZestaaa@3790@[|*F*| + 1], and we append position *i *to *F*. If there are multiple positions *i *that are valid occurrences for ℳ
 MathType@MTEF@5@5@+=feaafiart1ev1aaatCvAUfKttLearuWrP9MDH5MBPbIqV92AaeXatLxBI9gBamrtHrhAL1wy0L2yHvtyaeHbnfgDOvwBHrxAJfwnaebbnrfifHhDYfgasaacH8akY=wiFfYdH8Gipec8Eeeu0xXdbba9frFj0=OqFfea0dXdd9vqai=hGuQ8kuc9pgc9s8qqaq=dirpe0xb9q8qiLsFr0=vr0=vr0dc8meaabaqaciaacaGaaeqabaWaaeGaeaaakeaaimaacqWFZestaaa@3790@[|*F*| + 1], we add as many copies of *F *appended with the *i *positions to ℱ
 MathType@MTEF@5@5@+=feaafiart1ev1aaatCvAUfKttLearuWrP9MDH5MBPbIqV92AaeXatLxBI9gBamrtHrhAL1wy0L2yHvtyaeHbnfgDOvwBHrxAJfwnaebbnrfifHhDYfgasaacH8akY=wiFfYdH8Gipec8Eeeu0xXdbba9frFj0=OqFfea0dXdd9vqai=hGuQ8kuc9pgc9s8qqaq=dirpe0xb9q8qiLsFr0=vr0=vr0dc8meaabaqaciaacaGaaeqabaWaaeGaeaaakeaaimaacqWFXeIraaa@3787@. A prefix *F *is removed if there are no symbols in *S *which match within the maximum gap. The algorithm stops once all *F *∈ ℱ
 MathType@MTEF@5@5@+=feaafiart1ev1aaatCvAUfKttLearuWrP9MDH5MBPbIqV92AaeXatLxBI9gBamrtHrhAL1wy0L2yHvtyaeHbnfgDOvwBHrxAJfwnaebbnrfifHhDYfgasaacH8akY=wiFfYdH8Gipec8Eeeu0xXdbba9frFj0=OqFfea0dXdd9vqai=hGuQ8kuc9pgc9s8qqaq=dirpe0xb9q8qiLsFr0=vr0=vr0dc8meaabaqaciaacaGaaeqabaWaaeGaeaaakeaaimaacqWFXeIraaa@3787@ are full positions or if the maximum span *L *has been reached.

**Figure 4:**
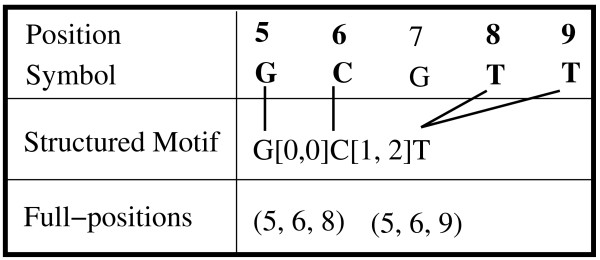
**Recomputed Full-position Recovery Example**. The figure shows how recomputed full-position recovery, using the structured example shown.

As an example, let's assume we want to recover the full position for the motif ℳ
 MathType@MTEF@5@5@+=feaafiart1ev1aaatCvAUfKttLearuWrP9MDH5MBPbIqV92AaeXatLxBI9gBamrtHrhAL1wy0L2yHvtyaeHbnfgDOvwBHrxAJfwnaebbnrfifHhDYfgasaacH8akY=wiFfYdH8Gipec8Eeeu0xXdbba9frFj0=OqFfea0dXdd9vqai=hGuQ8kuc9pgc9s8qqaq=dirpe0xb9q8qiLsFr0=vr0=vr0dc8meaabaqaciaacaGaaeqabaWaaeGaeaaakeaaimaacqWFZestaaa@3790@ = GC[1,2]T in our the sequence *S *from Table 4, starting from position *s *= 5. The recovery process is illustrated in Figure 4. Since the maximum span of ℳ
 MathType@MTEF@5@5@+=feaafiart1ev1aaatCvAUfKttLearuWrP9MDH5MBPbIqV92AaeXatLxBI9gBamrtHrhAL1wy0L2yHvtyaeHbnfgDOvwBHrxAJfwnaebbnrfifHhDYfgasaacH8akY=wiFfYdH8Gipec8Eeeu0xXdbba9frFj0=OqFfea0dXdd9vqai=hGuQ8kuc9pgc9s8qqaq=dirpe0xb9q8qiLsFr0=vr0=vr0dc8meaabaqaciaacaGaaeqabaWaaeGaeaaakeaaimaacqWFZestaaa@3790@ is *L *= 5 the figure shows positions 5 through 9 in *S*, which are the only valid positions where ℳ
 MathType@MTEF@5@5@+=feaafiart1ev1aaatCvAUfKttLearuWrP9MDH5MBPbIqV92AaeXatLxBI9gBamrtHrhAL1wy0L2yHvtyaeHbnfgDOvwBHrxAJfwnaebbnrfifHhDYfgasaacH8akY=wiFfYdH8Gipec8Eeeu0xXdbba9frFj0=OqFfea0dXdd9vqai=hGuQ8kuc9pgc9s8qqaq=dirpe0xb9q8qiLsFr0=vr0=vr0dc8meaabaqaciaacaGaaeqabaWaaeGaeaaakeaaimaacqWFZestaaa@3790@ may be found. Initially, ℱ
 MathType@MTEF@5@5@+=feaafiart1ev1aaatCvAUfKttLearuWrP9MDH5MBPbIqV92AaeXatLxBI9gBamrtHrhAL1wy0L2yHvtyaeHbnfgDOvwBHrxAJfwnaebbnrfifHhDYfgasaacH8akY=wiFfYdH8Gipec8Eeeu0xXdbba9frFj0=OqFfea0dXdd9vqai=hGuQ8kuc9pgc9s8qqaq=dirpe0xb9q8qiLsFr0=vr0=vr0dc8meaabaqaciaacaGaaeqabaWaaeGaeaaakeaaimaacqWFXeIraaa@3787@ = {(5)}, and we know that ℳ
 MathType@MTEF@5@5@+=feaafiart1ev1aaatCvAUfKttLearuWrP9MDH5MBPbIqV92AaeXatLxBI9gBamrtHrhAL1wy0L2yHvtyaeHbnfgDOvwBHrxAJfwnaebbnrfifHhDYfgasaacH8akY=wiFfYdH8Gipec8Eeeu0xXdbba9frFj0=OqFfea0dXdd9vqai=hGuQ8kuc9pgc9s8qqaq=dirpe0xb9q8qiLsFr0=vr0=vr0dc8meaabaqaciaacaGaaeqabaWaaeGaeaaakeaaimaacqWFZestaaa@3790@[1] = *S *[5]. Next *S *[6] = *C *= ℳ
 MathType@MTEF@5@5@+=feaafiart1ev1aaatCvAUfKttLearuWrP9MDH5MBPbIqV92AaeXatLxBI9gBamrtHrhAL1wy0L2yHvtyaeHbnfgDOvwBHrxAJfwnaebbnrfifHhDYfgasaacH8akY=wiFfYdH8Gipec8Eeeu0xXdbba9frFj0=OqFfea0dXdd9vqai=hGuQ8kuc9pgc9s8qqaq=dirpe0xb9q8qiLsFr0=vr0=vr0dc8meaabaqaciaacaGaaeqabaWaaeGaeaaakeaaimaacqWFZestaaa@3790@[2] match and are also adjacent in *S*, so we update ℱ
 MathType@MTEF@5@5@+=feaafiart1ev1aaatCvAUfKttLearuWrP9MDH5MBPbIqV92AaeXatLxBI9gBamrtHrhAL1wy0L2yHvtyaeHbnfgDOvwBHrxAJfwnaebbnrfifHhDYfgasaacH8akY=wiFfYdH8Gipec8Eeeu0xXdbba9frFj0=OqFfea0dXdd9vqai=hGuQ8kuc9pgc9s8qqaq=dirpe0xb9q8qiLsFr0=vr0=vr0dc8meaabaqaciaacaGaaeqabaWaaeGaeaaakeaaimaacqWFXeIraaa@3787@ = {(5, 6)}. We discard *S *[7] since it doesn't match ℳ
 MathType@MTEF@5@5@+=feaafiart1ev1aaatCvAUfKttLearuWrP9MDH5MBPbIqV92AaeXatLxBI9gBamrtHrhAL1wy0L2yHvtyaeHbnfgDOvwBHrxAJfwnaebbnrfifHhDYfgasaacH8akY=wiFfYdH8Gipec8Eeeu0xXdbba9frFj0=OqFfea0dXdd9vqai=hGuQ8kuc9pgc9s8qqaq=dirpe0xb9q8qiLsFr0=vr0=vr0dc8meaabaqaciaacaGaaeqabaWaaeGaeaaakeaaimaacqWFZestaaa@3790@[3]. However, both *S *[8] = *T *= ℳ
 MathType@MTEF@5@5@+=feaafiart1ev1aaatCvAUfKttLearuWrP9MDH5MBPbIqV92AaeXatLxBI9gBamrtHrhAL1wy0L2yHvtyaeHbnfgDOvwBHrxAJfwnaebbnrfifHhDYfgasaacH8akY=wiFfYdH8Gipec8Eeeu0xXdbba9frFj0=OqFfea0dXdd9vqai=hGuQ8kuc9pgc9s8qqaq=dirpe0xb9q8qiLsFr0=vr0=vr0dc8meaabaqaciaacaGaaeqabaWaaeGaeaaakeaaimaacqWFZestaaa@3790@[3] and *S *[9] = *T *= ℳ
 MathType@MTEF@5@5@+=feaafiart1ev1aaatCvAUfKttLearuWrP9MDH5MBPbIqV92AaeXatLxBI9gBamrtHrhAL1wy0L2yHvtyaeHbnfgDOvwBHrxAJfwnaebbnrfifHhDYfgasaacH8akY=wiFfYdH8Gipec8Eeeu0xXdbba9frFj0=OqFfea0dXdd9vqai=hGuQ8kuc9pgc9s8qqaq=dirpe0xb9q8qiLsFr0=vr0=vr0dc8meaabaqaciaacaGaaeqabaWaaeGaeaaakeaaimaacqWFZestaaa@3790@[3] are valid matches within the gap constraints. Thus we update ℱ
 MathType@MTEF@5@5@+=feaafiart1ev1aaatCvAUfKttLearuWrP9MDH5MBPbIqV92AaeXatLxBI9gBamrtHrhAL1wy0L2yHvtyaeHbnfgDOvwBHrxAJfwnaebbnrfifHhDYfgasaacH8akY=wiFfYdH8Gipec8Eeeu0xXdbba9frFj0=OqFfea0dXdd9vqai=hGuQ8kuc9pgc9s8qqaq=dirpe0xb9q8qiLsFr0=vr0=vr0dc8meaabaqaciaacaGaaeqabaWaaeGaeaaakeaaimaacqWFXeIraaa@3787@ = {(5, 6, 8), (5, 6, 9)}. At this point we have reached the maximum span, and also all *F *∈ ℱ
 MathType@MTEF@5@5@+=feaafiart1ev1aaatCvAUfKttLearuWrP9MDH5MBPbIqV92AaeXatLxBI9gBamrtHrhAL1wy0L2yHvtyaeHbnfgDOvwBHrxAJfwnaebbnrfifHhDYfgasaacH8akY=wiFfYdH8Gipec8Eeeu0xXdbba9frFj0=OqFfea0dXdd9vqai=hGuQ8kuc9pgc9s8qqaq=dirpe0xb9q8qiLsFr0=vr0=vr0dc8meaabaqaciaacaGaaeqabaWaaeGaeaaakeaaimaacqWFXeIraaa@3787@ are full positions, so we stop.

##### Indexed full position recovery

**Figure 5:**
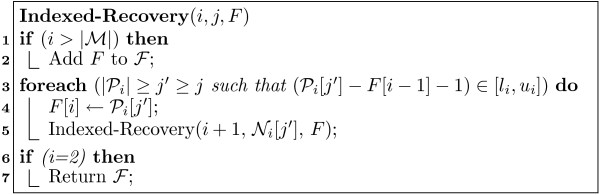
Indexed Full Position Recovery Algorithm.

Rather than recomputing the positions, we can "index" some information during the positional joins in order to facilitate full position recovery. For each suffix of ℳ
 MathType@MTEF@5@5@+=feaafiart1ev1aaatCvAUfKttLearuWrP9MDH5MBPbIqV92AaeXatLxBI9gBamrtHrhAL1wy0L2yHvtyaeHbnfgDOvwBHrxAJfwnaebbnrfifHhDYfgasaacH8akY=wiFfYdH8Gipec8Eeeu0xXdbba9frFj0=OqFfea0dXdd9vqai=hGuQ8kuc9pgc9s8qqaq=dirpe0xb9q8qiLsFr0=vr0=vr0dc8meaabaqaciaacaGaaeqabaWaaeGaeaaakeaaimaacqWFZestaaa@3790@ starting at position *i *with 1 ≤ *i *≤ |ℳ
 MathType@MTEF@5@5@+=feaafiart1ev1aaatCvAUfKttLearuWrP9MDH5MBPbIqV92AaeXatLxBI9gBamrtHrhAL1wy0L2yHvtyaeHbnfgDOvwBHrxAJfwnaebbnrfifHhDYfgasaacH8akY=wiFfYdH8Gipec8Eeeu0xXdbba9frFj0=OqFfea0dXdd9vqai=hGuQ8kuc9pgc9s8qqaq=dirpe0xb9q8qiLsFr0=vr0=vr0dc8meaabaqaciaacaGaaeqabaWaaeGaeaaakeaaimaacqWFZestaaa@3790@|, we keep its pos-list, P
 MathType@MTEF@5@5@+=feaafiart1ev1aaatCvAUfKttLearuWrP9MDH5MBPbIqV92AaeXatLxBI9gBamrtHrhAL1wy0L2yHvtyaeHbnfgDOvwBHrxAJfwnaebbnrfifHhDYfgasaacH8akY=wiFfYdH8Gipec8Eeeu0xXdbba9frFj0=OqFfea0dXdd9vqai=hGuQ8kuc9pgc9s8qqaq=dirpe0xb9q8qiLsFr0=vr0=vr0dc8meaabaqaciaacaGaaeqabaWaaeGaeaaakeaacqqGqbauaaa@3786@_*i*_, and an index list, N
 MathType@MTEF@5@5@+=feaafiart1ev1aaatCvAUfKttLearuWrP9MDH5MBPbIqV92AaeXatLxBI9gBamrtHrhAL1wy0L2yHvtyaeHbnfgDOvwBHrxAJfwnaebbnrfifHhDYfgasaacH8akY=wiFfYdH8Gipec8Eeeu0xXdbba9frFj0=OqFfea0dXdd9vqai=hGuQ8kuc9pgc9s8qqaq=dirpe0xb9q8qiLsFr0=vr0=vr0dc8meaabaqaciaacaGaaeqabaWaaeGaeaaakeaaimaacqWFneVtaaa@383B@_*i*_. For each entry, say P
 MathType@MTEF@5@5@+=feaafiart1ev1aaatCvAUfKttLearuWrP9MDH5MBPbIqV92AaeXatLxBI9gBamrtHrhAL1wy0L2yHvtyaeHbnfgDOvwBHrxAJfwnaebbnrfifHhDYfgasaacH8akY=wiFfYdH8Gipec8Eeeu0xXdbba9frFj0=OqFfea0dXdd9vqai=hGuQ8kuc9pgc9s8qqaq=dirpe0xb9q8qiLsFr0=vr0=vr0dc8meaabaqaciaacaGaaeqabaWaaeGaeaaakeaacqqGqbauaaa@3786@_*i *_[*j*], in the pos-list P
 MathType@MTEF@5@5@+=feaafiart1ev1aaatCvAUfKttLearuWrP9MDH5MBPbIqV92AaeXatLxBI9gBamrtHrhAL1wy0L2yHvtyaeHbnfgDOvwBHrxAJfwnaebbnrfifHhDYfgasaacH8akY=wiFfYdH8Gipec8Eeeu0xXdbba9frFj0=OqFfea0dXdd9vqai=hGuQ8kuc9pgc9s8qqaq=dirpe0xb9q8qiLsFr0=vr0=vr0dc8meaabaqaciaacaGaaeqabaWaaeGaeaaakeaacqqGqbauaaa@3786@_*i*_, the corresponding index entry N
 MathType@MTEF@5@5@+=feaafiart1ev1aaatCvAUfKttLearuWrP9MDH5MBPbIqV92AaeXatLxBI9gBamrtHrhAL1wy0L2yHvtyaeHbnfgDOvwBHrxAJfwnaebbnrfifHhDYfgasaacH8akY=wiFfYdH8Gipec8Eeeu0xXdbba9frFj0=OqFfea0dXdd9vqai=hGuQ8kuc9pgc9s8qqaq=dirpe0xb9q8qiLsFr0=vr0=vr0dc8meaabaqaciaacaGaaeqabaWaaeGaeaaakeaaimaacqWFneVtaaa@383B@_*i *_[*j*], points to the first entry, say *l*, in P
 MathType@MTEF@5@5@+=feaafiart1ev1aaatCvAUfKttLearuWrP9MDH5MBPbIqV92AaeXatLxBI9gBamrtHrhAL1wy0L2yHvtyaeHbnfgDOvwBHrxAJfwnaebbnrfifHhDYfgasaacH8akY=wiFfYdH8Gipec8Eeeu0xXdbba9frFj0=OqFfea0dXdd9vqai=hGuQ8kuc9pgc9s8qqaq=dirpe0xb9q8qiLsFr0=vr0=vr0dc8meaabaqaciaacaGaaeqabaWaaeGaeaaakeaacqqGqbauaaa@3786@_*i*+1 _that satisfies the gap range with respect to P
 MathType@MTEF@5@5@+=feaafiart1ev1aaatCvAUfKttLearuWrP9MDH5MBPbIqV92AaeXatLxBI9gBamrtHrhAL1wy0L2yHvtyaeHbnfgDOvwBHrxAJfwnaebbnrfifHhDYfgasaacH8akY=wiFfYdH8Gipec8Eeeu0xXdbba9frFj0=OqFfea0dXdd9vqai=hGuQ8kuc9pgc9s8qqaq=dirpe0xb9q8qiLsFr0=vr0=vr0dc8meaabaqaciaacaGaaeqabaWaaeGaeaaakeaacqqGqbauaaa@3786@_*i *_[*j*], i.e., P
 MathType@MTEF@5@5@+=feaafiart1ev1aaatCvAUfKttLearuWrP9MDH5MBPbIqV92AaeXatLxBI9gBamrtHrhAL1wy0L2yHvtyaeHbnfgDOvwBHrxAJfwnaebbnrfifHhDYfgasaacH8akY=wiFfYdH8Gipec8Eeeu0xXdbba9frFj0=OqFfea0dXdd9vqai=hGuQ8kuc9pgc9s8qqaq=dirpe0xb9q8qiLsFr0=vr0=vr0dc8meaabaqaciaacaGaaeqabaWaaeGaeaaakeaacqqGqbauaaa@3786@_*i*+1 _[*l*] - P
 MathType@MTEF@5@5@+=feaafiart1ev1aaatCvAUfKttLearuWrP9MDH5MBPbIqV92AaeXatLxBI9gBamrtHrhAL1wy0L2yHvtyaeHbnfgDOvwBHrxAJfwnaebbnrfifHhDYfgasaacH8akY=wiFfYdH8Gipec8Eeeu0xXdbba9frFj0=OqFfea0dXdd9vqai=hGuQ8kuc9pgc9s8qqaq=dirpe0xb9q8qiLsFr0=vr0=vr0dc8meaabaqaciaacaGaaeqabaWaaeGaeaaakeaacqqGqbauaaa@3786@_*i *_[*j*] - 1 ∈ [*l*_*i*_, *u*_*i*_]. Note that N|ℳ|
 MathType@MTEF@5@5@+=feaafiart1ev1aaatCvAUfKttLearuWrP9MDH5MBPbIqV92AaeXatLxBI9gBamrtHrhAL1wy0L2yHvtyaeHbnfgDOvwBHrxAJfwnaebbnrfifHhDYfgasaacH8akY=wiFfYdH8Gipec8Eeeu0xXdbba9frFj0=OqFfea0dXdd9vqai=hGuQ8kuc9pgc9s8qqaq=dirpe0xb9q8qiLsFr0=vr0=vr0dc8meaabaqaciaacaGaaeqabaWaaeGaeaaakeaaimaacqWFneVtdaWgaaWcbaGaeiiFaWNae83mH0KaeiiFaWhabeaaaaa@3C8D@ is never used. Also note that P
 MathType@MTEF@5@5@+=feaafiart1ev1aaatCvAUfKttLearuWrP9MDH5MBPbIqV92AaeXatLxBI9gBamrtHrhAL1wy0L2yHvtyaeHbnfgDOvwBHrxAJfwnaebbnrfifHhDYfgasaacH8akY=wiFfYdH8Gipec8Eeeu0xXdbba9frFj0=OqFfea0dXdd9vqai=hGuQ8kuc9pgc9s8qqaq=dirpe0xb9q8qiLsFr0=vr0=vr0dc8meaabaqaciaacaGaaeqabaWaaeGaeaaakeaacqqGqbauaaa@3786@(ℳ
 MathType@MTEF@5@5@+=feaafiart1ev1aaatCvAUfKttLearuWrP9MDH5MBPbIqV92AaeXatLxBI9gBamrtHrhAL1wy0L2yHvtyaeHbnfgDOvwBHrxAJfwnaebbnrfifHhDYfgasaacH8akY=wiFfYdH8Gipec8Eeeu0xXdbba9frFj0=OqFfea0dXdd9vqai=hGuQ8kuc9pgc9s8qqaq=dirpe0xb9q8qiLsFr0=vr0=vr0dc8meaabaqaciaacaGaaeqabaWaaeGaeaaakeaaimaacqWFZestaaa@3790@) = P
 MathType@MTEF@5@5@+=feaafiart1ev1aaatCvAUfKttLearuWrP9MDH5MBPbIqV92AaeXatLxBI9gBamrtHrhAL1wy0L2yHvtyaeHbnfgDOvwBHrxAJfwnaebbnrfifHhDYfgasaacH8akY=wiFfYdH8Gipec8Eeeu0xXdbba9frFj0=OqFfea0dXdd9vqai=hGuQ8kuc9pgc9s8qqaq=dirpe0xb9q8qiLsFr0=vr0=vr0dc8meaabaqaciaacaGaaeqabaWaaeGaeaaakeaacqqGqbauaaa@3786@_1_. Let *s *be a start position for the structured motif in sequence *S*, and let *s *be the *j*_*s*_-th entry in P
 MathType@MTEF@5@5@+=feaafiart1ev1aaatCvAUfKttLearuWrP9MDH5MBPbIqV92AaeXatLxBI9gBamrtHrhAL1wy0L2yHvtyaeHbnfgDOvwBHrxAJfwnaebbnrfifHhDYfgasaacH8akY=wiFfYdH8Gipec8Eeeu0xXdbba9frFj0=OqFfea0dXdd9vqai=hGuQ8kuc9pgc9s8qqaq=dirpe0xb9q8qiLsFr0=vr0=vr0dc8meaabaqaciaacaGaaeqabaWaaeGaeaaakeaacqqGqbauaaa@3786@_1_, i.e., *s *= P
 MathType@MTEF@5@5@+=feaafiart1ev1aaatCvAUfKttLearuWrP9MDH5MBPbIqV92AaeXatLxBI9gBamrtHrhAL1wy0L2yHvtyaeHbnfgDOvwBHrxAJfwnaebbnrfifHhDYfgasaacH8akY=wiFfYdH8Gipec8Eeeu0xXdbba9frFj0=OqFfea0dXdd9vqai=hGuQ8kuc9pgc9s8qqaq=dirpe0xb9q8qiLsFr0=vr0=vr0dc8meaabaqaciaacaGaaeqabaWaaeGaeaaakeaacqqGqbauaaa@3786@_1_[*j*_*s*_]. Also let *F *store a full position starting from *s*, and let ℱ
 MathType@MTEF@5@5@+=feaafiart1ev1aaatCvAUfKttLearuWrP9MDH5MBPbIqV92AaeXatLxBI9gBamrtHrhAL1wy0L2yHvtyaeHbnfgDOvwBHrxAJfwnaebbnrfifHhDYfgasaacH8akY=wiFfYdH8Gipec8Eeeu0xXdbba9frFj0=OqFfea0dXdd9vqai=hGuQ8kuc9pgc9s8qqaq=dirpe0xb9q8qiLsFr0=vr0=vr0dc8meaabaqaciaacaGaaeqabaWaaeGaeaaakeaaimaacqWFXeIraaa@3787@ store the set of all full positions. Figure 5 shows the pseudo-code for recovering full positions starting from *s*. This recursive algorithm has two parameters: *i *denotes a (suffix) position in ℳ
 MathType@MTEF@5@5@+=feaafiart1ev1aaatCvAUfKttLearuWrP9MDH5MBPbIqV92AaeXatLxBI9gBamrtHrhAL1wy0L2yHvtyaeHbnfgDOvwBHrxAJfwnaebbnrfifHhDYfgasaacH8akY=wiFfYdH8Gipec8Eeeu0xXdbba9frFj0=OqFfea0dXdd9vqai=hGuQ8kuc9pgc9s8qqaq=dirpe0xb9q8qiLsFr0=vr0=vr0dc8meaabaqaciaacaGaaeqabaWaaeGaeaaakeaaimaacqWFZestaaa@3790@, and *j *gives the *j*-th entry in P
 MathType@MTEF@5@5@+=feaafiart1ev1aaatCvAUfKttLearuWrP9MDH5MBPbIqV92AaeXatLxBI9gBamrtHrhAL1wy0L2yHvtyaeHbnfgDOvwBHrxAJfwnaebbnrfifHhDYfgasaacH8akY=wiFfYdH8Gipec8Eeeu0xXdbba9frFj0=OqFfea0dXdd9vqai=hGuQ8kuc9pgc9s8qqaq=dirpe0xb9q8qiLsFr0=vr0=vr0dc8meaabaqaciaacaGaaeqabaWaaeGaeaaakeaacqqGqbauaaa@3786@_*i*_. The algorithm is initially called with ℱ
 MathType@MTEF@5@5@+=feaafiart1ev1aaatCvAUfKttLearuWrP9MDH5MBPbIqV92AaeXatLxBI9gBamrtHrhAL1wy0L2yHvtyaeHbnfgDOvwBHrxAJfwnaebbnrfifHhDYfgasaacH8akY=wiFfYdH8Gipec8Eeeu0xXdbba9frFj0=OqFfea0dXdd9vqai=hGuQ8kuc9pgc9s8qqaq=dirpe0xb9q8qiLsFr0=vr0=vr0dc8meaabaqaciaacaGaaeqabaWaaeGaeaaakeaaimaacqWFXeIraaa@3787@ = {*F *= {*s*}}, *i *= 2 and with *j *= N
 MathType@MTEF@5@5@+=feaafiart1ev1aaatCvAUfKttLearuWrP9MDH5MBPbIqV92AaeXatLxBI9gBamrtHrhAL1wy0L2yHvtyaeHbnfgDOvwBHrxAJfwnaebbnrfifHhDYfgasaacH8akY=wiFfYdH8Gipec8Eeeu0xXdbba9frFj0=OqFfea0dXdd9vqai=hGuQ8kuc9pgc9s8qqaq=dirpe0xb9q8qiLsFr0=vr0=vr0dc8meaabaqaciaacaGaaeqabaWaaeGaeaaakeaaimaacqWFneVtaaa@383B@_1_[*j*_*s*_]. Since we have P
 MathType@MTEF@5@5@+=feaafiart1ev1aaatCvAUfKttLearuWrP9MDH5MBPbIqV92AaeXatLxBI9gBamrtHrhAL1wy0L2yHvtyaeHbnfgDOvwBHrxAJfwnaebbnrfifHhDYfgasaacH8akY=wiFfYdH8Gipec8Eeeu0xXdbba9frFj0=OqFfea0dXdd9vqai=hGuQ8kuc9pgc9s8qqaq=dirpe0xb9q8qiLsFr0=vr0=vr0dc8meaabaqaciaacaGaaeqabaWaaeGaeaaakeaacqqGqbauaaa@3786@_2_[*j*] - *F *[1] - 1 ∈ [*l*_1_, *u*_1_] we set *F *[2] = P
 MathType@MTEF@5@5@+=feaafiart1ev1aaatCvAUfKttLearuWrP9MDH5MBPbIqV92AaeXatLxBI9gBamrtHrhAL1wy0L2yHvtyaeHbnfgDOvwBHrxAJfwnaebbnrfifHhDYfgasaacH8akY=wiFfYdH8Gipec8Eeeu0xXdbba9frFj0=OqFfea0dXdd9vqai=hGuQ8kuc9pgc9s8qqaq=dirpe0xb9q8qiLsFr0=vr0=vr0dc8meaabaqaciaacaGaaeqabaWaaeGaeaaakeaacqqGqbauaaa@3786@_2_[*j*]. In the next call we can follow the index N
 MathType@MTEF@5@5@+=feaafiart1ev1aaatCvAUfKttLearuWrP9MDH5MBPbIqV92AaeXatLxBI9gBamrtHrhAL1wy0L2yHvtyaeHbnfgDOvwBHrxAJfwnaebbnrfifHhDYfgasaacH8akY=wiFfYdH8Gipec8Eeeu0xXdbba9frFj0=OqFfea0dXdd9vqai=hGuQ8kuc9pgc9s8qqaq=dirpe0xb9q8qiLsFr0=vr0=vr0dc8meaabaqaciaacaGaaeqabaWaaeGaeaaakeaaimaacqWFneVtaaa@383B@_2_[*j*] to get the next position in *F*, namely *F *[3]. Thus in each call we keep following the indices from one pos-list to the next and finally we can get a full position starting from *s *when we reach the last pos-list, ${\text{P}}_{\left|\text{M}\right|}$. Furthermore, at each suffix position *i*, since *j *only marks the first position in P
 MathType@MTEF@5@5@+=feaafiart1ev1aaatCvAUfKttLearuWrP9MDH5MBPbIqV92AaeXatLxBI9gBamrtHrhAL1wy0L2yHvtyaeHbnfgDOvwBHrxAJfwnaebbnrfifHhDYfgasaacH8akY=wiFfYdH8Gipec8Eeeu0xXdbba9frFj0=OqFfea0dXdd9vqai=hGuQ8kuc9pgc9s8qqaq=dirpe0xb9q8qiLsFr0=vr0=vr0dc8meaabaqaciaacaGaaeqabaWaaeGaeaaakeaacqqGqbauaaa@3786@_*i*+1 _that satisfies the gap constraints, we also need to consider all the subsequent positions *j' *> *j *that may satisfy the corresponding gap range.

**Figure 6:**
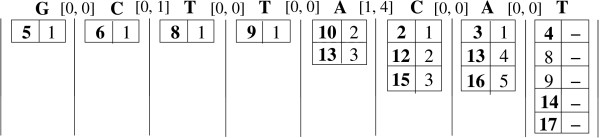
**Indexed Full-position Recovery Example**. The figure shows how indexed full-position recovery, using the (expanded) motif from Table 4.

Consider the example shown in Figure 6 to recover the full positions for our example motif from Table 4. Under each symbol we show two columns. The left column corresponds to the intermediate pos-lists P
 MathType@MTEF@5@5@+=feaafiart1ev1aaatCvAUfKttLearuWrP9MDH5MBPbIqV92AaeXatLxBI9gBamrtHrhAL1wy0L2yHvtyaeHbnfgDOvwBHrxAJfwnaebbnrfifHhDYfgasaacH8akY=wiFfYdH8Gipec8Eeeu0xXdbba9frFj0=OqFfea0dXdd9vqai=hGuQ8kuc9pgc9s8qqaq=dirpe0xb9q8qiLsFr0=vr0=vr0dc8meaabaqaciaacaGaaeqabaWaaeGaeaaakeaacqqGqbauaaa@3786@_*i *_(compare to Figure 2), whereas the right column stores the indices N
 MathType@MTEF@5@5@+=feaafiart1ev1aaatCvAUfKttLearuWrP9MDH5MBPbIqV92AaeXatLxBI9gBamrtHrhAL1wy0L2yHvtyaeHbnfgDOvwBHrxAJfwnaebbnrfifHhDYfgasaacH8akY=wiFfYdH8Gipec8Eeeu0xXdbba9frFj0=OqFfea0dXdd9vqai=hGuQ8kuc9pgc9s8qqaq=dirpe0xb9q8qiLsFr0=vr0=vr0dc8meaabaqaciaacaGaaeqabaWaaeGaeaaakeaaimaacqWFneVtaaa@383B@_*i *_into the pos-list P
 MathType@MTEF@5@5@+=feaafiart1ev1aaatCvAUfKttLearuWrP9MDH5MBPbIqV92AaeXatLxBI9gBamrtHrhAL1wy0L2yHvtyaeHbnfgDOvwBHrxAJfwnaebbnrfifHhDYfgasaacH8akY=wiFfYdH8Gipec8Eeeu0xXdbba9frFj0=OqFfea0dXdd9vqai=hGuQ8kuc9pgc9s8qqaq=dirpe0xb9q8qiLsFr0=vr0=vr0dc8meaabaqaciaacaGaaeqabaWaaeGaeaaakeaacqqGqbauaaa@3786@_*i*+1_. For example, P
 MathType@MTEF@5@5@+=feaafiart1ev1aaatCvAUfKttLearuWrP9MDH5MBPbIqV92AaeXatLxBI9gBamrtHrhAL1wy0L2yHvtyaeHbnfgDOvwBHrxAJfwnaebbnrfifHhDYfgasaacH8akY=wiFfYdH8Gipec8Eeeu0xXdbba9frFj0=OqFfea0dXdd9vqai=hGuQ8kuc9pgc9s8qqaq=dirpe0xb9q8qiLsFr0=vr0=vr0dc8meaabaqaciaacaGaaeqabaWaaeGaeaaakeaacqqGqbauaaa@3786@(*A*[0,0]*T*) = P
 MathType@MTEF@5@5@+=feaafiart1ev1aaatCvAUfKttLearuWrP9MDH5MBPbIqV92AaeXatLxBI9gBamrtHrhAL1wy0L2yHvtyaeHbnfgDOvwBHrxAJfwnaebbnrfifHhDYfgasaacH8akY=wiFfYdH8Gipec8Eeeu0xXdbba9frFj0=OqFfea0dXdd9vqai=hGuQ8kuc9pgc9s8qqaq=dirpe0xb9q8qiLsFr0=vr0=vr0dc8meaabaqaciaacaGaaeqabaWaaeGaeaaakeaacqqGqbauaaa@3786@_7 _= {3, 13, 16}, and N
 MathType@MTEF@5@5@+=feaafiart1ev1aaatCvAUfKttLearuWrP9MDH5MBPbIqV92AaeXatLxBI9gBamrtHrhAL1wy0L2yHvtyaeHbnfgDOvwBHrxAJfwnaebbnrfifHhDYfgasaacH8akY=wiFfYdH8Gipec8Eeeu0xXdbba9frFj0=OqFfea0dXdd9vqai=hGuQ8kuc9pgc9s8qqaq=dirpe0xb9q8qiLsFr0=vr0=vr0dc8meaabaqaciaacaGaaeqabaWaaeGaeaaakeaaimaacqWFneVtaaa@383B@_7 _= {1, 4, 5}. For example, for entry P
 MathType@MTEF@5@5@+=feaafiart1ev1aaatCvAUfKttLearuWrP9MDH5MBPbIqV92AaeXatLxBI9gBamrtHrhAL1wy0L2yHvtyaeHbnfgDOvwBHrxAJfwnaebbnrfifHhDYfgasaacH8akY=wiFfYdH8Gipec8Eeeu0xXdbba9frFj0=OqFfea0dXdd9vqai=hGuQ8kuc9pgc9s8qqaq=dirpe0xb9q8qiLsFr0=vr0=vr0dc8meaabaqaciaacaGaaeqabaWaaeGaeaaakeaacqqGqbauaaa@3786@_7_[2] = 13, we have N
 MathType@MTEF@5@5@+=feaafiart1ev1aaatCvAUfKttLearuWrP9MDH5MBPbIqV92AaeXatLxBI9gBamrtHrhAL1wy0L2yHvtyaeHbnfgDOvwBHrxAJfwnaebbnrfifHhDYfgasaacH8akY=wiFfYdH8Gipec8Eeeu0xXdbba9frFj0=OqFfea0dXdd9vqai=hGuQ8kuc9pgc9s8qqaq=dirpe0xb9q8qiLsFr0=vr0=vr0dc8meaabaqaciaacaGaaeqabaWaaeGaeaaakeaaimaacqWFneVtaaa@383B@_7_[2] = 4, which means that the first position in P
 MathType@MTEF@5@5@+=feaafiart1ev1aaatCvAUfKttLearuWrP9MDH5MBPbIqV92AaeXatLxBI9gBamrtHrhAL1wy0L2yHvtyaeHbnfgDOvwBHrxAJfwnaebbnrfifHhDYfgasaacH8akY=wiFfYdH8Gipec8Eeeu0xXdbba9frFj0=OqFfea0dXdd9vqai=hGuQ8kuc9pgc9s8qqaq=dirpe0xb9q8qiLsFr0=vr0=vr0dc8meaabaqaciaacaGaaeqabaWaaeGaeaaakeaacqqGqbauaaa@3786@_8 _= P
 MathType@MTEF@5@5@+=feaafiart1ev1aaatCvAUfKttLearuWrP9MDH5MBPbIqV92AaeXatLxBI9gBamrtHrhAL1wy0L2yHvtyaeHbnfgDOvwBHrxAJfwnaebbnrfifHhDYfgasaacH8akY=wiFfYdH8Gipec8Eeeu0xXdbba9frFj0=OqFfea0dXdd9vqai=hGuQ8kuc9pgc9s8qqaq=dirpe0xb9q8qiLsFr0=vr0=vr0dc8meaabaqaciaacaGaaeqabaWaaeGaeaaakeaacqqGqbauaaa@3786@(*T*) that satisfies the gap range [0,0] is 14, which occurs at index 4, i.e., P
 MathType@MTEF@5@5@+=feaafiart1ev1aaatCvAUfKttLearuWrP9MDH5MBPbIqV92AaeXatLxBI9gBamrtHrhAL1wy0L2yHvtyaeHbnfgDOvwBHrxAJfwnaebbnrfifHhDYfgasaacH8akY=wiFfYdH8Gipec8Eeeu0xXdbba9frFj0=OqFfea0dXdd9vqai=hGuQ8kuc9pgc9s8qqaq=dirpe0xb9q8qiLsFr0=vr0=vr0dc8meaabaqaciaacaGaaeqabaWaaeGaeaaakeaacqqGqbauaaa@3786@_8_[4] - P
 MathType@MTEF@5@5@+=feaafiart1ev1aaatCvAUfKttLearuWrP9MDH5MBPbIqV92AaeXatLxBI9gBamrtHrhAL1wy0L2yHvtyaeHbnfgDOvwBHrxAJfwnaebbnrfifHhDYfgasaacH8akY=wiFfYdH8Gipec8Eeeu0xXdbba9frFj0=OqFfea0dXdd9vqai=hGuQ8kuc9pgc9s8qqaq=dirpe0xb9q8qiLsFr0=vr0=vr0dc8meaabaqaciaacaGaaeqabaWaaeGaeaaakeaacqqGqbauaaa@3786@_7_[2] - 1 = 14 - 13 - 1 = 0 ∈ [0,0]. To recover the full position for our example motif, from start position 5, we follow index 1 to get position 6 in the next pos-list, to obtain ℱ
 MathType@MTEF@5@5@+=feaafiart1ev1aaatCvAUfKttLearuWrP9MDH5MBPbIqV92AaeXatLxBI9gBamrtHrhAL1wy0L2yHvtyaeHbnfgDOvwBHrxAJfwnaebbnrfifHhDYfgasaacH8akY=wiFfYdH8Gipec8Eeeu0xXdbba9frFj0=OqFfea0dXdd9vqai=hGuQ8kuc9pgc9s8qqaq=dirpe0xb9q8qiLsFr0=vr0=vr0dc8meaabaqaciaacaGaaeqabaWaaeGaeaaakeaaimaacqWFXeIraaa@3787@ = {(5, 6)}. Then we keep following the indices and get ℱ
 MathType@MTEF@5@5@+=feaafiart1ev1aaatCvAUfKttLearuWrP9MDH5MBPbIqV92AaeXatLxBI9gBamrtHrhAL1wy0L2yHvtyaeHbnfgDOvwBHrxAJfwnaebbnrfifHhDYfgasaacH8akY=wiFfYdH8Gipec8Eeeu0xXdbba9frFj0=OqFfea0dXdd9vqai=hGuQ8kuc9pgc9s8qqaq=dirpe0xb9q8qiLsFr0=vr0=vr0dc8meaabaqaciaacaGaaeqabaWaaeGaeaaakeaaimaacqWFXeIraaa@3787@ = {(5, 6, 8, 9)}. In the next step, we follow to position 10 (at index 1); a quick check for the gap range [0,0] discards position 13. We now have ℱ
 MathType@MTEF@5@5@+=feaafiart1ev1aaatCvAUfKttLearuWrP9MDH5MBPbIqV92AaeXatLxBI9gBamrtHrhAL1wy0L2yHvtyaeHbnfgDOvwBHrxAJfwnaebbnrfifHhDYfgasaacH8akY=wiFfYdH8Gipec8Eeeu0xXdbba9frFj0=OqFfea0dXdd9vqai=hGuQ8kuc9pgc9s8qqaq=dirpe0xb9q8qiLsFr0=vr0=vr0dc8meaabaqaciaacaGaaeqabaWaaeGaeaaakeaaimaacqWFXeIraaa@3787@ = {(5, 6, 8, 9, 10)}. In the next step we immediately jump to position 12 (at index 2). However, both 12 and 15 are within the gap range [1,4]. From 12, we will eventually get *F *= (5, 6, 8, 9, 10, 12, 13, 14), whereas from 15, we will eventually get *F *= (5, 6, 8, 9, 10, 15, 16, 17), as the two possible full-positions.

### Sequence segmentation

The SMOTIF approach as described above works well for searching a motif in a relatively short sequence. For a very long sequence *S *(e.g., searching for *(LTR) retrotransposons *in an entire chromosome) the pos-lists can get very long in the initial stages, consuming a lot of memory. SMOTIF handles a long sequence by splitting it into several segments and searches each segment separately for the structured motif. That is, the sequence *S *is split into *p *equal partitions (except for the last one). Handling each smaller segment *S*_*i *_(*i *∈ [l, *p*]) instead of the original *S *can save a lot of space and also reduces the total search time. After segmentation, to avoid missing any occurrence, we require that each partition *S*_*i*_, with *i *∈ [l, *p *- 1], include the first *L *- 1 symbols from partition *S*_*i*+1_. Finally, to avoid duplicate occurrences, we discard all occurrences with a start position in the overlap region, since it would be reported when we process segment *S*_*i*+1_. For example, let *S *be the sequence in Table 6, and let the structured motif be ℳ
 MathType@MTEF@5@5@+=feaafiart1ev1aaatCvAUfKttLearuWrP9MDH5MBPbIqV92AaeXatLxBI9gBamrtHrhAL1wy0L2yHvtyaeHbnfgDOvwBHrxAJfwnaebbnrfifHhDYfgasaacH8akY=wiFfYdH8Gipec8Eeeu0xXdbba9frFj0=OqFfea0dXdd9vqai=hGuQ8kuc9pgc9s8qqaq=dirpe0xb9q8qiLsFr0=vr0=vr0dc8meaabaqaciaacaGaaeqabaWaaeGaeaaakeaaimaacqWFZestaaa@3790@ = GC[1,2]T with maximum span *L *= 5. If *p *= 3, then we would have three segments of length 6 each. After adding the overlap region of *L *- 1 = 4 positions at the end of each segment, we obtain the final three segments shown in Table 6. Two start positions of ℳ
 MathType@MTEF@5@5@+=feaafiart1ev1aaatCvAUfKttLearuWrP9MDH5MBPbIqV92AaeXatLxBI9gBamrtHrhAL1wy0L2yHvtyaeHbnfgDOvwBHrxAJfwnaebbnrfifHhDYfgasaacH8akY=wiFfYdH8Gipec8Eeeu0xXdbba9frFj0=OqFfea0dXdd9vqai=hGuQ8kuc9pgc9s8qqaq=dirpe0xb9q8qiLsFr0=vr0=vr0dc8meaabaqaciaacaGaaeqabaWaaeGaeaaakeaaimaacqWFZestaaa@3790@ would be found in *S*_1 _(namely 1 and 5), and one in *S*_2 _(namely 11). Note that start positions 5 and 11 would have been missed if we had no overlap.

So far we have assumed that we are searching for the structured motif in a single sequence. SMOTIF can easily handle a collection S
 MathType@MTEF@5@5@+=feaafiart1ev1aaatCvAUfKttLearuWrP9MDH5MBPbIqV92AaeXatLxBI9gBamrtHrhAL1wy0L2yHvtyaeHbnfgDOvwBHrxAJfwnaebbnrfifHhDYfgasaacH8akY=wiFfYdH8Gipec8Eeeu0xXdbba9frFj0=OqFfea0dXdd9vqai=hGuQ8kuc9pgc9s8qqaq=dirpe0xb9q8qiLsFr0=vr0=vr0dc8meaabaqaciaacaGaaeqabaWaaeGaeaaakeaaimaacqWFse=uaaa@3845@ of sequences. We simply search each sequence separately using segmentation when necessary.

**Table 6:** Segmentation into *p *= 3

*S *		G	C	A	T	G	C	G	T	T	A	G	C	A	T	C	A	T	C
*S*_1 _		G	C	A	T	G	C	G	T	T	A								
*S*_2 _								G	T	T	A	G	C	A	T	C	A		
*S*_3 _														A	T	C	A	T	C

### Missing components

In some applications a partial match of the structured motif might still be of interest. SMOTIF allows up to *q *simple motif components to be missing during the search. Let ℳ
 MathType@MTEF@5@5@+=feaafiart1ev1aaatCvAUfKttLearuWrP9MDH5MBPbIqV92AaeXatLxBI9gBamrtHrhAL1wy0L2yHvtyaeHbnfgDOvwBHrxAJfwnaebbnrfifHhDYfgasaacH8akY=wiFfYdH8Gipec8Eeeu0xXdbba9frFj0=OqFfea0dXdd9vqai=hGuQ8kuc9pgc9s8qqaq=dirpe0xb9q8qiLsFr0=vr0=vr0dc8meaabaqaciaacaGaaeqabaWaaeGaeaaakeaaimaacqWFZestaaa@3790@ be a structured motif with *k *components. SMOTIF first enumerates all possible sub-motifs having *k' *components, where *k' *∈ [*k *- *q*, *k*]. Next, the gap ranges are adjusted in each sub-motif to account for skipping over the missing components. The new gap range, [*l*_*i,j*_, *u*_*i,j*_], between components *M*_*i *_and *M*_*j *_(with 1 ≤ *i *<*j *≤ *k*) in a sub-motif, is calculated as follows: li,j=∑n=1j−1ln
 MathType@MTEF@5@5@+=feaafiart1ev1aaatCvAUfKttLearuWrP9MDH5MBPbIqV92AaeXatLxBI9gBaebbnrfifHhDYfgasaacH8akY=wiFfYdH8Gipec8Eeeu0xXdbba9frFj0=OqFfea0dXdd9vqai=hGuQ8kuc9pgc9s8qqaq=dirpe0xb9q8qiLsFr0=vr0=vr0dc8meaabaqaciaacaGaaeqabaqabeGadaaakeaacqWGSbaBdaWgaaWcbaGaemyAaKMaeiilaWIaemOAaOgabeaakiabg2da9maaqadabaGaemiBaW2aaSbaaSqaaiabd6gaUbqabaaabaGaemOBa4Maeyypa0JaeGymaedabaGaemOAaOMaeyOeI0IaeGymaedaniabggHiLdaaaa@3E5F@, and ui,j=ui+∑n=i+1j−1(un+|Mn|)
 MathType@MTEF@5@5@+=feaafiart1ev1aaatCvAUfKttLearuWrP9MDH5MBPbIqV92AaeXatLxBI9gBaebbnrfifHhDYfgasaacH8akY=wiFfYdH8Gipec8Eeeu0xXdbba9frFj0=OqFfea0dXdd9vqai=hGuQ8kuc9pgc9s8qqaq=dirpe0xb9q8qiLsFr0=vr0=vr0dc8meaabaqaciaacaGaaeqabaqabeGadaaakeaacqWG1bqDdaWgaaWcbaGaemyAaKMaeiilaWIaemOAaOgabeaakiabg2da9iabdwha1naaBaaaleaacqWGPbqAaeqaaOGaey4kaSYaaabmaeaacqGGOaakcqWG1bqDdaWgaaWcbaGaemOBa4gabeaakiabgUcaRiabcYha8jabd2eannaaBaaaleaacqWGUbGBaeqaaOGaeiiFaWNaeiykaKcaleaacqWGUbGBcqGH9aqpcqWGPbqAcqGHRaWkcqaIXaqmaeaacqWGQbGAcqGHsislcqaIXaqma0GaeyyeIuoaaaa@4D0D@.

For example, if we allow one (*q *= 1) missing component for our structured motif in Table 4, the set of sub-motifs that need to be searched for are: GC[0,1]TTA[1,4]CAT, GC[1,8]CAT, GC[0,1]TTA and TTA[1,4]CAT. Note that it is straightforward to incorporate other approaches to compute new ranges into SMOTIF since it would only change the gap constraints. For example, *l*_*i,j *_= min_*n *∈ [*i,j*-1] _{*l*_*n*_} and *u*_*i,j *_= max_*n *∈ [*i,j*-1] _{*u*_*n*_} is another possible way to compute the adjusted gap ranges.

Instead of searching each sub-motif separately, we do an optimized search. We reuse the partial pos-lists created when using a depth first search to enumerate and search the sub-motifs. The idea is to re-use the pos-lists created for common suffixes when enumerating their sub-motif extensions.

### Two-step approach for structured pattern search

So far we have described the direct method used by SMOTIF to search for the structured motif by positional joins over the symbols. In fact, SMOTIF, can also follow a two-step approach like in Anrep [4] and SMARTFINDER [6]. In the first step, given S
 MathType@MTEF@5@5@+=feaafiart1ev1aaatCvAUfKttLearuWrP9MDH5MBPbIqV92AaeXatLxBI9gBamrtHrhAL1wy0L2yHvtyaeHbnfgDOvwBHrxAJfwnaebbnrfifHhDYfgasaacH8akY=wiFfYdH8Gipec8Eeeu0xXdbba9frFj0=OqFfea0dXdd9vqai=hGuQ8kuc9pgc9s8qqaq=dirpe0xb9q8qiLsFr0=vr0=vr0dc8meaabaqaciaacaGaaeqabaWaaeGaeaaakeaaimaacqWFse=uaaa@3845@ and ℳ
 MathType@MTEF@5@5@+=feaafiart1ev1aaatCvAUfKttLearuWrP9MDH5MBPbIqV92AaeXatLxBI9gBamrtHrhAL1wy0L2yHvtyaeHbnfgDOvwBHrxAJfwnaebbnrfifHhDYfgasaacH8akY=wiFfYdH8Gipec8Eeeu0xXdbba9frFj0=OqFfea0dXdd9vqai=hGuQ8kuc9pgc9s8qqaq=dirpe0xb9q8qiLsFr0=vr0=vr0dc8meaabaqaciaacaGaaeqabaWaaeGaeaaakeaaimaacqWFZestaaa@3790@, we search S
 MathType@MTEF@5@5@+=feaafiart1ev1aaatCvAUfKttLearuWrP9MDH5MBPbIqV92AaeXatLxBI9gBamrtHrhAL1wy0L2yHvtyaeHbnfgDOvwBHrxAJfwnaebbnrfifHhDYfgasaacH8akY=wiFfYdH8Gipec8Eeeu0xXdbba9frFj0=OqFfea0dXdd9vqai=hGuQ8kuc9pgc9s8qqaq=dirpe0xb9q8qiLsFr0=vr0=vr0dc8meaabaqaciaacaGaaeqabaWaaeGaeaaakeaaimaacqWFse=uaaa@3845@ for each simple motif in ℳ
 MathType@MTEF@5@5@+=feaafiart1ev1aaatCvAUfKttLearuWrP9MDH5MBPbIqV92AaeXatLxBI9gBamrtHrhAL1wy0L2yHvtyaeHbnfgDOvwBHrxAJfwnaebbnrfifHhDYfgasaacH8akY=wiFfYdH8Gipec8Eeeu0xXdbba9frFj0=OqFfea0dXdd9vqai=hGuQ8kuc9pgc9s8qqaq=dirpe0xb9q8qiLsFr0=vr0=vr0dc8meaabaqaciaacaGaaeqabaWaaeGaeaaakeaaimaacqWFZestaaa@3790@, i.e., *M*_1_, *M*_2_, ..., *M*_*k*_. This task can be solved by existing pattern matching algorithms. In the second step, we do positional joins on the pos-lists of the simple motifs. Let P
 MathType@MTEF@5@5@+=feaafiart1ev1aaatCvAUfKttLearuWrP9MDH5MBPbIqV92AaeXatLxBI9gBamrtHrhAL1wy0L2yHvtyaeHbnfgDOvwBHrxAJfwnaebbnrfifHhDYfgasaacH8akY=wiFfYdH8Gipec8Eeeu0xXdbba9frFj0=OqFfea0dXdd9vqai=hGuQ8kuc9pgc9s8qqaq=dirpe0xb9q8qiLsFr0=vr0=vr0dc8meaabaqaciaacaGaaeqabaWaaeGaeaaakeaacqqGqbauaaa@3786@(*M*_*i *_[*l*_*i*_, *u*_*i*_] T
 MathType@MTEF@5@5@+=feaafiart1ev1aaatCvAUfKttLearuWrP9MDH5MBPbIqV92AaeXatLxBI9gBamrtHrhAL1wy0L2yHvtyaeHbnfgDOvwBHrxAJfwnaebbnrfifHhDYfgasaacH8akY=wiFfYdH8Gipec8Eeeu0xXdbba9frFj0=OqFfea0dXdd9vqai=hGuQ8kuc9pgc9s8qqaq=dirpe0xb9q8qiLsFr0=vr0=vr0dc8meaabaqaciaacaGaaeqabaWaaeGaeaaakeaaimaacqWFtepvaaa@3847@) be an intermediate pos-list, with simple motif *M*_*i *_as the head, and a suffix structured motif T
 MathType@MTEF@5@5@+=feaafiart1ev1aaatCvAUfKttLearuWrP9MDH5MBPbIqV92AaeXatLxBI9gBamrtHrhAL1wy0L2yHvtyaeHbnfgDOvwBHrxAJfwnaebbnrfifHhDYfgasaacH8akY=wiFfYdH8Gipec8Eeeu0xXdbba9frFj0=OqFfea0dXdd9vqai=hGuQ8kuc9pgc9s8qqaq=dirpe0xb9q8qiLsFr0=vr0=vr0dc8meaabaqaciaacaGaaeqabaWaaeGaeaaakeaaimaacqWFtepvaaa@3847@ = *M*_*i*+1 _⋯ *M*_*k *_as tail. Since P
 MathType@MTEF@5@5@+=feaafiart1ev1aaatCvAUfKttLearuWrP9MDH5MBPbIqV92AaeXatLxBI9gBamrtHrhAL1wy0L2yHvtyaeHbnfgDOvwBHrxAJfwnaebbnrfifHhDYfgasaacH8akY=wiFfYdH8Gipec8Eeeu0xXdbba9frFj0=OqFfea0dXdd9vqai=hGuQ8kuc9pgc9s8qqaq=dirpe0xb9q8qiLsFr0=vr0=vr0dc8meaabaqaciaacaGaaeqabaWaaeGaeaaakeaacqqGqbauaaa@3786@(*M*_*i*_) stores only the start positions, we need convert them into end positions to check the gap constraints. There are two cases to consider.

#### Exact matching

**Figure 7:**
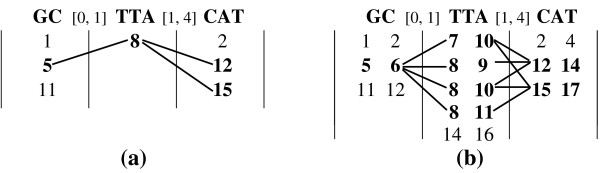
**Simple Motif Positional Joins Example**. The figure shows an example of positional joins on simple motifs, using the motif from Table 4.

Many algorithms [11-14] exist for exact pattern matching. Like in SMARTFINDER we use a lazy suffix tree [11] to extract the pos-lists for all simple motifs. The matching occurrences are sorted after extracting them from the suffix tree to obtain the pos-list in sorted order. For an intermediate pattern *M*_*i *_[*l*_*i*_, *u*_*i*_]T
 MathType@MTEF@5@5@+=feaafiart1ev1aaatCvAUfKttLearuWrP9MDH5MBPbIqV92AaeXatLxBI9gBamrtHrhAL1wy0L2yHvtyaeHbnfgDOvwBHrxAJfwnaebbnrfifHhDYfgasaacH8akY=wiFfYdH8Gipec8Eeeu0xXdbba9frFj0=OqFfea0dXdd9vqai=hGuQ8kuc9pgc9s8qqaq=dirpe0xb9q8qiLsFr0=vr0=vr0dc8meaabaqaciaacaGaaeqabaWaaeGaeaaakeaaimaacqWFtepvaaa@3847@, each start position *s *∈ P
 MathType@MTEF@5@5@+=feaafiart1ev1aaatCvAUfKttLearuWrP9MDH5MBPbIqV92AaeXatLxBI9gBamrtHrhAL1wy0L2yHvtyaeHbnfgDOvwBHrxAJfwnaebbnrfifHhDYfgasaacH8akY=wiFfYdH8Gipec8Eeeu0xXdbba9frFj0=OqFfea0dXdd9vqai=hGuQ8kuc9pgc9s8qqaq=dirpe0xb9q8qiLsFr0=vr0=vr0dc8meaabaqaciaacaGaaeqabaWaaeGaeaaakeaacqqGqbauaaa@3786@(*M*_*i *_[*l*_*i*_, *u*_*i*_]T
 MathType@MTEF@5@5@+=feaafiart1ev1aaatCvAUfKttLearuWrP9MDH5MBPbIqV92AaeXatLxBI9gBamrtHrhAL1wy0L2yHvtyaeHbnfgDOvwBHrxAJfwnaebbnrfifHhDYfgasaacH8akY=wiFfYdH8Gipec8Eeeu0xXdbba9frFj0=OqFfea0dXdd9vqai=hGuQ8kuc9pgc9s8qqaq=dirpe0xb9q8qiLsFr0=vr0=vr0dc8meaabaqaciaacaGaaeqabaWaaeGaeaaakeaaimaacqWFtepvaaa@3847@) is converted into an end position *s *+ |*M*_*i*_| - 1. Figure 7(a) shows an example of the pos-list join using exact matches for simple motifs. Each column shows the pos-list for a simple motif in the structured motif from Table 4. We first join the pos-lists of TTA and CAT, checking for gap range [1,4]. The start position 8 ∈ P
 MathType@MTEF@5@5@+=feaafiart1ev1aaatCvAUfKttLearuWrP9MDH5MBPbIqV92AaeXatLxBI9gBamrtHrhAL1wy0L2yHvtyaeHbnfgDOvwBHrxAJfwnaebbnrfifHhDYfgasaacH8akY=wiFfYdH8Gipec8Eeeu0xXdbba9frFj0=OqFfea0dXdd9vqai=hGuQ8kuc9pgc9s8qqaq=dirpe0xb9q8qiLsFr0=vr0=vr0dc8meaabaqaciaacaGaaeqabaWaaeGaeaaakeaacqqGqbauaaa@3786@(*TTA*) is converted to end position 8 + 3 - 1 = 10. We find that both positions 12 and 15 lie within the minimum and maximum gap range (indicated by the links), and thus 8 is retained in the resulting pos-list. Likewise 5 is in the final pos-list, since after obtaining its end position 5 + 2 - 1 = 6, we find *d *= 8 - 6 - 1 = 1 ∈ [0, 1].

#### Approximate matching

Several algorithms [8, 12, 15–18] exist for approximate pattern matching. For consistency, we used Sellers' dynamic programming algorithm [26], as implemented in SMARTFINDER, to extract the pos-lists for all simple motifs with approximate matches. This algorithm is not optimal and it can be replaced by more efficient ones [16-18]. Since we allow a specific Levenshtein distance [8] (i.e., insertions, deletions and substitutions) between the occurrences and the motif, the length of the occurrences can be different from the component length |*M*_*i*_|. Thus we augment the pos-list to explicitly store the end position, in addition to the start position, for each occurrence. Figure 7(b) shows how the pos-list joins work for approximate matches of simple motifs. In the structured motif from Table 4, we consider the exact matches of GC and CAT, and the approximate matches of TTA within Levenshtein distance of 1. Each column in (b) shows the pos-list of a simple motif: the left sub-column is a list of its start positions and the right sub-column is a list of its end positions. We first join the pos-lists of TTA and CAT, checking for gap range [1,4]. We compare the end positions of TTA and the start positions of CAT and find that the pairs (9,12), (10,12), (10,15), and (11,15) all lie within the gap range (indicated by the links), and thus the pairs, (7, 10), (8, 9), (8, 10) and (8, 11) are retained in the resulting pos-list. Likewise (5, 6) is in the final pos-list, since after comparing the end position of GC, 6, with the start position of TTA, 7 and 8, we find *d *= 7 - 6 - 1 = 0 ∈ [0,1] and *d *= 8 - 6 - 1 = 1 ∈ [0, 1].

### Structured profile search

Having outlined our approach for structured pattern search, here we tackle the problem of structured profile search. The profile (also called a position weight matrix) for a structured motif ℳ
 MathType@MTEF@5@5@+=feaafiart1ev1aaatCvAUfKttLearuWrP9MDH5MBPbIqV92AaeXatLxBI9gBamrtHrhAL1wy0L2yHvtyaeHbnfgDOvwBHrxAJfwnaebbnrfifHhDYfgasaacH8akY=wiFfYdH8Gipec8Eeeu0xXdbba9frFj0=OqFfea0dXdd9vqai=hGuQ8kuc9pgc9s8qqaq=dirpe0xb9q8qiLsFr0=vr0=vr0dc8meaabaqaciaacaGaaeqabaWaaeGaeaaakeaaimaacqWFZestaaa@3790@ gives for each position in each component, the frequency of occurrence for each symbol in Σ_DNA_, i.e., ℳ
 MathType@MTEF@5@5@+=feaafiart1ev1aaatCvAUfKttLearuWrP9MDH5MBPbIqV92AaeXatLxBI9gBamrtHrhAL1wy0L2yHvtyaeHbnfgDOvwBHrxAJfwnaebbnrfifHhDYfgasaacH8akY=wiFfYdH8Gipec8Eeeu0xXdbba9frFj0=OqFfea0dXdd9vqai=hGuQ8kuc9pgc9s8qqaq=dirpe0xb9q8qiLsFr0=vr0=vr0dc8meaabaqaciaacaGaaeqabaWaaeGaeaaakeaaimaacqWFZestaaa@3790@_*xj *_gives how often symbol *x *∈ Σ_DNA _occurs at position *j*. Note that, as in the case of pattern motifs, for a structured profile motif ℳ
 MathType@MTEF@5@5@+=feaafiart1ev1aaatCvAUfKttLearuWrP9MDH5MBPbIqV92AaeXatLxBI9gBamrtHrhAL1wy0L2yHvtyaeHbnfgDOvwBHrxAJfwnaebbnrfifHhDYfgasaacH8akY=wiFfYdH8Gipec8Eeeu0xXdbba9frFj0=OqFfea0dXdd9vqai=hGuQ8kuc9pgc9s8qqaq=dirpe0xb9q8qiLsFr0=vr0=vr0dc8meaabaqaciaacaGaaeqabaWaaeGaeaaakeaaimaacqWFZestaaa@3790@, we have to specify the gap constraints between its simple profile motif components. Profiles are able to better capture the variability in the motifs, since they capture position specific statistics on the symbols, and as such can retain more valuable information than the pattern representation.

Given a profile structured motif ℳ
 MathType@MTEF@5@5@+=feaafiart1ev1aaatCvAUfKttLearuWrP9MDH5MBPbIqV92AaeXatLxBI9gBamrtHrhAL1wy0L2yHvtyaeHbnfgDOvwBHrxAJfwnaebbnrfifHhDYfgasaacH8akY=wiFfYdH8Gipec8Eeeu0xXdbba9frFj0=OqFfea0dXdd9vqai=hGuQ8kuc9pgc9s8qqaq=dirpe0xb9q8qiLsFr0=vr0=vr0dc8meaabaqaciaacaGaaeqabaWaaeGaeaaakeaaimaacqWFZestaaa@3790@, and a user-specified match/score threshold λ, the goal of structured profile search is to enumerate all structured *patterns *that match the profile motif above the threshold λ. Our approach to profile motif search consists of the following two steps: a) convert the raw frequency profile into a relative profile weighted by information content at each position, b) enumerate occurrences matching the profile.

#### Weighted profile creation

Given the initial "raw" frequency profile for the structured motif ℳ
 MathType@MTEF@5@5@+=feaafiart1ev1aaatCvAUfKttLearuWrP9MDH5MBPbIqV92AaeXatLxBI9gBamrtHrhAL1wy0L2yHvtyaeHbnfgDOvwBHrxAJfwnaebbnrfifHhDYfgasaacH8akY=wiFfYdH8Gipec8Eeeu0xXdbba9frFj0=OqFfea0dXdd9vqai=hGuQ8kuc9pgc9s8qqaq=dirpe0xb9q8qiLsFr0=vr0=vr0dc8meaabaqaciaacaGaaeqabaWaaeGaeaaakeaaimaacqWFZestaaa@3790@, we first convert it into a weighted profile as follows:

fxj=ℳxj+px∑y∈ΣDNA(ℳyj+py),W′xj=ln⁡(fxjpx)     (1)
 MathType@MTEF@5@5@+=feaafiart1ev1aaatCvAUfKttLearuWrP9MDH5MBPbIqV92AaeXatLxBI9gBamrtHrhAL1wy0L2yHvtyaeHbnfgDOvwBHrxAJfwnaebbnrfifHhDYfgasaacH8akY=wiFfYdH8Gipec8Eeeu0xXdbba9frFj0=OqFfea0dXdd9vqai=hGuQ8kuc9pgc9s8qqaq=dirpe0xb9q8qiLsFr0=vr0=vr0dc8meaabaqaciaacaGaaeqabaWaaeGaeaaakeaafaqabeqacaaabaGaemOzay2aaSbaaSqaaiabdIha4jabdQgaQbqabaGccqGH9aqpdaWcaaqaaGWaaiab=ntinnaaBaaaleaacqWG4baEcqWGQbGAaeqaaOGaey4kaSIaemiCaa3aaSbaaSqaaiabdIha4bqabaaakeaadaaeqaqaaiabcIcaOiab=ntinnaaBaaaleaacqWG5bqEcqWGQbGAaeqaaOGaey4kaSIaemiCaa3aaSbaaSqaaiabdMha5bqabaGccqGGPaqkaSqaaiabdMha5jabgIGiolabfo6atnaaBaaameaacqqGebarcqqGobGtcqqGbbqqaeqaaaWcbeqdcqGHris5aaaakiabcYcaSaqaaiqb=zr8xzaafaWaaSbaaSqaaiabdIha4jabdQgaQbqabaGccqGH9aqpcyGGSbaBcqGGUbGBdaqadaqaamaalaaabaGaemOzay2aaSbaaSqaaiabdIha4jabdQgaQbqabaaakeaacqWGWbaCdaWgaaWcbaGaemiEaGhabeaaaaaakiaawIcacaGLPaaaaaGaaCzcaiaaxMaadaqadaqaaiabigdaXaGaayjkaiaawMcaaaaa@6E55@

where ℳ
 MathType@MTEF@5@5@+=feaafiart1ev1aaatCvAUfKttLearuWrP9MDH5MBPbIqV92AaeXatLxBI9gBamrtHrhAL1wy0L2yHvtyaeHbnfgDOvwBHrxAJfwnaebbnrfifHhDYfgasaacH8akY=wiFfYdH8Gipec8Eeeu0xXdbba9frFj0=OqFfea0dXdd9vqai=hGuQ8kuc9pgc9s8qqaq=dirpe0xb9q8qiLsFr0=vr0=vr0dc8meaabaqaciaacaGaaeqabaWaaeGaeaaakeaaimaacqWFZestaaa@3790@_*xj *_gives the absolute frequency of symbol *x *∈ Σ_DNA _at position *j *in the structured motif ℳ
 MathType@MTEF@5@5@+=feaafiart1ev1aaatCvAUfKttLearuWrP9MDH5MBPbIqV92AaeXatLxBI9gBamrtHrhAL1wy0L2yHvtyaeHbnfgDOvwBHrxAJfwnaebbnrfifHhDYfgasaacH8akY=wiFfYdH8Gipec8Eeeu0xXdbba9frFj0=OqFfea0dXdd9vqai=hGuQ8kuc9pgc9s8qqaq=dirpe0xb9q8qiLsFr0=vr0=vr0dc8meaabaqaciaacaGaaeqabaWaaeGaeaaakeaaimaacqWFZestaaa@3790@, for 1 ≤ *j *≤ |ℳ
 MathType@MTEF@5@5@+=feaafiart1ev1aaatCvAUfKttLearuWrP9MDH5MBPbIqV92AaeXatLxBI9gBamrtHrhAL1wy0L2yHvtyaeHbnfgDOvwBHrxAJfwnaebbnrfifHhDYfgasaacH8akY=wiFfYdH8Gipec8Eeeu0xXdbba9frFj0=OqFfea0dXdd9vqai=hGuQ8kuc9pgc9s8qqaq=dirpe0xb9q8qiLsFr0=vr0=vr0dc8meaabaqaciaacaGaaeqabaWaaeGaeaaakeaaimaacqWFZestaaa@3790@|; *f*_*xj *_represents the relative frequency of symbol *x *at position *j *in the motif; *p*_*x *_(or *p*_*y*_) denotes the prior (background) probability of symbol *x *(or *y*) ∈ Σ_DNA_; W′xj
 MathType@MTEF@5@5@+=feaafiart1ev1aaatCvAUfKttLearuWrP9MDH5MBPbIqV92AaeXatLxBI9gBamrtHrhAL1wy0L2yHvtyaeHbnfgDOvwBHrxAJfwnaebbnrfifHhDYfgasaacH8akY=wiFfYdH8Gipec8Eeeu0xXdbba9frFj0=OqFfea0dXdd9vqai=hGuQ8kuc9pgc9s8qqaq=dirpe0xb9q8qiLsFr0=vr0=vr0dc8meaabaqaciaacaGaaeqabaWaaeGaeaaakeaaimaacuWFwe=vgaqbamaaBaaaleaacqWG4baEcqWGQbGAaeqaaaaa@3B5B@ is the weight (log-likelihood) of observing symbol *x *at position *j*.

Whereas the weights computed above give the likelihood of observing a given symbol in a given position they do not account for the degree to which some symbols are conserved at some positions. We can adjust the weights by considering the information content at each position. The *information content *for a profile is given as:

ℐxj=fxjln⁡(fxj)−pxln⁡(px),ℐj=∑x∈ΣDNAℐxj,ℐ=∑j=1|ℳ|ℐj     (2)
 MathType@MTEF@5@5@+=feaafiart1ev1aaatCvAUfKttLearuWrP9MDH5MBPbIqV92AaeXatLxBI9gBamrtHrhAL1wy0L2yHvtyaeHbnfgDOvwBHrxAJfwnaebbnrfifHhDYfgasaacH8akY=wiFfYdH8Gipec8Eeeu0xXdbba9frFj0=OqFfea0dXdd9vqai=hGuQ8kuc9pgc9s8qqaq=dirpe0xb9q8qiLsFr0=vr0=vr0dc8meaabaqaciaacaGaaeqabaWaaeGaeaaakeaafaqabeqadaaabaacdaGae8heHK0aaSbaaSqaaiabdIha4jabdQgaQbqabaGccqGH9aqpcqWGMbGzdaWgaaWcbaGaemiEaGNaemOAaOgabeaakiGbcYgaSjabc6gaUjabcIcaOiabdAgaMnaaBaaaleaacqWG4baEcqWGQbGAaeqaaOGaeiykaKIaeyOeI0IaemiCaa3aaSbaaSqaaiabdIha4bqabaGccyGGSbaBcqGGUbGBcqGGOaakcqWGWbaCdaWgaaWcbaGaemiEaGhabeaakiabcMcaPiabcYcaSaqaaiab=brijnaaBaaaleaacqWGQbGAaeqaaOGaeyypa0ZaaabuaeaacqWFqessdaWgaaWcbaGaemiEaGNaemOAaOgabeaakiabcYcaSaWcbaGaemiEaGNaeyicI4Saeu4Odm1aaSbaaWqaaiabbseaejabb6eaojabbgeabbqabaaaleqaniabggHiLdaakeaacqWFqesscqGH9aqpdaaeWbqaaiab=brijnaaBaaaleaacqWGQbGAaeqaaaqaaiabdQgaQjabg2da9iabigdaXaqaaiabcYha8jab=ntinjabcYha8bqdcqGHris5aaaakiaaxMaacaWLjaWaaeWaaeaacqaIYaGmaiaawIcacaGLPaaaaaa@79E3@

where ℐ
 MathType@MTEF@5@5@+=feaafiart1ev1aaatCvAUfKttLearuWrP9MDH5MBPbIqV92AaeXatLxBI9gBamrtHrhAL1wy0L2yHvtyaeHbnfgDOvwBHrxAJfwnaebbnrfifHhDYfgasaacH8akY=wiFfYdH8Gipec8Eeeu0xXdbba9frFj0=OqFfea0dXdd9vqai=hGuQ8kuc9pgc9s8qqaq=dirpe0xb9q8qiLsFr0=vr0=vr0dc8meaabaqaciaacaGaaeqabaWaaeGaeaaakeaaimaacqWFqessaaa@3769@_*xj *_is the information content of symbol *x *at position *j*; ℐ
 MathType@MTEF@5@5@+=feaafiart1ev1aaatCvAUfKttLearuWrP9MDH5MBPbIqV92AaeXatLxBI9gBamrtHrhAL1wy0L2yHvtyaeHbnfgDOvwBHrxAJfwnaebbnrfifHhDYfgasaacH8akY=wiFfYdH8Gipec8Eeeu0xXdbba9frFj0=OqFfea0dXdd9vqai=hGuQ8kuc9pgc9s8qqaq=dirpe0xb9q8qiLsFr0=vr0=vr0dc8meaabaqaciaacaGaaeqabaWaaeGaeaaakeaaimaacqWFqessaaa@3769@_*j *_is the information content over all bases at position *j*; and ℐ
 MathType@MTEF@5@5@+=feaafiart1ev1aaatCvAUfKttLearuWrP9MDH5MBPbIqV92AaeXatLxBI9gBamrtHrhAL1wy0L2yHvtyaeHbnfgDOvwBHrxAJfwnaebbnrfifHhDYfgasaacH8akY=wiFfYdH8Gipec8Eeeu0xXdbba9frFj0=OqFfea0dXdd9vqai=hGuQ8kuc9pgc9s8qqaq=dirpe0xb9q8qiLsFr0=vr0=vr0dc8meaabaqaciaacaGaaeqabaWaaeGaeaaakeaaimaacqWFqessaaa@3769@ is the information content of the entire profile. To allow mismatches at less conserved positions to be more easily tolerated than those at highly conserved positions, we multiply each weight W′xj
 MathType@MTEF@5@5@+=feaafiart1ev1aaatCvAUfKttLearuWrP9MDH5MBPbIqV92AaeXatLxBI9gBamrtHrhAL1wy0L2yHvtyaeHbnfgDOvwBHrxAJfwnaebbnrfifHhDYfgasaacH8akY=wiFfYdH8Gipec8Eeeu0xXdbba9frFj0=OqFfea0dXdd9vqai=hGuQ8kuc9pgc9s8qqaq=dirpe0xb9q8qiLsFr0=vr0=vr0dc8meaabaqaciaacaGaaeqabaWaaeGaeaaakeaaimaacuWFwe=vgaqbamaaBaaaleaacqWG4baEcqWGQbGAaeqaaaaa@3B5B@ by ℐ
 MathType@MTEF@5@5@+=feaafiart1ev1aaatCvAUfKttLearuWrP9MDH5MBPbIqV92AaeXatLxBI9gBamrtHrhAL1wy0L2yHvtyaeHbnfgDOvwBHrxAJfwnaebbnrfifHhDYfgasaacH8akY=wiFfYdH8Gipec8Eeeu0xXdbba9frFj0=OqFfea0dXdd9vqai=hGuQ8kuc9pgc9s8qqaq=dirpe0xb9q8qiLsFr0=vr0=vr0dc8meaabaqaciaacaGaaeqabaWaaeGaeaaakeaaimaacqWFqessaaa@3769@_*j*_, which is larger for more conserved positions. As a result, the corrected weight of each element in the profile becomes:

Wxj=ℐjW′xj=ℐjln⁡(fxjpx)     (3)
 MathType@MTEF@5@5@+=feaafiart1ev1aaatCvAUfKttLearuWrP9MDH5MBPbIqV92AaeXatLxBI9gBamrtHrhAL1wy0L2yHvtyaeHbnfgDOvwBHrxAJfwnaebbnrfifHhDYfgasaacH8akY=wiFfYdH8Gipec8Eeeu0xXdbba9frFj0=OqFfea0dXdd9vqai=hGuQ8kuc9pgc9s8qqaq=dirpe0xb9q8qiLsFr0=vr0=vr0dc8meaabaqaciaacaGaaeqabaWaaeGaeaaakeaaimaacqWFwe=vdaWgaaWcbaGaemiEaGNaemOAaOgabeaakiabg2da9iab=brijnaaBaaaleaacqWGQbGAaeqaaOGaf8NfXFLbauaadaWgaaWcbaGaemiEaGNaemOAaOgabeaakiabg2da9iab=brijnaaBaaaleaacqWGQbGAaeqaaOGagiiBaWMaeiOBa42aaeWaaeaadaWcaaqaaiabdAgaMnaaBaaaleaacqWG4baEcqWGQbGAaeqaaaGcbaGaemiCaa3aaSbaaSqaaiabdIha4bqabaaaaaGccaGLOaGaayzkaaGaaCzcaiaaxMaadaqadaqaaiabiodaZaGaayjkaiaawMcaaaaa@571D@

Given the initial profile motif ℳ
 MathType@MTEF@5@5@+=feaafiart1ev1aaatCvAUfKttLearuWrP9MDH5MBPbIqV92AaeXatLxBI9gBamrtHrhAL1wy0L2yHvtyaeHbnfgDOvwBHrxAJfwnaebbnrfifHhDYfgasaacH8akY=wiFfYdH8Gipec8Eeeu0xXdbba9frFj0=OqFfea0dXdd9vqai=hGuQ8kuc9pgc9s8qqaq=dirpe0xb9q8qiLsFr0=vr0=vr0dc8meaabaqaciaacaGaaeqabaWaaeGaeaaakeaaimaacqWFZestaaa@3790@ we obtain its corresponding information content weighted profile W
 MathType@MTEF@5@5@+=feaafiart1ev1aaatCvAUfKttLearuWrP9MDH5MBPbIqV92AaeXatLxBI9gBamrtHrhAL1wy0L2yHvtyaeHbnfgDOvwBHrxAJfwnaebbnrfifHhDYfgasaacH8akY=wiFfYdH8Gipec8Eeeu0xXdbba9frFj0=OqFfea0dXdd9vqai=hGuQ8kuc9pgc9s8qqaq=dirpe0xb9q8qiLsFr0=vr0=vr0dc8meaabaqaciaacaGaaeqabaWaaeGaeaaakeaaimaacqWFwe=vaaa@384D@ for use in the enumeration step. Our weighting approach has some differences with respect to previous ones [19-22]. For instance, we consider the prior probability of each base for the calculation of the positional weight and information content. That is necessary because some bases (i.e. C and G) occur more frequently than others (i.e. A and T). Also we use relative frequency instead of the absolute one, so as to compare with the prior probability. However, note that, in general, SMOTIF is flexible enough to handle any user specified profile.

**Figure 8:**
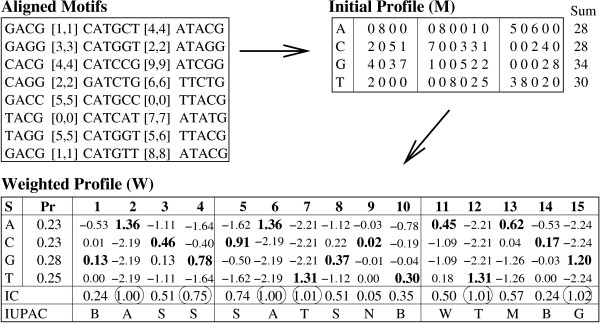
**Structured Profile**. A set of 8 aligned structured motifs yield the initial profile motif ℳ
 MathType@MTEF@5@5@+=feaafiart1ev1aaatCvAUfKttLearuWrP9MDH5MBPbIqV92AaeXatLxBI9gBamrtHrhAL1wy0L2yHvtyaeHbnfgDOvwBHrxAJfwnaebbnrfifHhDYfgasaacH8akY=wiFfYdH8Gipec8Eeeu0xXdbba9frFj0=OqFfea0dXdd9vqai=hGuQ8kuc9pgc9s8qqaq=dirpe0xb9q8qiLsFr0=vr0=vr0dc8meaabaqaciaacaGaaeqabaWaaeGaeaaakeaaimaacqWFZestaaa@3790@ = *M*_1 _[0, 5] *M*_2 _[0, 9] *M*_3_, (where |*M*_1_| = 4, |*M*_2_| = 6, and |*M*_3_| = 5), which is converted into the information content weighted profile W
 MathType@MTEF@5@5@+=feaafiart1ev1aaatCvAUfKttLearuWrP9MDH5MBPbIqV92AaeXatLxBI9gBamrtHrhAL1wy0L2yHvtyaeHbnfgDOvwBHrxAJfwnaebbnrfifHhDYfgasaacH8akY=wiFfYdH8Gipec8Eeeu0xXdbba9frFj0=OqFfea0dXdd9vqai=hGuQ8kuc9pgc9s8qqaq=dirpe0xb9q8qiLsFr0=vr0=vr0dc8meaabaqaciaacaGaaeqabaWaaeGaeaaakeaaimaacqWFwe=vaaa@384D@. The highest weights at each position are given in bold. The penultimate row shows the position specific information content (IC), and the *core *positions with highest IC are given in brackets. The last row shows the IUPAC symbol that best captures the position specific symbol occurrences.

Figure 8 shows an example of computing a profile from a set of aligned structured motifs. There are 8 aligned motifs with different gaps between components. We first obtain the initial frequencies of each symbol *x *∈ Σ_DNA _in each position *j *(ℳ
 MathType@MTEF@5@5@+=feaafiart1ev1aaatCvAUfKttLearuWrP9MDH5MBPbIqV92AaeXatLxBI9gBamrtHrhAL1wy0L2yHvtyaeHbnfgDOvwBHrxAJfwnaebbnrfifHhDYfgasaacH8akY=wiFfYdH8Gipec8Eeeu0xXdbba9frFj0=OqFfea0dXdd9vqai=hGuQ8kuc9pgc9s8qqaq=dirpe0xb9q8qiLsFr0=vr0=vr0dc8meaabaqaciaacaGaaeqabaWaaeGaeaaakeaaimaacqWFZestaaa@3790@_*xj*_), as well as its background probability (*p*_*x*_). For example, in position *j *= 1, we have 4 occurrences of *G*, i.e., ℳ
 MathType@MTEF@5@5@+=feaafiart1ev1aaatCvAUfKttLearuWrP9MDH5MBPbIqV92AaeXatLxBI9gBamrtHrhAL1wy0L2yHvtyaeHbnfgDOvwBHrxAJfwnaebbnrfifHhDYfgasaacH8akY=wiFfYdH8Gipec8Eeeu0xXdbba9frFj0=OqFfea0dXdd9vqai=hGuQ8kuc9pgc9s8qqaq=dirpe0xb9q8qiLsFr0=vr0=vr0dc8meaabaqaciaacaGaaeqabaWaaeGaeaaakeaaimaacqWFZestaaa@3790@_*G*1 _= 4. Also, the background probability of *G *in the aligned motif is *p*_*G *_= 34/120 = 0.28 (note that the background probabilities can be obtained using different means, for example, from the entire set of DNA sequences, and not just the aligned motifs). Plugging in these values into Equations 1, 2 and 3 yield the final position specific weights (under each column) in the weighted profile W
 MathType@MTEF@5@5@+=feaafiart1ev1aaatCvAUfKttLearuWrP9MDH5MBPbIqV92AaeXatLxBI9gBamrtHrhAL1wy0L2yHvtyaeHbnfgDOvwBHrxAJfwnaebbnrfifHhDYfgasaacH8akY=wiFfYdH8Gipec8Eeeu0xXdbba9frFj0=OqFfea0dXdd9vqai=hGuQ8kuc9pgc9s8qqaq=dirpe0xb9q8qiLsFr0=vr0=vr0dc8meaabaqaciaacaGaaeqabaWaaeGaeaaakeaaimaacqWFwe=vaaa@384D@ shown in Figure 8, e.g., W
 MathType@MTEF@5@5@+=feaafiart1ev1aaatCvAUfKttLearuWrP9MDH5MBPbIqV92AaeXatLxBI9gBamrtHrhAL1wy0L2yHvtyaeHbnfgDOvwBHrxAJfwnaebbnrfifHhDYfgasaacH8akY=wiFfYdH8Gipec8Eeeu0xXdbba9frFj0=OqFfea0dXdd9vqai=hGuQ8kuc9pgc9s8qqaq=dirpe0xb9q8qiLsFr0=vr0=vr0dc8meaabaqaciaacaGaaeqabaWaaeGaeaaakeaaimaacqWFwe=vaaa@384D@_*G*1 _= 0.13. The figure also shows the information content for each position, as well as the IUPAC symbol that best captures the symbol distribution in each position.

#### Profile scoring

Given the weighted profile W
 MathType@MTEF@5@5@+=feaafiart1ev1aaatCvAUfKttLearuWrP9MDH5MBPbIqV92AaeXatLxBI9gBamrtHrhAL1wy0L2yHvtyaeHbnfgDOvwBHrxAJfwnaebbnrfifHhDYfgasaacH8akY=wiFfYdH8Gipec8Eeeu0xXdbba9frFj0=OqFfea0dXdd9vqai=hGuQ8kuc9pgc9s8qqaq=dirpe0xb9q8qiLsFr0=vr0=vr0dc8meaabaqaciaacaGaaeqabaWaaeGaeaaakeaaimaacqWFwe=vaaa@384D@ for a structured motif ℳ
 MathType@MTEF@5@5@+=feaafiart1ev1aaatCvAUfKttLearuWrP9MDH5MBPbIqV92AaeXatLxBI9gBamrtHrhAL1wy0L2yHvtyaeHbnfgDOvwBHrxAJfwnaebbnrfifHhDYfgasaacH8akY=wiFfYdH8Gipec8Eeeu0xXdbba9frFj0=OqFfea0dXdd9vqai=hGuQ8kuc9pgc9s8qqaq=dirpe0xb9q8qiLsFr0=vr0=vr0dc8meaabaqaciaacaGaaeqabaWaaeGaeaaakeaaimaacqWFZestaaa@3790@, a sequence *S *∈ S
 MathType@MTEF@5@5@+=feaafiart1ev1aaatCvAUfKttLearuWrP9MDH5MBPbIqV92AaeXatLxBI9gBamrtHrhAL1wy0L2yHvtyaeHbnfgDOvwBHrxAJfwnaebbnrfifHhDYfgasaacH8akY=wiFfYdH8Gipec8Eeeu0xXdbba9frFj0=OqFfea0dXdd9vqai=hGuQ8kuc9pgc9s8qqaq=dirpe0xb9q8qiLsFr0=vr0=vr0dc8meaabaqaciaacaGaaeqabaWaaeGaeaaakeaaimaacqWFse=uaaa@3845@, and any subsequence *S' *of *S *(not necessarily consecutive) of length |ℳ
 MathType@MTEF@5@5@+=feaafiart1ev1aaatCvAUfKttLearuWrP9MDH5MBPbIqV92AaeXatLxBI9gBamrtHrhAL1wy0L2yHvtyaeHbnfgDOvwBHrxAJfwnaebbnrfifHhDYfgasaacH8akY=wiFfYdH8Gipec8Eeeu0xXdbba9frFj0=OqFfea0dXdd9vqai=hGuQ8kuc9pgc9s8qqaq=dirpe0xb9q8qiLsFr0=vr0=vr0dc8meaabaqaciaacaGaaeqabaWaaeGaeaaakeaaimaacqWFZestaaa@3790@|, that satisfies the gap constraints, the *profile score *for subsequence *S' *is computed as the sum of weights of its symbols at each position in the profile, given as: ℛ(S′)=∑j=1|ℳ|WS′[j]j
 MathType@MTEF@5@5@+=feaafiart1ev1aaatCvAUfKttLearuWrP9MDH5MBPbIqV92AaeXatLxBI9gBamrtHrhAL1wy0L2yHvtyaeHbnfgDOvwBHrxAJfwnaebbnrfifHhDYfgasaacH8akY=wiFfYdH8Gipec8Eeeu0xXdbba9frFj0=OqFfea0dXdd9vqai=hGuQ8kuc9pgc9s8qqaq=dirpe0xb9q8qiLsFr0=vr0=vr0dc8meaabaqaciaacaGaaeqabaWaaeGaeaaakeaaimaacqWFBeIucqGGOaakcuWGtbWugaqbaiabcMcaPiabg2da9maaqadabaGae8NfXF1aaSbaaSqaaiqbdofatzaafaGaei4waSLaemOAaOMaeiyxa0LaemOAaOgabeaaaeaacqWGQbGAcqGH9aqpcqaIXaqmaeaacqGG8baFcqWFZestcqGG8baFa0GaeyyeIuoaaaa@4D64@ (note that W
 MathType@MTEF@5@5@+=feaafiart1ev1aaatCvAUfKttLearuWrP9MDH5MBPbIqV92AaeXatLxBI9gBamrtHrhAL1wy0L2yHvtyaeHbnfgDOvwBHrxAJfwnaebbnrfifHhDYfgasaacH8akY=wiFfYdH8Gipec8Eeeu0xXdbba9frFj0=OqFfea0dXdd9vqai=hGuQ8kuc9pgc9s8qqaq=dirpe0xb9q8qiLsFr0=vr0=vr0dc8meaabaqaciaacaGaaeqabaWaaeGaeaaakeaaimaacqWFwe=vaaa@384D@_*S' *[*j*]*j *_gives the weight of symbol *S' *[*j*] at position *j *in the profile motif). In order to check whether *S' *is a potential binding site, we compare its profile score with the user-specified threshold, λ ∈ [0,1]. To ensure that the profile score lies between 0 and 1, we have to normalize it. Let W
 MathType@MTEF@5@5@+=feaafiart1ev1aaatCvAUfKttLearuWrP9MDH5MBPbIqV92AaeXatLxBI9gBamrtHrhAL1wy0L2yHvtyaeHbnfgDOvwBHrxAJfwnaebbnrfifHhDYfgasaacH8akY=wiFfYdH8Gipec8Eeeu0xXdbba9frFj0=OqFfea0dXdd9vqai=hGuQ8kuc9pgc9s8qqaq=dirpe0xb9q8qiLsFr0=vr0=vr0dc8meaabaqaciaacaGaaeqabaWaaeGaeaaakeaaimaacqWFwe=vaaa@384D@^min ^and W
 MathType@MTEF@5@5@+=feaafiart1ev1aaatCvAUfKttLearuWrP9MDH5MBPbIqV92AaeXatLxBI9gBamrtHrhAL1wy0L2yHvtyaeHbnfgDOvwBHrxAJfwnaebbnrfifHhDYfgasaacH8akY=wiFfYdH8Gipec8Eeeu0xXdbba9frFj0=OqFfea0dXdd9vqai=hGuQ8kuc9pgc9s8qqaq=dirpe0xb9q8qiLsFr0=vr0=vr0dc8meaabaqaciaacaGaaeqabaWaaeGaeaaakeaaimaacqWFwe=vaaa@384D@^max ^be the minimum and maximum weights, and let *μ *and *σ *be the mean and standard deviation of the weights, across all the positions in the weighted profile. The normalized profile score ℛ
 MathType@MTEF@5@5@+=feaafiart1ev1aaatCvAUfKttLearuWrP9MDH5MBPbIqV92AaeXatLxBI9gBamrtHrhAL1wy0L2yHvtyaeHbnfgDOvwBHrxAJfwnaebbnrfifHhDYfgasaacH8akY=wiFfYdH8Gipec8Eeeu0xXdbba9frFj0=OqFfea0dXdd9vqai=hGuQ8kuc9pgc9s8qqaq=dirpe0xb9q8qiLsFr0=vr0=vr0dc8meaabaqaciaacaGaaeqabaWaaeGaeaaakeaaimaacqWFBeIuaaa@377D@^*n *^can then be calculated using any of the following formulas:

(a). ℛn(S′)=ℛ(S′)Wmax⁡(b). ℛn(S′)=ℛ(S′)−Wmin⁡Wmax⁡−Wmin⁡(c). ℛn(S′)=ℛ(S′)−μ3σ+12     (4)
 MathType@MTEF@5@5@+=feaafiart1ev1aaatCvAUfKttLearuWrP9MDH5MBPbIqV92AaeXatLxBI9gBamrtHrhAL1wy0L2yHvtyaeHbnfgDOvwBHrxAJfwnaebbnrfifHhDYfgasaacH8akY=wiFfYdH8Gipec8Eeeu0xXdbba9frFj0=OqFfea0dXdd9vqai=hGuQ8kuc9pgc9s8qqaq=dirpe0xb9q8qiLsFr0=vr0=vr0dc8meaabaqaciaacaGaaeqabaWaaeGaeaaakeaafaqabeqadaaabaGaeiikaGIaemyyaeMaeiykaKIaeiOla4IaeeiiaaccdaGae83gHi1aaWbaaSqabeaacqWGUbGBaaGccqGGOaakcuWGtbWugaqbaiabcMcaPiabg2da9maalaaabaGae83gHiLaeiikaGIafm4uamLbauaacqGGPaqkaeaacqWFwe=vdaahaaWcbeqaaiGbc2gaTjabcggaHjabcIha4baaaaaakeaacqGGOaakcqWGIbGycqGGPaqkcqGGUaGlcqqGGaaicqWFBeIudaahaaWcbeqaaiabd6gaUbaakiabcIcaOiqbdofatzaafaGaeiykaKIaeyypa0ZaaSaaaeaacqWFBeIucqGGOaakcuWGtbWugaqbaiabcMcaPiabgkHiTiab=zr8xnaaCaaaleqabaGagiyBa0MaeiyAaKMaeiOBa4gaaaGcbaGae8NfXF1aaWbaaSqabeaacyGGTbqBcqGGHbqycqGG4baEaaGccqGHsislcqWFwe=vdaahaaWcbeqaaiGbc2gaTjabcMgaPjabc6gaUbaaaaaakeaacqGGOaakcqWGJbWycqGGPaqkcqGGUaGlcqqGGaaicqWFBeIudaahaaWcbeqaaiabd6gaUbaakiabcIcaOiqbdofatzaafaGaeiykaKIaeyypa0ZaaSaaaeaadaWcaaqaaiab=TrisjabcIcaOiqbdofatzaafaGaeiykaKIaeyOeI0ccciGae4hVd0gabaGaeG4mamJae43WdmhaaiabgUcaRiabigdaXaqaaiabikdaYaaaaaGaaCzcaiaaxMaadaqadaqaaiabisda0aGaayjkaiaawMcaaaaa@8B50@

Note that whereas Equations 4(a) and 4(b) are strictly in the range [0, 1], for 4(c) ℛ
 MathType@MTEF@5@5@+=feaafiart1ev1aaatCvAUfKttLearuWrP9MDH5MBPbIqV92AaeXatLxBI9gBamrtHrhAL1wy0L2yHvtyaeHbnfgDOvwBHrxAJfwnaebbnrfifHhDYfgasaacH8akY=wiFfYdH8Gipec8Eeeu0xXdbba9frFj0=OqFfea0dXdd9vqai=hGuQ8kuc9pgc9s8qqaq=dirpe0xb9q8qiLsFr0=vr0=vr0dc8meaabaqaciaacaGaaeqabaWaaeGaeaaakeaaimaacqWFBeIuaaa@377D@^*n *^is in the range [0, 1] only 99.7% of the time (within a range ± 3*σ *of the mean *μ*).

When applying the score threshold for an occurrence *S'*, we require that its normalized score is above the threshold λ. For example, for Equation 4(b),

ℛn(S′)=ℛ(S′)−Wmin⁡Wmax⁡−Wmin⁡≥λ⇒ℛ(S′)≥λ(Wmax⁡−Wmin⁡)+Wmin⁡=λn
 MathType@MTEF@5@5@+=feaafiart1ev1aaatCvAUfKttLearuWrP9MDH5MBPbIqV92AaeXatLxBI9gBamrtHrhAL1wy0L2yHvtyaeHbnfgDOvwBHrxAJfwnaebbnrfifHhDYfgasaacH8akY=wiFfYdH8Gipec8Eeeu0xXdbba9frFj0=OqFfea0dXdd9vqai=hGuQ8kuc9pgc9s8qqaq=dirpe0xb9q8qiLsFr0=vr0=vr0dc8meaabaqaciaacaGaaeqabaWaaeGaeaaakeaaimaacqWFBeIudaahaaWcbeqaaiabd6gaUbaakiabcIcaOiqbdofatzaafaGaeiykaKIaeyypa0ZaaSaaaeaacqWFBeIucqGGOaakcuWGtbWugaqbaiabcMcaPiabgkHiTiab=zr8xnaaCaaaleqabaGagiyBa0MaeiyAaKMaeiOBa4gaaaGcbaGae8NfXF1aaWbaaSqabeaacyGGTbqBcqGGHbqycqGG4baEaaGccqGHsislcqWFwe=vdaahaaWcbeqaaiGbc2gaTjabcMgaPjabc6gaUbaaaaGccqGHLjYScqaH7oaBcqGHshI3cqWFBeIucqGGOaakcuWGtbWugaqbaiabcMcaPiabgwMiZkabeU7aSjabcIcaOiab=zr8xnaaCaaaleqabaGagiyBa0MaeiyyaeMaeiiEaGhaaOGaeyOeI0Iae8NfXF1aaWbaaSqabeaacyGGTbqBcqGGPbqAcqGGUbGBaaGccqGGPaqkcqGHRaWkcqWFwe=vdaahaaWcbeqaaiGbc2gaTjabcMgaPjabc6gaUbaakiabg2da9iabeU7aSnaaCaaaleqabaGaemOBa4gaaaaa@7D84@

In other words, instead of normalizing the score for each match, we take λ^*n *^as the new normalized threshold for scoring the potential matches. Likewise we can get the new thresholds for Equations 4(a) and 4(c). For example, for 4(a) the normalized threshold would be λ^*n *^= λ · W
 MathType@MTEF@5@5@+=feaafiart1ev1aaatCvAUfKttLearuWrP9MDH5MBPbIqV92AaeXatLxBI9gBamrtHrhAL1wy0L2yHvtyaeHbnfgDOvwBHrxAJfwnaebbnrfifHhDYfgasaacH8akY=wiFfYdH8Gipec8Eeeu0xXdbba9frFj0=OqFfea0dXdd9vqai=hGuQ8kuc9pgc9s8qqaq=dirpe0xb9q8qiLsFr0=vr0=vr0dc8meaabaqaciaacaGaaeqabaWaaeGaeaaakeaaimaacqWFwe=vaaa@384D@^max^.

##### Partial scores

For profile matching problem, we are only given the score threshold λ for the whole structured motif. We here develop a method to compute a partial score threshold for any sub-profile, which can lead to great pruning efficiency. For a sub-profile ℳ′
 MathType@MTEF@5@5@+=feaafiart1ev1aaatCvAUfKttLearuWrP9MDH5MBPbIqV92AaeXatLxBI9gBamrtHrhAL1wy0L2yHvtyaeHbnfgDOvwBHrxAJfwnaebbnrfifHhDYfgasaacH8akY=wiFfYdH8Gipec8Eeeu0xXdbba9frFj0=OqFfea0dXdd9vqai=hGuQ8kuc9pgc9s8qqaq=dirpe0xb9q8qiLsFr0=vr0=vr0dc8meaabaqaciaacaGaaeqabaWaaeGaeaaakeaaimaacuWFZestgaqbaaaa@379C@ of profile ℳ
 MathType@MTEF@5@5@+=feaafiart1ev1aaatCvAUfKttLearuWrP9MDH5MBPbIqV92AaeXatLxBI9gBamrtHrhAL1wy0L2yHvtyaeHbnfgDOvwBHrxAJfwnaebbnrfifHhDYfgasaacH8akY=wiFfYdH8Gipec8Eeeu0xXdbba9frFj0=OqFfea0dXdd9vqai=hGuQ8kuc9pgc9s8qqaq=dirpe0xb9q8qiLsFr0=vr0=vr0dc8meaabaqaciaacaGaaeqabaWaaeGaeaaakeaaimaacqWFZestaaa@3790@, let λ^*n *^(ℳ′
 MathType@MTEF@5@5@+=feaafiart1ev1aaatCvAUfKttLearuWrP9MDH5MBPbIqV92AaeXatLxBI9gBamrtHrhAL1wy0L2yHvtyaeHbnfgDOvwBHrxAJfwnaebbnrfifHhDYfgasaacH8akY=wiFfYdH8Gipec8Eeeu0xXdbba9frFj0=OqFfea0dXdd9vqai=hGuQ8kuc9pgc9s8qqaq=dirpe0xb9q8qiLsFr0=vr0=vr0dc8meaabaqaciaacaGaaeqabaWaaeGaeaaakeaaimaacuWFZestgaqbaaaa@379C@) denote its minimum score threshold and let ℳ′
 MathType@MTEF@5@5@+=feaafiart1ev1aaatCvAUfKttLearuWrP9MDH5MBPbIqV92AaeXatLxBI9gBamrtHrhAL1wy0L2yHvtyaeHbnfgDOvwBHrxAJfwnaebbnrfifHhDYfgasaacH8akY=wiFfYdH8Gipec8Eeeu0xXdbba9frFj0=OqFfea0dXdd9vqai=hGuQ8kuc9pgc9s8qqaq=dirpe0xb9q8qiLsFr0=vr0=vr0dc8meaabaqaciaacaGaaeqabaWaaeGaeaaakeaaimaacuWFZestgaqbaaaa@379C@^max ^be its maximum weight, i.e., the sum of the maximum weights (regardless of the symbol) across all positions in ℳ′
 MathType@MTEF@5@5@+=feaafiart1ev1aaatCvAUfKttLearuWrP9MDH5MBPbIqV92AaeXatLxBI9gBamrtHrhAL1wy0L2yHvtyaeHbnfgDOvwBHrxAJfwnaebbnrfifHhDYfgasaacH8akY=wiFfYdH8Gipec8Eeeu0xXdbba9frFj0=OqFfea0dXdd9vqai=hGuQ8kuc9pgc9s8qqaq=dirpe0xb9q8qiLsFr0=vr0=vr0dc8meaabaqaciaacaGaaeqabaWaaeGaeaaakeaaimaacuWFZestgaqbaaaa@379C@. Then the minimum (partial) score threshold for ℳ′
 MathType@MTEF@5@5@+=feaafiart1ev1aaatCvAUfKttLearuWrP9MDH5MBPbIqV92AaeXatLxBI9gBamrtHrhAL1wy0L2yHvtyaeHbnfgDOvwBHrxAJfwnaebbnrfifHhDYfgasaacH8akY=wiFfYdH8Gipec8Eeeu0xXdbba9frFj0=OqFfea0dXdd9vqai=hGuQ8kuc9pgc9s8qqaq=dirpe0xb9q8qiLsFr0=vr0=vr0dc8meaabaqaciaacaGaaeqabaWaaeGaeaaakeaaimaacuWFZestgaqbaaaa@379C@ is calculated as:

λn(ℳ′)=λn−(Wmax⁡−ℳ′max⁡)     (5)
 MathType@MTEF@5@5@+=feaafiart1ev1aaatCvAUfKttLearuWrP9MDH5MBPbIqV92AaeXatLxBI9gBamrtHrhAL1wy0L2yHvtyaeHbnfgDOvwBHrxAJfwnaebbnrfifHhDYfgasaacH8akY=wiFfYdH8Gipec8Eeeu0xXdbba9frFj0=OqFfea0dXdd9vqai=hGuQ8kuc9pgc9s8qqaq=dirpe0xb9q8qiLsFr0=vr0=vr0dc8meaabaqaciaacaGaaeqabaWaaeGaeaaakeaacqaH7oaBdaahaaWcbeqaaiabd6gaUbaakiabcIcaOGWaaiqb=ntinzaafaGaeiykaKIaeyypa0Jaeq4UdW2aaWbaaSqabeaacqWGUbGBaaGccqGHsislcqGGOaakcqWFwe=vdaahaaWcbeqaaiGbc2gaTjabcggaHjabcIha4baakiabgkHiTiqb=ntinzaafaWaaWbaaSqabeaacyGGTbqBcqGGHbqycqGG4baEaaGccqGGPaqkcaWLjaGaaCzcamaabmaabaGaeGynaudacaGLOaGaayzkaaaaaa@5414@

In another word, the minimum threshold for any sub-profile ℳ′
 MathType@MTEF@5@5@+=feaafiart1ev1aaatCvAUfKttLearuWrP9MDH5MBPbIqV92AaeXatLxBI9gBamrtHrhAL1wy0L2yHvtyaeHbnfgDOvwBHrxAJfwnaebbnrfifHhDYfgasaacH8akY=wiFfYdH8Gipec8Eeeu0xXdbba9frFj0=OqFfea0dXdd9vqai=hGuQ8kuc9pgc9s8qqaq=dirpe0xb9q8qiLsFr0=vr0=vr0dc8meaabaqaciaacaGaaeqabaWaaeGaeaaakeaaimaacuWFZestgaqbaaaa@379C@ is calculated as the difference of the minimum threshold for the whole structured motif and the maximum weight of the motif excluding the sub-profile ℳ′
 MathType@MTEF@5@5@+=feaafiart1ev1aaatCvAUfKttLearuWrP9MDH5MBPbIqV92AaeXatLxBI9gBamrtHrhAL1wy0L2yHvtyaeHbnfgDOvwBHrxAJfwnaebbnrfifHhDYfgasaacH8akY=wiFfYdH8Gipec8Eeeu0xXdbba9frFj0=OqFfea0dXdd9vqai=hGuQ8kuc9pgc9s8qqaq=dirpe0xb9q8qiLsFr0=vr0=vr0dc8meaabaqaciaacaGaaeqabaWaaeGaeaaakeaaimaacuWFZestgaqbaaaa@379C@. For example, consider the weighted profile *M*_1 _[0, 5] *M*_2 _[0, 9] *M*_3 _shown in Figure 8. Assume that λ = 0.8, then using Equation 4(a), we have λ^*n *^= λ W
 MathType@MTEF@5@5@+=feaafiart1ev1aaatCvAUfKttLearuWrP9MDH5MBPbIqV92AaeXatLxBI9gBamrtHrhAL1wy0L2yHvtyaeHbnfgDOvwBHrxAJfwnaebbnrfifHhDYfgasaacH8akY=wiFfYdH8Gipec8Eeeu0xXdbba9frFj0=OqFfea0dXdd9vqai=hGuQ8kuc9pgc9s8qqaq=dirpe0xb9q8qiLsFr0=vr0=vr0dc8meaabaqaciaacaGaaeqabaWaaeGaeaaakeaaimaacqWFwe=vaaa@384D@^max ^= 0.8 × 10.75 = 8.60. Note that W
 MathType@MTEF@5@5@+=feaafiart1ev1aaatCvAUfKttLearuWrP9MDH5MBPbIqV92AaeXatLxBI9gBamrtHrhAL1wy0L2yHvtyaeHbnfgDOvwBHrxAJfwnaebbnrfifHhDYfgasaacH8akY=wiFfYdH8Gipec8Eeeu0xXdbba9frFj0=OqFfea0dXdd9vqai=hGuQ8kuc9pgc9s8qqaq=dirpe0xb9q8qiLsFr0=vr0=vr0dc8meaabaqaciaacaGaaeqabaWaaeGaeaaakeaaimaacqWFwe=vaaa@384D@^max ^= 10.75 is obtained by adding all the highest weights (in bold) at each position in the profile. Now consider the sub-profile of ℳ
 MathType@MTEF@5@5@+=feaafiart1ev1aaatCvAUfKttLearuWrP9MDH5MBPbIqV92AaeXatLxBI9gBamrtHrhAL1wy0L2yHvtyaeHbnfgDOvwBHrxAJfwnaebbnrfifHhDYfgasaacH8akY=wiFfYdH8Gipec8Eeeu0xXdbba9frFj0=OqFfea0dXdd9vqai=hGuQ8kuc9pgc9s8qqaq=dirpe0xb9q8qiLsFr0=vr0=vr0dc8meaabaqaciaacaGaaeqabaWaaeGaeaaakeaaimaacqWFZestaaa@3790@ consisting only of component *M*_3_. Then the score threshold for ℳ′
 MathType@MTEF@5@5@+=feaafiart1ev1aaatCvAUfKttLearuWrP9MDH5MBPbIqV92AaeXatLxBI9gBamrtHrhAL1wy0L2yHvtyaeHbnfgDOvwBHrxAJfwnaebbnrfifHhDYfgasaacH8akY=wiFfYdH8Gipec8Eeeu0xXdbba9frFj0=OqFfea0dXdd9vqai=hGuQ8kuc9pgc9s8qqaq=dirpe0xb9q8qiLsFr0=vr0=vr0dc8meaabaqaciaacaGaaeqabaWaaeGaeaaakeaaimaacuWFZestgaqbaaaa@379C@' = *M*_3 _is given as λ^*n *^(*M*_3_) = 8.6 - (10.75 – 3.75) = 1.60. Likewise, we can compute the minimum score threshold for any sub-profile (composed of any set of positions) of ℳ
 MathType@MTEF@5@5@+=feaafiart1ev1aaatCvAUfKttLearuWrP9MDH5MBPbIqV92AaeXatLxBI9gBamrtHrhAL1wy0L2yHvtyaeHbnfgDOvwBHrxAJfwnaebbnrfifHhDYfgasaacH8akY=wiFfYdH8Gipec8Eeeu0xXdbba9frFj0=OqFfea0dXdd9vqai=hGuQ8kuc9pgc9s8qqaq=dirpe0xb9q8qiLsFr0=vr0=vr0dc8meaabaqaciaacaGaaeqabaWaaeGaeaaakeaaimaacqWFZestaaa@3790@. Wu et al. [27] proposed a (permuted) lookahead profile scoring approach similar to our partial scoring method. However, their method is only for simple motif scoring, while our method is applied for both simple motif scoring and structured motif scoring.

##### Core scores

In many biologically relevant motifs, some positions are more conserved than others. We call them *core positions*, and these are precisely those positions with high information content. We choose the top *h *(usually 4 to 6) positions in the profile with highest information content as the core positions. For any potential match *S'*, we can compute its core score ℛ
 MathType@MTEF@5@5@+=feaafiart1ev1aaatCvAUfKttLearuWrP9MDH5MBPbIqV92AaeXatLxBI9gBamrtHrhAL1wy0L2yHvtyaeHbnfgDOvwBHrxAJfwnaebbnrfifHhDYfgasaacH8akY=wiFfYdH8Gipec8Eeeu0xXdbba9frFj0=OqFfea0dXdd9vqai=hGuQ8kuc9pgc9s8qqaq=dirpe0xb9q8qiLsFr0=vr0=vr0dc8meaabaqaciaacaGaaeqabaWaaeGaeaaakeaaimaacqWFBeIuaaa@377D@^*c *^(*S'*) to be the sum of the weights over only the core positions, and we require that ℛ
 MathType@MTEF@5@5@+=feaafiart1ev1aaatCvAUfKttLearuWrP9MDH5MBPbIqV92AaeXatLxBI9gBamrtHrhAL1wy0L2yHvtyaeHbnfgDOvwBHrxAJfwnaebbnrfifHhDYfgasaacH8akY=wiFfYdH8Gipec8Eeeu0xXdbba9frFj0=OqFfea0dXdd9vqai=hGuQ8kuc9pgc9s8qqaq=dirpe0xb9q8qiLsFr0=vr0=vr0dc8meaabaqaciaacaGaaeqabaWaaeGaeaaakeaaimaacqWFBeIuaaa@377D@^*c *^(*S'*) satisfy a user-specified normalized core score threshold λ_*c*_. Just like the score threshold, we use the normalized core score threshold λcn
 MathType@MTEF@5@5@+=feaafiart1ev1aaatCvAUfKttLearuWrP9MDH5MBPbIqV92AaeXatLxBI9gBaebbnrfifHhDYfgasaacH8akY=wiFfYdH8Gipec8Eeeu0xXdbba9frFj0=OqFfea0dXdd9vqai=hGuQ8kuc9pgc9s8qqaq=dirpe0xb9q8qiLsFr0=vr0=vr0dc8meaabaqaciaacaGaaeqabaqabeGadaaakeaacqaH7oaBdaqhaaWcbaGaem4yamgabaGaemOBa4gaaaaa@3141@ for pruning, and furthermore, we can compute the core threshold for any sub-profile of the core positions. Note that we compute separate (partial) core scores for each component. For example, assuming we restrict our attention to only component *M*_1 _in Figure 8, with λ_*c *_= 1, we have ℛ
 MathType@MTEF@5@5@+=feaafiart1ev1aaatCvAUfKttLearuWrP9MDH5MBPbIqV92AaeXatLxBI9gBamrtHrhAL1wy0L2yHvtyaeHbnfgDOvwBHrxAJfwnaebbnrfifHhDYfgasaacH8akY=wiFfYdH8Gipec8Eeeu0xXdbba9frFj0=OqFfea0dXdd9vqai=hGuQ8kuc9pgc9s8qqaq=dirpe0xb9q8qiLsFr0=vr0=vr0dc8meaabaqaciaacaGaaeqabaWaaeGaeaaakeaaimaacqWFBeIuaaa@377D@^*c *^(*M*_1_) = 1.36 + 0.78 = 2.14.

#### Motif enumeration

##### Simple motif scoring

Given a profile motif ℳ
 MathType@MTEF@5@5@+=feaafiart1ev1aaatCvAUfKttLearuWrP9MDH5MBPbIqV92AaeXatLxBI9gBamrtHrhAL1wy0L2yHvtyaeHbnfgDOvwBHrxAJfwnaebbnrfifHhDYfgasaacH8akY=wiFfYdH8Gipec8Eeeu0xXdbba9frFj0=OqFfea0dXdd9vqai=hGuQ8kuc9pgc9s8qqaq=dirpe0xb9q8qiLsFr0=vr0=vr0dc8meaabaqaciaacaGaaeqabaWaaeGaeaaakeaaimaacqWFZestaaa@3790@, score threshold λ, core score threshold λ_*c*_, and a sequence set S
 MathType@MTEF@5@5@+=feaafiart1ev1aaatCvAUfKttLearuWrP9MDH5MBPbIqV92AaeXatLxBI9gBamrtHrhAL1wy0L2yHvtyaeHbnfgDOvwBHrxAJfwnaebbnrfifHhDYfgasaacH8akY=wiFfYdH8Gipec8Eeeu0xXdbba9frFj0=OqFfea0dXdd9vqai=hGuQ8kuc9pgc9s8qqaq=dirpe0xb9q8qiLsFr0=vr0=vr0dc8meaabaqaciaacaGaaeqabaWaaeGaeaaakeaaimaacqWFse=uaaa@3845@, for each sequence *S *∈ S
 MathType@MTEF@5@5@+=feaafiart1ev1aaatCvAUfKttLearuWrP9MDH5MBPbIqV92AaeXatLxBI9gBamrtHrhAL1wy0L2yHvtyaeHbnfgDOvwBHrxAJfwnaebbnrfifHhDYfgasaacH8akY=wiFfYdH8Gipec8Eeeu0xXdbba9frFj0=OqFfea0dXdd9vqai=hGuQ8kuc9pgc9s8qqaq=dirpe0xb9q8qiLsFr0=vr0=vr0dc8meaabaqaciaacaGaaeqabaWaaeGaeaaakeaaimaacqWFse=uaaa@3845@, we first compute the potential matches for each component *M*_*i *_separately. That is, for each component *M*_*i*_, for each consecutive subsequence *S' *of length |*M*_*i*_| starting at each position *j *in *S *(i.e., *S' *= *S *[*j*, *j *+ |*M*_*i*_| - 1]) we compute its component core score ℛ
 MathType@MTEF@5@5@+=feaafiart1ev1aaatCvAUfKttLearuWrP9MDH5MBPbIqV92AaeXatLxBI9gBamrtHrhAL1wy0L2yHvtyaeHbnfgDOvwBHrxAJfwnaebbnrfifHhDYfgasaacH8akY=wiFfYdH8Gipec8Eeeu0xXdbba9frFj0=OqFfea0dXdd9vqai=hGuQ8kuc9pgc9s8qqaq=dirpe0xb9q8qiLsFr0=vr0=vr0dc8meaabaqaciaacaGaaeqabaWaaeGaeaaakeaaimaacqWFBeIuaaa@377D@^*c *^(*S'*), and partial score ℛ
 MathType@MTEF@5@5@+=feaafiart1ev1aaatCvAUfKttLearuWrP9MDH5MBPbIqV92AaeXatLxBI9gBamrtHrhAL1wy0L2yHvtyaeHbnfgDOvwBHrxAJfwnaebbnrfifHhDYfgasaacH8akY=wiFfYdH8Gipec8Eeeu0xXdbba9frFj0=OqFfea0dXdd9vqai=hGuQ8kuc9pgc9s8qqaq=dirpe0xb9q8qiLsFr0=vr0=vr0dc8meaabaqaciaacaGaaeqabaWaaeGaeaaakeaaimaacqWFBeIuaaa@377D@ (*S'*). If these scores are larger than the corresponding score thresholds λcn
 MathType@MTEF@5@5@+=feaafiart1ev1aaatCvAUfKttLearuWrP9MDH5MBPbIqV92AaeXatLxBI9gBaebbnrfifHhDYfgasaacH8akY=wiFfYdH8Gipec8Eeeu0xXdbba9frFj0=OqFfea0dXdd9vqai=hGuQ8kuc9pgc9s8qqaq=dirpe0xb9q8qiLsFr0=vr0=vr0dc8meaabaqaciaacaGaaeqabaqabeGadaaakeaacqaH7oaBdaqhaaWcbaGaem4yamgabaGaemOBa4gaaaaa@3141@(*M*_*i*_) and λ^*n *^(*M*_*i*_), respectively, we record this position *j *into the component's pos-list P
 MathType@MTEF@5@5@+=feaafiart1ev1aaatCvAUfKttLearuWrP9MDH5MBPbIqV92AaeXatLxBI9gBamrtHrhAL1wy0L2yHvtyaeHbnfgDOvwBHrxAJfwnaebbnrfifHhDYfgasaacH8akY=wiFfYdH8Gipec8Eeeu0xXdbba9frFj0=OqFfea0dXdd9vqai=hGuQ8kuc9pgc9s8qqaq=dirpe0xb9q8qiLsFr0=vr0=vr0dc8meaabaqaciaacaGaaeqabaWaaeGaeaaakeaacqqGqbauaaa@3786@(*M*_*i*_).

To prune matches *S' *that will eventually not meet the score threshold, we check the score threshold as each position in *S' *is being considered. If the score for any prefix of *S' *falls below the score threshold, we can discard *S'*. In fact, before applying scores over all positions, we first consider the scores for the prefixes of the core positions within the component. This continuous check for the core positions and regular positions leads to very effective pruning. Note that as opposed to previous methods [19-22], our approach does not require the core positions be consecutive so as to find the most conserved parts in the profile.

**Figure 9:**
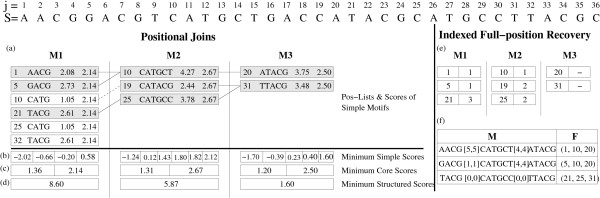
**Profile Positional Joins and Full Position Recovery**. The example sequence *S *has length 36. (a) Under each component *M*_*i*_, 1 ≤ *i *≤ 3 a set of tuples are listed in the format (start position, subsequence, score, core score). (b) Minimum Simple Scores row gives the minimum score threshold for any prefix of a component; (c) Minimum Core Scores gives the minimum core score threshold for any prefix of core positions for each component; (d) Minimum Structured Scores gives the minimum score threshold for each (component-wise) suffix of the structured motif; (e) The final pos-lists and the index for each component; (f) The motifs (M) that satisfy the profile, along with their full positions (F).

Figure 9 shows how we search for the example profile motif from Figure 8, namely *M*_1 _[0, 5] *M*_2 _[0, 9] *M*_3_. In Figure 9(a), under each component *M*_*i*_, 1 ≤ *i *≤ 3 a set of tuples are listed in the format (*j*, *S'*, ℛ
 MathType@MTEF@5@5@+=feaafiart1ev1aaatCvAUfKttLearuWrP9MDH5MBPbIqV92AaeXatLxBI9gBamrtHrhAL1wy0L2yHvtyaeHbnfgDOvwBHrxAJfwnaebbnrfifHhDYfgasaacH8akY=wiFfYdH8Gipec8Eeeu0xXdbba9frFj0=OqFfea0dXdd9vqai=hGuQ8kuc9pgc9s8qqaq=dirpe0xb9q8qiLsFr0=vr0=vr0dc8meaabaqaciaacaGaaeqabaWaaeGaeaaakeaaimaacqWFBeIuaaa@377D@(*S'*), ℛ
 MathType@MTEF@5@5@+=feaafiart1ev1aaatCvAUfKttLearuWrP9MDH5MBPbIqV92AaeXatLxBI9gBamrtHrhAL1wy0L2yHvtyaeHbnfgDOvwBHrxAJfwnaebbnrfifHhDYfgasaacH8akY=wiFfYdH8Gipec8Eeeu0xXdbba9frFj0=OqFfea0dXdd9vqai=hGuQ8kuc9pgc9s8qqaq=dirpe0xb9q8qiLsFr0=vr0=vr0dc8meaabaqaciaacaGaaeqabaWaaeGaeaaakeaaimaacqWFBeIuaaa@377D@^*C *^(*S'*)), where *j *is a position in the given sequence *S*, *S' *is the subsequence *S *[*j*, *j *+ |*M*_*i*_| - 1], ℛ
 MathType@MTEF@5@5@+=feaafiart1ev1aaatCvAUfKttLearuWrP9MDH5MBPbIqV92AaeXatLxBI9gBamrtHrhAL1wy0L2yHvtyaeHbnfgDOvwBHrxAJfwnaebbnrfifHhDYfgasaacH8akY=wiFfYdH8Gipec8Eeeu0xXdbba9frFj0=OqFfea0dXdd9vqai=hGuQ8kuc9pgc9s8qqaq=dirpe0xb9q8qiLsFr0=vr0=vr0dc8meaabaqaciaacaGaaeqabaWaaeGaeaaakeaaimaacqWFBeIuaaa@377D@ (*S'*) is the profile score for *S'*, and ℛ
 MathType@MTEF@5@5@+=feaafiart1ev1aaatCvAUfKttLearuWrP9MDH5MBPbIqV92AaeXatLxBI9gBamrtHrhAL1wy0L2yHvtyaeHbnfgDOvwBHrxAJfwnaebbnrfifHhDYfgasaacH8akY=wiFfYdH8Gipec8Eeeu0xXdbba9frFj0=OqFfea0dXdd9vqai=hGuQ8kuc9pgc9s8qqaq=dirpe0xb9q8qiLsFr0=vr0=vr0dc8meaabaqaciaacaGaaeqabaWaaeGaeaaakeaaimaacqWFBeIuaaa@377D@^*c *^(*S'*) is the core score for *S'*. In this example we use the core score threshold λ_*c *_= 1 and the score threshold λ = 0.8. We use Equation 4(a) for normalization. In Figure 9(b)–(c) the minimum thresholds are given: (b) gives the score thresholds for each prefix of a given component. For example, looking at the 1st position of *M*_2_, we have the score threshold λ^*n *^(*M*_2 _[1]) = 8.60 - (10.75 - 0.91) = -1.24 (using Equation 5). (c) gives the core thresholds for each prefix over core positions in each component. For example, for the 1st core position of *M*_1_, we have λcn
 MathType@MTEF@5@5@+=feaafiart1ev1aaatCvAUfKttLearuWrP9MDH5MBPbIqV92AaeXatLxBI9gBaebbnrfifHhDYfgasaacH8akY=wiFfYdH8Gipec8Eeeu0xXdbba9frFj0=OqFfea0dXdd9vqai=hGuQ8kuc9pgc9s8qqaq=dirpe0xb9q8qiLsFr0=vr0=vr0dc8meaabaqaciaacaGaaeqabaqabeGadaaakeaacqaH7oaBdaqhaaWcbaGaem4yamgabaGaemOBa4gaaaaa@3141@ (*M*_1 _[2]) = 2.14 - (2.14 - 1.36) = 1.36. Consider component *M*_1_, whose core positions are 2 and 4, and consider the subsequence *S' *= *AACG *starting at position *j *= 1 in *S*. We first check position 2 and get its core score ℛ
 MathType@MTEF@5@5@+=feaafiart1ev1aaatCvAUfKttLearuWrP9MDH5MBPbIqV92AaeXatLxBI9gBamrtHrhAL1wy0L2yHvtyaeHbnfgDOvwBHrxAJfwnaebbnrfifHhDYfgasaacH8akY=wiFfYdH8Gipec8Eeeu0xXdbba9frFj0=OqFfea0dXdd9vqai=hGuQ8kuc9pgc9s8qqaq=dirpe0xb9q8qiLsFr0=vr0=vr0dc8meaabaqaciaacaGaaeqabaWaaeGaeaaakeaaimaacqWFBeIuaaa@377D@^*c *^(*S'*) = W
 MathType@MTEF@5@5@+=feaafiart1ev1aaatCvAUfKttLearuWrP9MDH5MBPbIqV92AaeXatLxBI9gBamrtHrhAL1wy0L2yHvtyaeHbnfgDOvwBHrxAJfwnaebbnrfifHhDYfgasaacH8akY=wiFfYdH8Gipec8Eeeu0xXdbba9frFj0=OqFfea0dXdd9vqai=hGuQ8kuc9pgc9s8qqaq=dirpe0xb9q8qiLsFr0=vr0=vr0dc8meaabaqaciaacaGaaeqabaWaaeGaeaaakeaaimaacqWFwe=vaaa@384D@_*A*2 _= 1.36, which indeed passes the corresponding threshold λcn
 MathType@MTEF@5@5@+=feaafiart1ev1aaatCvAUfKttLearuWrP9MDH5MBPbIqV92AaeXatLxBI9gBaebbnrfifHhDYfgasaacH8akY=wiFfYdH8Gipec8Eeeu0xXdbba9frFj0=OqFfea0dXdd9vqai=hGuQ8kuc9pgc9s8qqaq=dirpe0xb9q8qiLsFr0=vr0=vr0dc8meaabaqaciaacaGaaeqabaqabeGadaaakeaacqaH7oaBdaqhaaWcbaGaem4yamgabaGaemOBa4gaaaaa@3141@ = 1.36. Thus we continue checking position 4 and get ℛ
 MathType@MTEF@5@5@+=feaafiart1ev1aaatCvAUfKttLearuWrP9MDH5MBPbIqV92AaeXatLxBI9gBamrtHrhAL1wy0L2yHvtyaeHbnfgDOvwBHrxAJfwnaebbnrfifHhDYfgasaacH8akY=wiFfYdH8Gipec8Eeeu0xXdbba9frFj0=OqFfea0dXdd9vqai=hGuQ8kuc9pgc9s8qqaq=dirpe0xb9q8qiLsFr0=vr0=vr0dc8meaabaqaciaacaGaaeqabaWaaeGaeaaakeaaimaacqWFBeIuaaa@377D@^*c *^(*S'*) = W
 MathType@MTEF@5@5@+=feaafiart1ev1aaatCvAUfKttLearuWrP9MDH5MBPbIqV92AaeXatLxBI9gBamrtHrhAL1wy0L2yHvtyaeHbnfgDOvwBHrxAJfwnaebbnrfifHhDYfgasaacH8akY=wiFfYdH8Gipec8Eeeu0xXdbba9frFj0=OqFfea0dXdd9vqai=hGuQ8kuc9pgc9s8qqaq=dirpe0xb9q8qiLsFr0=vr0=vr0dc8meaabaqaciaacaGaaeqabaWaaeGaeaaakeaaimaacqWFwe=vaaa@384D@_*A*2 _+ W
 MathType@MTEF@5@5@+=feaafiart1ev1aaatCvAUfKttLearuWrP9MDH5MBPbIqV92AaeXatLxBI9gBamrtHrhAL1wy0L2yHvtyaeHbnfgDOvwBHrxAJfwnaebbnrfifHhDYfgasaacH8akY=wiFfYdH8Gipec8Eeeu0xXdbba9frFj0=OqFfea0dXdd9vqai=hGuQ8kuc9pgc9s8qqaq=dirpe0xb9q8qiLsFr0=vr0=vr0dc8meaabaqaciaacaGaaeqabaWaaeGaeaaakeaaimaacqWFwe=vaaa@384D@_*G*4 _= 2.14 ≥ λcn
 MathType@MTEF@5@5@+=feaafiart1ev1aaatCvAUfKttLearuWrP9MDH5MBPbIqV92AaeXatLxBI9gBaebbnrfifHhDYfgasaacH8akY=wiFfYdH8Gipec8Eeeu0xXdbba9frFj0=OqFfea0dXdd9vqai=hGuQ8kuc9pgc9s8qqaq=dirpe0xb9q8qiLsFr0=vr0=vr0dc8meaabaqaciaacaGaaeqabaqabeGadaaakeaacqaH7oaBdaqhaaWcbaGaem4yamgabaGaemOBa4gaaaaa@3141@ = 2.14. Thus we continue to check the whole score. Checking for position 1 gives W
 MathType@MTEF@5@5@+=feaafiart1ev1aaatCvAUfKttLearuWrP9MDH5MBPbIqV92AaeXatLxBI9gBamrtHrhAL1wy0L2yHvtyaeHbnfgDOvwBHrxAJfwnaebbnrfifHhDYfgasaacH8akY=wiFfYdH8Gipec8Eeeu0xXdbba9frFj0=OqFfea0dXdd9vqai=hGuQ8kuc9pgc9s8qqaq=dirpe0xb9q8qiLsFr0=vr0=vr0dc8meaabaqaciaacaGaaeqabaWaaeGaeaaakeaaimaacqWFwe=vaaa@384D@_*A*1 _= -0.53 > -2.02; then a check for the prefix up to position 2 gives W
 MathType@MTEF@5@5@+=feaafiart1ev1aaatCvAUfKttLearuWrP9MDH5MBPbIqV92AaeXatLxBI9gBamrtHrhAL1wy0L2yHvtyaeHbnfgDOvwBHrxAJfwnaebbnrfifHhDYfgasaacH8akY=wiFfYdH8Gipec8Eeeu0xXdbba9frFj0=OqFfea0dXdd9vqai=hGuQ8kuc9pgc9s8qqaq=dirpe0xb9q8qiLsFr0=vr0=vr0dc8meaabaqaciaacaGaaeqabaWaaeGaeaaakeaaimaacqWFwe=vaaa@384D@_*A*1 _+ W
 MathType@MTEF@5@5@+=feaafiart1ev1aaatCvAUfKttLearuWrP9MDH5MBPbIqV92AaeXatLxBI9gBamrtHrhAL1wy0L2yHvtyaeHbnfgDOvwBHrxAJfwnaebbnrfifHhDYfgasaacH8akY=wiFfYdH8Gipec8Eeeu0xXdbba9frFj0=OqFfea0dXdd9vqai=hGuQ8kuc9pgc9s8qqaq=dirpe0xb9q8qiLsFr0=vr0=vr0dc8meaabaqaciaacaGaaeqabaWaaeGaeaaakeaaimaacqWFwe=vaaa@384D@_*A*2 _= 0.83 > -0.66. By continuing this process, we see that AACG satisfies the core score threshold as well as the score threshold. Figure 9(a) shows all the matching positions, i.e., the pos-list, for each component in the profile.

##### Structured motif scoring and positional joins

After obtaining the pos-lists of simple motif components, we can enumerate structured motifs by doing positional joins on these pos-lists, as already outlined for structured pattern search. We compute the positional joins with *M*_*i *_as the head and *M*_*i*+1 _⋯ *M*_*k *_as the tail, as we start from *M*_*k *_as the head and end at *M*_1 _as the head. During the positional joins we also check the partial structured score thresholds λ^*n *^(*M*_*i *_⋯ *M*_*k*_). If the check fails at any stage we prune the match candidate. We keep a pattern (along with its score) only if it satisfies the full structured score threshold λ^*n*^.

Figure 9(a) shows how to enumerate structured motifs via positional joins. The pos-list of each component is simply the set of positions (1st element of the quadruples) under it. For example, P
 MathType@MTEF@5@5@+=feaafiart1ev1aaatCvAUfKttLearuWrP9MDH5MBPbIqV92AaeXatLxBI9gBamrtHrhAL1wy0L2yHvtyaeHbnfgDOvwBHrxAJfwnaebbnrfifHhDYfgasaacH8akY=wiFfYdH8Gipec8Eeeu0xXdbba9frFj0=OqFfea0dXdd9vqai=hGuQ8kuc9pgc9s8qqaq=dirpe0xb9q8qiLsFr0=vr0=vr0dc8meaabaqaciaacaGaaeqabaWaaeGaeaaakeaacqqGqbauaaa@3786@(*M*_1_) = {1, 5, 10, 21, 25, 32}. As before the joins proceed from *M*_3 _to *M*_1_, i.e., first we obtain the pos-list for *M*_2 _[0,9] *M*_3_, and then for *M*_1 _[0, 5] as head and *M*_2 _[0, 9] *M*_3 _as the tail. At any stage, the head motif's pos-list corresponds to the full list of positions shown, whereas the tail's pos-list consists only of the shaded positions. For illustration, we add a link between any two positions, *x *and *y*, in adjacent columns if their difference (*d *= *y *- *x *- 1) falls within the corresponding gap range. If the current partial motif starting at position *x *also satisfies the corresponding (partial) score threshold, the link is solid; otherwise, the link is dashed. When joining *M*_2 _as the head and *M*_3 _as the tail with a gap of [0, 9], the positions *x *∈ P
 MathType@MTEF@5@5@+=feaafiart1ev1aaatCvAUfKttLearuWrP9MDH5MBPbIqV92AaeXatLxBI9gBamrtHrhAL1wy0L2yHvtyaeHbnfgDOvwBHrxAJfwnaebbnrfifHhDYfgasaacH8akY=wiFfYdH8Gipec8Eeeu0xXdbba9frFj0=OqFfea0dXdd9vqai=hGuQ8kuc9pgc9s8qqaq=dirpe0xb9q8qiLsFr0=vr0=vr0dc8meaabaqaciaacaGaaeqabaWaaeGaeaaakeaacqqGqbauaaa@3786@(*M*_2_) that satisfy the gap constraint are 10, 19 and 25 (marked in bold), which thus form the pos-list of *M*_2 _[0, 9] *M*_3_. We then check whether each occurrence satisfies the corresponding structured score threshold. Figure 9(d) shows the minimum partial scores required for each component suffix. For example the threshold λ^*n *^(*M*_2 _[0, 9] *M*_3_) = 5.87. Checking the score for CATACG[0,9]TTACG, we get 2.44 + 3.48 = 5.92 > 5.87, so we keep it. The other occurrences also satisfy the partial score threshold. Next we join P
 MathType@MTEF@5@5@+=feaafiart1ev1aaatCvAUfKttLearuWrP9MDH5MBPbIqV92AaeXatLxBI9gBamrtHrhAL1wy0L2yHvtyaeHbnfgDOvwBHrxAJfwnaebbnrfifHhDYfgasaacH8akY=wiFfYdH8Gipec8Eeeu0xXdbba9frFj0=OqFfea0dXdd9vqai=hGuQ8kuc9pgc9s8qqaq=dirpe0xb9q8qiLsFr0=vr0=vr0dc8meaabaqaciaacaGaaeqabaWaaeGaeaaakeaacqqGqbauaaa@3786@(*M*_1_) with P
 MathType@MTEF@5@5@+=feaafiart1ev1aaatCvAUfKttLearuWrP9MDH5MBPbIqV92AaeXatLxBI9gBamrtHrhAL1wy0L2yHvtyaeHbnfgDOvwBHrxAJfwnaebbnrfifHhDYfgasaacH8akY=wiFfYdH8Gipec8Eeeu0xXdbba9frFj0=OqFfea0dXdd9vqai=hGuQ8kuc9pgc9s8qqaq=dirpe0xb9q8qiLsFr0=vr0=vr0dc8meaabaqaciaacaGaaeqabaWaaeGaeaaakeaacqqGqbauaaa@3786@(*M*_2 _[0, 9] *M*_3_) to get the valid positions 1, 5, 10 and 21. When checking with the score threshold, we find that the score of CATG[0,5]CATACG[0,9]TTACG is 1.05 + 5.87 = 6.97 < 8.60 = λ^*n*^, so we discard this motif (as a result the corresponding link between the positions is dashed.) Finally, we get P
 MathType@MTEF@5@5@+=feaafiart1ev1aaatCvAUfKttLearuWrP9MDH5MBPbIqV92AaeXatLxBI9gBamrtHrhAL1wy0L2yHvtyaeHbnfgDOvwBHrxAJfwnaebbnrfifHhDYfgasaacH8akY=wiFfYdH8Gipec8Eeeu0xXdbba9frFj0=OqFfea0dXdd9vqai=hGuQ8kuc9pgc9s8qqaq=dirpe0xb9q8qiLsFr0=vr0=vr0dc8meaabaqaciaacaGaaeqabaWaaeGaeaaakeaacqqGqbauaaa@3786@(*M*_1 _[0, 5] *M*_2 _[0, 9] *M*_3_) = {1, 5, 21} as the pos-list for the full structured motif.

##### Full position recovery

Once the pos-list for the profile ℳ
 MathType@MTEF@5@5@+=feaafiart1ev1aaatCvAUfKttLearuWrP9MDH5MBPbIqV92AaeXatLxBI9gBamrtHrhAL1wy0L2yHvtyaeHbnfgDOvwBHrxAJfwnaebbnrfifHhDYfgasaacH8akY=wiFfYdH8Gipec8Eeeu0xXdbba9frFj0=OqFfea0dXdd9vqai=hGuQ8kuc9pgc9s8qqaq=dirpe0xb9q8qiLsFr0=vr0=vr0dc8meaabaqaciaacaGaaeqabaWaaeGaeaaakeaaimaacqWFZestaaa@3790@ has been computed, we can then recover the full positions from each start position, using the approach already outlined for the structured pattern search, i.e., we use an index N
 MathType@MTEF@5@5@+=feaafiart1ev1aaatCvAUfKttLearuWrP9MDH5MBPbIqV92AaeXatLxBI9gBamrtHrhAL1wy0L2yHvtyaeHbnfgDOvwBHrxAJfwnaebbnrfifHhDYfgasaacH8akY=wiFfYdH8Gipec8Eeeu0xXdbba9frFj0=OqFfea0dXdd9vqai=hGuQ8kuc9pgc9s8qqaq=dirpe0xb9q8qiLsFr0=vr0=vr0dc8meaabaqaciaacaGaaeqabaWaaeGaeaaakeaaimaacqWFneVtaaa@383B@_*i *_for each component to speed up the full position recovery. For example, in Figure 9(e), to recover the full position for the occurrence starting at position 1, we follow index 1 to get position 10 in *M*_2_'s pos-list, to obtain ℱ
 MathType@MTEF@5@5@+=feaafiart1ev1aaatCvAUfKttLearuWrP9MDH5MBPbIqV92AaeXatLxBI9gBamrtHrhAL1wy0L2yHvtyaeHbnfgDOvwBHrxAJfwnaebbnrfifHhDYfgasaacH8akY=wiFfYdH8Gipec8Eeeu0xXdbba9frFj0=OqFfea0dXdd9vqai=hGuQ8kuc9pgc9s8qqaq=dirpe0xb9q8qiLsFr0=vr0=vr0dc8meaabaqaciaacaGaaeqabaWaaeGaeaaakeaaimaacqWFXeIraaa@3787@ = {(1, 10)}. Then we follow index 1 to position 20 in *M*_3_'s pos-list, to get ℱ
 MathType@MTEF@5@5@+=feaafiart1ev1aaatCvAUfKttLearuWrP9MDH5MBPbIqV92AaeXatLxBI9gBamrtHrhAL1wy0L2yHvtyaeHbnfgDOvwBHrxAJfwnaebbnrfifHhDYfgasaacH8akY=wiFfYdH8Gipec8Eeeu0xXdbba9frFj0=OqFfea0dXdd9vqai=hGuQ8kuc9pgc9s8qqaq=dirpe0xb9q8qiLsFr0=vr0=vr0dc8meaabaqaciaacaGaaeqabaWaaeGaeaaakeaaimaacqWFXeIraaa@3787@ = {(1,10, 20)} as a full position and its corresponding sequence is AACG[5]CATGCT[4]ATACG. By continuing this process, we can get the other two full positions, as shown in Figure 9(f).

### The complete SMOTIF algorithm: complexity analysis

**Figure 10:**
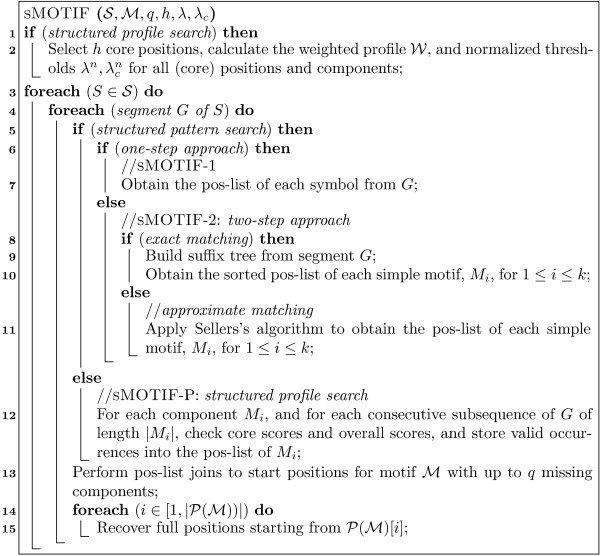
SMOTIF Algorithm.

The pseudo-code for the complete SMOTIF algorithm is shown in Figure 10. We distinguish three cases: the direct (or one-step) approach, and the two-step approach for structured pattern search are denoted as SMOTIF-1, and SMOTIF-2, respectively. The approach for structured profile search is called SMOTIF-P. Let n=∑S∈S|S|
 MathType@MTEF@5@5@+=feaafiart1ev1aaatCvAUfKttLearuWrP9MDH5MBPbIqV92AaeXatLxBI9gBamrtHrhAL1wy0L2yHvtyaeHbnfgDOvwBHrxAJfwnaebbnrfifHhDYfgasaacH8akY=wiFfYdH8Gipec8Eeeu0xXdbba9frFj0=OqFfea0dXdd9vqai=hGuQ8kuc9pgc9s8qqaq=dirpe0xb9q8qiLsFr0=vr0=vr0dc8meaabaqaciaacaGaaeqabaWaaeGaeaaakeaacqWGUbGBcqGH9aqpdaaeqaqaaiabcYha8jabdofatjabcYha8bWcbaGaem4uamLaeyicI4mcdaGae8NeXpfabeqdcqGHris5aaaa@4375@ be the total length over all sequences in S
 MathType@MTEF@5@5@+=feaafiart1ev1aaatCvAUfKttLearuWrP9MDH5MBPbIqV92AaeXatLxBI9gBamrtHrhAL1wy0L2yHvtyaeHbnfgDOvwBHrxAJfwnaebbnrfifHhDYfgasaacH8akY=wiFfYdH8Gipec8Eeeu0xXdbba9frFj0=OqFfea0dXdd9vqai=hGuQ8kuc9pgc9s8qqaq=dirpe0xb9q8qiLsFr0=vr0=vr0dc8meaabaqaciaacaGaaeqabaWaaeGaeaaakeaaimaacqWFse=uaaa@3845@, let and *m *= max_*i *_{|*M*_*i*_|} be the maximum component length, and assume that all patterns/profiles are over Σ_DNA_. In Figure 10, line 2 takes *O *(|ℳ
 MathType@MTEF@5@5@+=feaafiart1ev1aaatCvAUfKttLearuWrP9MDH5MBPbIqV92AaeXatLxBI9gBamrtHrhAL1wy0L2yHvtyaeHbnfgDOvwBHrxAJfwnaebbnrfifHhDYfgasaacH8akY=wiFfYdH8Gipec8Eeeu0xXdbba9frFj0=OqFfea0dXdd9vqai=hGuQ8kuc9pgc9s8qqaq=dirpe0xb9q8qiLsFr0=vr0=vr0dc8meaabaqaciaacaGaaeqabaWaaeGaeaaakeaaimaacqWFZestaaa@3790@| · |Σ_DNA_|) time and space to compute the weighted profile and *O *(|ℳ
 MathType@MTEF@5@5@+=feaafiart1ev1aaatCvAUfKttLearuWrP9MDH5MBPbIqV92AaeXatLxBI9gBamrtHrhAL1wy0L2yHvtyaeHbnfgDOvwBHrxAJfwnaebbnrfifHhDYfgasaacH8akY=wiFfYdH8Gipec8Eeeu0xXdbba9frFj0=OqFfea0dXdd9vqai=hGuQ8kuc9pgc9s8qqaq=dirpe0xb9q8qiLsFr0=vr0=vr0dc8meaabaqaciaacaGaaeqabaWaaeGaeaaakeaaimaacqWFZestaaa@3790@|) time/space to compute the (core) score thresholds. Line 4 reads one segment each time and takes *O *(*n*) time and space over all sequences. Line 7 also takes *O *(*n*) time and space since SMOTIF-1 scans the input sequences only once to create the pos-lists. For SMOTIF-2 with exact matching, line 9 builds the suffix tree in *O *(*n*) time and space. In line 10, to get the occurrences for all components, takes time *O *(*km*) to search in the suffix tree, and in the worst case, *O *(*n*) time/space to extract all the occurrences. To sort the occurrences takes *O *(*n *log *n*) using comparison-based sorting, or *O *(*n*) time using counting sort, for example. The total time is then *O *(*km *+ *n *log *n*) (or *O *(*km *+ *n*) if using counting sort). For SMOTIF-2 with approximate matching, line 11 applies Sellers' dynamic programming algorithm [26] which takes *O *(|ℳ
 MathType@MTEF@5@5@+=feaafiart1ev1aaatCvAUfKttLearuWrP9MDH5MBPbIqV92AaeXatLxBI9gBamrtHrhAL1wy0L2yHvtyaeHbnfgDOvwBHrxAJfwnaebbnrfifHhDYfgasaacH8akY=wiFfYdH8Gipec8Eeeu0xXdbba9frFj0=OqFfea0dXdd9vqai=hGuQ8kuc9pgc9s8qqaq=dirpe0xb9q8qiLsFr0=vr0=vr0dc8meaabaqaciaacaGaaeqabaWaaeGaeaaakeaaimaacqWFZestaaa@3790@| · *n*) time/space over all components. For SMOTIF-P, line 12 considers *O *(*n*) starting positions and the scoring takes total time *O *(|ℳ
 MathType@MTEF@5@5@+=feaafiart1ev1aaatCvAUfKttLearuWrP9MDH5MBPbIqV92AaeXatLxBI9gBamrtHrhAL1wy0L2yHvtyaeHbnfgDOvwBHrxAJfwnaebbnrfifHhDYfgasaacH8akY=wiFfYdH8Gipec8Eeeu0xXdbba9frFj0=OqFfea0dXdd9vqai=hGuQ8kuc9pgc9s8qqaq=dirpe0xb9q8qiLsFr0=vr0=vr0dc8meaabaqaciaacaGaaeqabaWaaeGaeaaakeaaimaacqWFZestaaa@3790@| · *n*) over all components. Line 13 enumerates all the possible sub-motifs of ℳ
 MathType@MTEF@5@5@+=feaafiart1ev1aaatCvAUfKttLearuWrP9MDH5MBPbIqV92AaeXatLxBI9gBamrtHrhAL1wy0L2yHvtyaeHbnfgDOvwBHrxAJfwnaebbnrfifHhDYfgasaacH8akY=wiFfYdH8Gipec8Eeeu0xXdbba9frFj0=OqFfea0dXdd9vqai=hGuQ8kuc9pgc9s8qqaq=dirpe0xb9q8qiLsFr0=vr0=vr0dc8meaabaqaciaacaGaaeqabaWaaeGaeaaakeaaimaacqWFZestaaa@3790@ with *q *missing components. The number of sub-motifs to be searched for is T=∑i=k−qk(ki)
 MathType@MTEF@5@5@+=feaafiart1ev1aaatCvAUfKttLearuWrP9MDH5MBPbIqV92AaeXatLxBI9gBaebbnrfifHhDYfgasaacH8akY=wiFfYdH8Gipec8Eeeu0xXdbba9frFj0=OqFfea0dXdd9vqai=hGuQ8kuc9pgc9s8qqaq=dirpe0xb9q8qiLsFr0=vr0=vr0dc8meaabaqaciaacaGaaeqabaqabeGadaaakeaacqWGubavcqGH9aqpdaaeWaqaamaabmaabaqbaeqabiqaaaqaaiabdUgaRbqaaiabdMgaPbaaaiaawIcacaGLPaaaaSqaaiabdMgaPjabg2da9iabdUgaRjabgkHiTiabdghaXbqaaiabdUgaRbqdcqGHris5aaaa@3CAC@ with *i *∈ [*k *- *q*, *k*] simple motifs, which in the worst case is *T *= 2^*k *^- 1 (when *q *= *k *- 1). Since the positional joins take linear time in the length of the pos-lists (the length is *O *(*n*) in the worst case), the time for pos-list joins is *O *(|ℳ
 MathType@MTEF@5@5@+=feaafiart1ev1aaatCvAUfKttLearuWrP9MDH5MBPbIqV92AaeXatLxBI9gBamrtHrhAL1wy0L2yHvtyaeHbnfgDOvwBHrxAJfwnaebbnrfifHhDYfgasaacH8akY=wiFfYdH8Gipec8Eeeu0xXdbba9frFj0=OqFfea0dXdd9vqai=hGuQ8kuc9pgc9s8qqaq=dirpe0xb9q8qiLsFr0=vr0=vr0dc8meaabaqaciaacaGaaeqabaWaaeGaeaaakeaaimaacqWFZestaaa@3790@| · *n*) for SMOTIF-1, and *O *(*kn*) for SMOTIF-2 and SMOTIF-P, when there are no missing components; these times have to be multiplied by *T *when there are *q *missing components. The number of final occurrences of the structured motif in the sequences is given as O
 MathType@MTEF@5@5@+=feaafiart1ev1aaatCvAUfKttLearuWrP9MDH5MBPbIqV92AaeXatLxBI9gBamrtHrhAL1wy0L2yHvtyaeHbnfgDOvwBHrxAJfwnaebbnrfifHhDYfgasaacH8akY=wiFfYdH8Gipec8Eeeu0xXdbba9frFj0=OqFfea0dXdd9vqai=hGuQ8kuc9pgc9s8qqaq=dirpe0xb9q8qiLsFr0=vr0=vr0dc8meaabaqaciaacaGaaeqabaWaaeGaeaaakeaaimaacqWFoe=taaa@383D@. Lines 14–15 scan a region of span *L *from each starting position, taking *O *(*L *· |O
 MathType@MTEF@5@5@+=feaafiart1ev1aaatCvAUfKttLearuWrP9MDH5MBPbIqV92AaeXatLxBI9gBamrtHrhAL1wy0L2yHvtyaeHbnfgDOvwBHrxAJfwnaebbnrfifHhDYfgasaacH8akY=wiFfYdH8Gipec8Eeeu0xXdbba9frFj0=OqFfea0dXdd9vqai=hGuQ8kuc9pgc9s8qqaq=dirpe0xb9q8qiLsFr0=vr0=vr0dc8meaabaqaciaacaGaaeqabaWaaeGaeaaakeaaimaacqWFoe=taaa@383D@|) time over all occurrences. Note that in the worst case, for any sequence *S *∈ S
 MathType@MTEF@5@5@+=feaafiart1ev1aaatCvAUfKttLearuWrP9MDH5MBPbIqV92AaeXatLxBI9gBamrtHrhAL1wy0L2yHvtyaeHbnfgDOvwBHrxAJfwnaebbnrfifHhDYfgasaacH8akY=wiFfYdH8Gipec8Eeeu0xXdbba9frFj0=OqFfea0dXdd9vqai=hGuQ8kuc9pgc9s8qqaq=dirpe0xb9q8qiLsFr0=vr0=vr0dc8meaabaqaciaacaGaaeqabaWaaeGaeaaakeaaimaacqWFse=uaaa@3845@, the number of full occurrences of the motif can be |O
 MathType@MTEF@5@5@+=feaafiart1ev1aaatCvAUfKttLearuWrP9MDH5MBPbIqV92AaeXatLxBI9gBamrtHrhAL1wy0L2yHvtyaeHbnfgDOvwBHrxAJfwnaebbnrfifHhDYfgasaacH8akY=wiFfYdH8Gipec8Eeeu0xXdbba9frFj0=OqFfea0dXdd9vqai=hGuQ8kuc9pgc9s8qqaq=dirpe0xb9q8qiLsFr0=vr0=vr0dc8meaabaqaciaacaGaaeqabaWaaeGaeaaakeaaimaacqWFoe=taaa@383D@| = O(∏i=1k−1(ui−li+1))
MathType@MTEF@5@5@+=feaafiart1ev1aaatCvAUfKttLearuWrP9MDH5MBPbIqV92AaeXatLxBI9gBaebbnrfifHhDYfgasaacH8akY=wiFfYdH8Gipec8Eeeu0xXdbba9frFj0=OqFfea0dXdd9vqai=hGuQ8kuc9pgc9s8qqaq=dirpe0xb9q8qiLsFr0=vr0=vr0dc8meaabaqaciaacaGaaeqabaqabeGadaaakeaacqWGpbWtcqGGOaakdaqeWaqaaiabcIcaOiabdwha1naaBaaaleaacqWGPbqAaeqaaOGaeyOeI0IaemiBaW2aaSbaaSqaaiabdMgaPbqabaGccqGHRaWkcqaIXaqmcqGGPaqkcqGGPaqkaSqaaiabdMgaPjabg2da9iabigdaXaqaaiabdUgaRjabgkHiTiabigdaXaqdcqGHpis1aaaa@426A@. Overall, the total time for SMOTIF is *O *(|ℳ
 MathType@MTEF@5@5@+=feaafiart1ev1aaatCvAUfKttLearuWrP9MDH5MBPbIqV92AaeXatLxBI9gBamrtHrhAL1wy0L2yHvtyaeHbnfgDOvwBHrxAJfwnaebbnrfifHhDYfgasaacH8akY=wiFfYdH8Gipec8Eeeu0xXdbba9frFj0=OqFfea0dXdd9vqai=hGuQ8kuc9pgc9s8qqaq=dirpe0xb9q8qiLsFr0=vr0=vr0dc8meaabaqaciaacaGaaeqabaWaaeGaeaaakeaaimaacqWFZestaaa@3790@| · |Σ_DNA_| + *n *· (|ℳ
 MathType@MTEF@5@5@+=feaafiart1ev1aaatCvAUfKttLearuWrP9MDH5MBPbIqV92AaeXatLxBI9gBamrtHrhAL1wy0L2yHvtyaeHbnfgDOvwBHrxAJfwnaebbnrfifHhDYfgasaacH8akY=wiFfYdH8Gipec8Eeeu0xXdbba9frFj0=OqFfea0dXdd9vqai=hGuQ8kuc9pgc9s8qqaq=dirpe0xb9q8qiLsFr0=vr0=vr0dc8meaabaqaciaacaGaaeqabaWaaeGaeaaakeaaimaacqWFZestaaa@3790@| + *k*) + *L *· |O
 MathType@MTEF@5@5@+=feaafiart1ev1aaatCvAUfKttLearuWrP9MDH5MBPbIqV92AaeXatLxBI9gBamrtHrhAL1wy0L2yHvtyaeHbnfgDOvwBHrxAJfwnaebbnrfifHhDYfgasaacH8akY=wiFfYdH8Gipec8Eeeu0xXdbba9frFj0=OqFfea0dXdd9vqai=hGuQ8kuc9pgc9s8qqaq=dirpe0xb9q8qiLsFr0=vr0=vr0dc8meaabaqaciaacaGaaeqabaWaaeGaeaaakeaaimaacqWFoe=taaa@383D@|), except when we use a comparison based sorting in SMOTIF-2 exact matching case, in which case the second term becomes *n *· (|ℳ
 MathType@MTEF@5@5@+=feaafiart1ev1aaatCvAUfKttLearuWrP9MDH5MBPbIqV92AaeXatLxBI9gBamrtHrhAL1wy0L2yHvtyaeHbnfgDOvwBHrxAJfwnaebbnrfifHhDYfgasaacH8akY=wiFfYdH8Gipec8Eeeu0xXdbba9frFj0=OqFfea0dXdd9vqai=hGuQ8kuc9pgc9s8qqaq=dirpe0xb9q8qiLsFr0=vr0=vr0dc8meaabaqaciaacaGaaeqabaWaaeGaeaaakeaaimaacqWFZestaaa@3790@| + *k *+ log *n*).

## Results and discussion

### Performance comparison

We have implemented the SMOTIF algorithm in standard C++. We compare our results with SMARTFINDER, the best previous algorithm for structured motif search. For fair comparison, the suffix tree used in SMOTIF-2 for finding the pos-list of simple motifs is the same as the one used in SMARTFINDER, namely the lazy mode suffix tree [11], and we apply Sellers' dynamic programming algorithm [26] for approximate matching. The programs were compiled with g++ v3.2.2 at the optimization level 3 (-O3). Unless indicated otherwise, we did the experiments on an Apple G5 with dual 2.7Ghz processors and 4GB memory running Mac OS X; the timings reported are total times for all steps of the algorithms; all our experiments use exact matching for the simple motifs, and we report the full position for the occurrences.

#### SMOTIF: *parameter settings*

In the first set of experiments we used the 5 chromosomes of *Arabidopsis thaliana *as our sequence set. These five chromosomes have lengths 29M, 19M, 22.7M, 16.9M and 25.7M base pairs, respectively. The structured motif ℳ
 MathType@MTEF@5@5@+=feaafiart1ev1aaatCvAUfKttLearuWrP9MDH5MBPbIqV92AaeXatLxBI9gBamrtHrhAL1wy0L2yHvtyaeHbnfgDOvwBHrxAJfwnaebbnrfifHhDYfgasaacH8akY=wiFfYdH8Gipec8Eeeu0xXdbba9frFj0=OqFfea0dXdd9vqai=hGuQ8kuc9pgc9s8qqaq=dirpe0xb9q8qiLsFr0=vr0=vr0dc8meaabaqaciaacaGaaeqabaWaaeGaeaaakeaaimaacqWFZestaaa@3790@ to search for includes a well conserved feature of a *Copia *retrotransposon [6,7], shown in Table 7.

**Table 7:** Real Motifs

Copia Motif	TNGA [12,14] TWNYTNNA [19,21] TNTMYRT [4,6] WNCCNNNNRG [72,95] TGNNA [100,125] TNTANRTNRAYGA
Motif ℳ MathType@MTEF@5@5@+=feaafiart1ev1aaatCvAUfKttLearuWrP9MDH5MBPbIqV92AaeXatLxBI9gBamrtHrhAL1wy0L2yHvtyaeHbnfgDOvwBHrxAJfwnaebbnrfifHhDYfgasaacH8akY=wiFfYdH8Gipec8Eeeu0xXdbba9frFj0=OqFfea0dXdd9vqai=hGuQ8kuc9pgc9s8qqaq=dirpe0xb9q8qiLsFr0=vr0=vr0dc8meaabaqaciaacaGaaeqabaWaaeGaeaaakeaaimaacqWFZestaaa@3790@_1_	HNGTNYDNHDNBTNNDNA [0,3] YNHTNYRHGGNBTNAR [0,2] ARDBNBH
Motif ℳ MathType@MTEF@5@5@+=feaafiart1ev1aaatCvAUfKttLearuWrP9MDH5MBPbIqV92AaeXatLxBI9gBamrtHrhAL1wy0L2yHvtyaeHbnfgDOvwBHrxAJfwnaebbnrfifHhDYfgasaacH8akY=wiFfYdH8Gipec8Eeeu0xXdbba9frFj0=OqFfea0dXdd9vqai=hGuQ8kuc9pgc9s8qqaq=dirpe0xb9q8qiLsFr0=vr0=vr0dc8meaabaqaciaacaGaaeqabaWaaeGaeaaakeaaimaacqWFZestaaa@3790@_2_	TNVRNKAYNKNVVNDV [9,11] HNRR [6,8] YDNNVNNV [9,13] HB [4,5] TNNNNRBNYDBDNNRR
Motif ℳ MathType@MTEF@5@5@+=feaafiart1ev1aaatCvAUfKttLearuWrP9MDH5MBPbIqV92AaeXatLxBI9gBamrtHrhAL1wy0L2yHvtyaeHbnfgDOvwBHrxAJfwnaebbnrfifHhDYfgasaacH8akY=wiFfYdH8Gipec8Eeeu0xXdbba9frFj0=OqFfea0dXdd9vqai=hGuQ8kuc9pgc9s8qqaq=dirpe0xb9q8qiLsFr0=vr0=vr0dc8meaabaqaciaacaGaaeqabaWaaeGaeaaakeaaimaacqWFZestaaa@3790@_3_	DNNNNDRYW [2,5] DS [6,7] HMM [1,2] TNDB
Motif ℳ MathType@MTEF@5@5@+=feaafiart1ev1aaatCvAUfKttLearuWrP9MDH5MBPbIqV92AaeXatLxBI9gBamrtHrhAL1wy0L2yHvtyaeHbnfgDOvwBHrxAJfwnaebbnrfifHhDYfgasaacH8akY=wiFfYdH8Gipec8Eeeu0xXdbba9frFj0=OqFfea0dXdd9vqai=hGuQ8kuc9pgc9s8qqaq=dirpe0xb9q8qiLsFr0=vr0=vr0dc8meaabaqaciaacaGaaeqabaWaaeGaeaaakeaaimaacqWFZestaaa@3790@_4_	DBNNNND [48,102] KRRYMYNNNMRNHYNDVNYAYVH [7,10] VNNNYNNND [34,63] WD [2,8] KNNH [3,5] VNDDRNNNNNNHVNNNNNNNHHH

**Figure 11:**
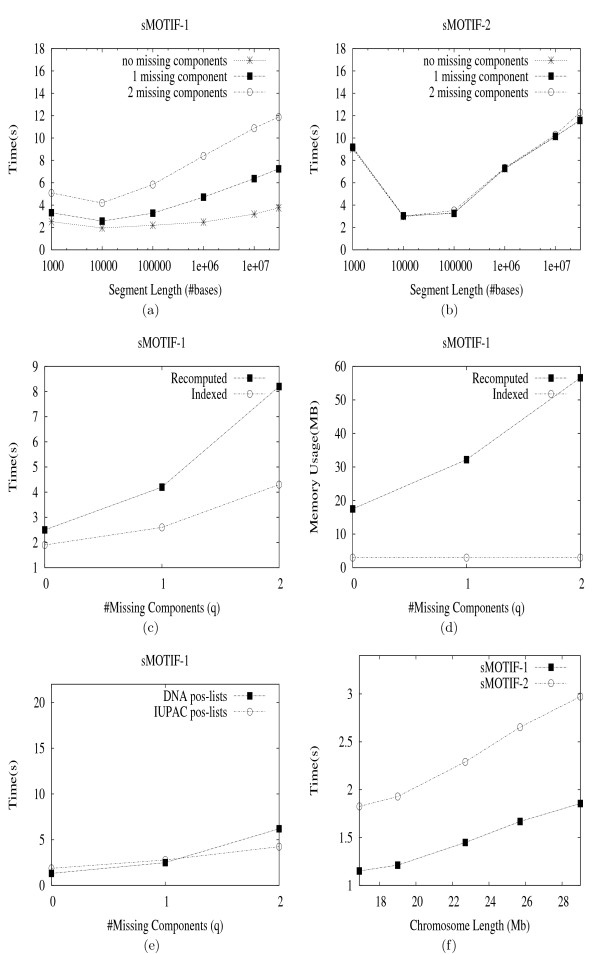
**SMOTIF Performance: Comparing SMOTIF-1 and SMOTIF-2**. The figure shows how SMOTIF performs while searching for the Copia retrotransposon on Chromosome 1 from *A. thaliana*. (a) and (b) show the effect of sequence segmentation on SMOTIF-1 and SMOTIF-2, respectively. (c) and (d) compare the time and memory usage, respectively, for SMOTIF-1, when we use the recomputed or indexed full position recovery (e) compares the DNA versus IUPAC pos-lists for handling IUPAC symbols in SMOTIF-1. (f) shows the time for SMOTIF-1 and SMOTIF-2 on each of the 5 chromosomes of *A. thaliana*, with no missing components.

Figure 11 shows how SMOTIF performs on the *A. thaliana *chromosome 1, while searching for the Copia retrotransposon. We study the impact of allowing 0, 1 or 2 missing components out of the 6 simple motifs in the Copia structured motif. Figure 11(a) and 11(b) show the effect of sequence segmentation on SMOTIF-1 and SMOTIF-2, respectively. The *x*-axis shows the length of the segments, whereas the *y*-axis shows the time. The rightmost end point on the *x*-axis shows the case when the sequence is not segmented.  For these experiments we used the IUPAC pos-list approach. The different curves in each figure show the effect of 0,1, or 2 missing components. Both figures suggest the best segment length is 10,000 base pairs. At that segment length, sequence segmentation can speed up the search around 2 to 3 times for SMOTIF-1 (depending on the number of missing components) and 4 times for SMOTIF-2. In the following experiments, we use 10,000 as the default segmentation length.

Figure 11(c) and 11(d) compare the time and memory usage for SMOTIF-1 when we use the recomputed or indexed full position recovery. We find that the indexed approach is about 2 times faster than recomputing the full positions, and at the same time it can consume up to 20 times less memory! The effects are more pronounced for more missing components. Henceforth, we use the indexed approach to full position recovery.

Figure 11(e) compares the two methods for handling IUPAC symbols in SMOTIF-1, namely DNA pos-lists or IUPAC pos-lists. We find that there is not much difference between the two when 0 or 1 missing components are allowed; but for 2 missing components, IUPAC pos-lists have a slight advantage. In terms of memory space, even though the IUPAC pos-lists approach stores the pos-lists for each distinct symbol that appears in the structured motif, since only one segment is processed at one time, the space utilization of the two approaches is comparable. For example, with no missing components, the IUPAC and DNA pos-lists consume 2.98MB and 2.82MB memory, respectively. Henceforth, we use the IUPAC pos-lists approach.

Figure 11(f) shows the time for SMOTIF-1 and SMOTIF-2 on each of the 5 chromosomes of *A. thaliana*, with no missing components. The chromosomes are arranged by increasing length on the *x*-axis. We find that the search time increases linearly with the lengths of the sequences. We also observe that the direct approach, SMOTIF-1, outperforms the two-step approach, SMOTIF-2, when searching for the Copia retrotransposon.

#### SMOTIF and SMARTFINDER: comparison

##### Comparison on A. thaliana

**Figure 12:**
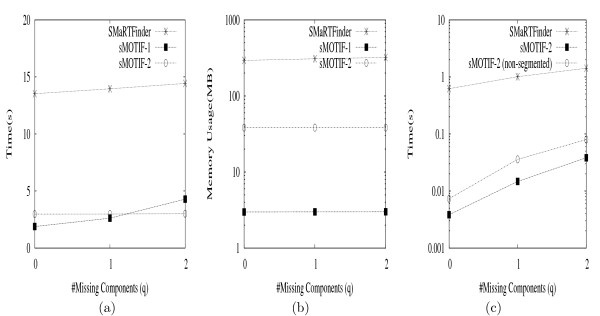
**SMOTIF and SMARTFINDER Comparison: Copia Motif**. The figure compares SMOTIF-1, SMOTIF-2 and SMARTFINDER, when searching for the Copia retrotransposon in chromosome 1 of *A. thaliana*. (a) and (b) compare time and memory usage. (c) compares the constraint graph approach of SMARTFINDER with the positional joins in SMOTIF.

Figure 12(a) and 12(b) compare time and memory usage, respectively, for SMOTIF-1, SMOTIF-2 and SMARTFINDER, when searching for the Copia retrotransposon in chromosome 1 of *A. thaliana*. We can see that SMOTIF-2 is around 5 times faster and takes around 8 times less memory than SMARTFINDER. Their running time and memory usage increase slowly with the number of missing components. This is because in both approaches, the simple motif search step is the same for different number of missing components and usually takes most of the time and memory. SMOTIF-1 always outperforms SMARTFINDER, both in time and memory usage. SMOTIF-1 is more than 7 times faster and takes 100 times less memory than SMARTFINDER! Its time increases linearly with the number of missing components. While SMOTIF-1 is faster when there are no missing components, SMOTIF-2 does better when there are two missing components. This is because SMOTIF-1 does the positional joins on the pos-lists of individual *symbols*, rather than *simple motifs*, so over all the sub-motifs of the structured motif, the pos-lists of simple motifs may be redundantly computed several times in SMOTIF-1, whereas these are computed only once in SMOTIF-2. Thus the time of SMOTIF-1 varies depending on the number of missing components. However, since we keep one pos-list per DNA/IUPAC symbol, the memory usage remains almost unchanged for SMOTIF-1 algorithm.

To directly evaluate SMARTFINDER's constraint graph approach with our positional join approach, in Figure 12(c), we compare the time for the second step of SMOTIF-2 and SMARTFINDER, after the suffix tree is built in the first step. We observe that SMOTIF-2 is orders of magnitude faster than SMARTFINDER in finding all the occurrences that satisfy the structured gap constraints depending on the number of missing components allowed. We conclude that doing positional joins on the inverted index is an efficient way of enumerating all occurrences.

We also multiply aligned 36 *A. thaliana *LTR retrotransposons from Repbase Update [2] database, which belong to *Copia *repeat class, and have *reverse transcriptase *as the keyword. We then extracted four conserved features from the alignment results, shown as ℳ
 MathType@MTEF@5@5@+=feaafiart1ev1aaatCvAUfKttLearuWrP9MDH5MBPbIqV92AaeXatLxBI9gBamrtHrhAL1wy0L2yHvtyaeHbnfgDOvwBHrxAJfwnaebbnrfifHhDYfgasaacH8akY=wiFfYdH8Gipec8Eeeu0xXdbba9frFj0=OqFfea0dXdd9vqai=hGuQ8kuc9pgc9s8qqaq=dirpe0xb9q8qiLsFr0=vr0=vr0dc8meaabaqaciaacaGaaeqabaWaaeGaeaaakeaaimaacqWFZestaaa@3790@_*i *_(*i *∈ [1, 4]) in Table 7. We searched chromosome 1 of *A. thaliana *for the four extracted motifs using SMARTFINDER, SMOTIF-1 and SMOTIF-2, respectively. Table 8 shows the number of occurrences |O
 MathType@MTEF@5@5@+=feaafiart1ev1aaatCvAUfKttLearuWrP9MDH5MBPbIqV92AaeXatLxBI9gBamrtHrhAL1wy0L2yHvtyaeHbnfgDOvwBHrxAJfwnaebbnrfifHhDYfgasaacH8akY=wiFfYdH8Gipec8Eeeu0xXdbba9frFj0=OqFfea0dXdd9vqai=hGuQ8kuc9pgc9s8qqaq=dirpe0xb9q8qiLsFr0=vr0=vr0dc8meaabaqaciaacaGaaeqabaWaaeGaeaaakeaaimaacqWFoe=taaa@383D@| found, and the search times. We can observe that since we allow no missing components, SMOTIF-1 can be 4 to 5 times faster than SMOTIF-2, and 4 to 18 times faster than SMARTFINDER. Note also that for the longest motif ℳ
 MathType@MTEF@5@5@+=feaafiart1ev1aaatCvAUfKttLearuWrP9MDH5MBPbIqV92AaeXatLxBI9gBamrtHrhAL1wy0L2yHvtyaeHbnfgDOvwBHrxAJfwnaebbnrfifHhDYfgasaacH8akY=wiFfYdH8Gipec8Eeeu0xXdbba9frFj0=OqFfea0dXdd9vqai=hGuQ8kuc9pgc9s8qqaq=dirpe0xb9q8qiLsFr0=vr0=vr0dc8meaabaqaciaacaGaaeqabaWaaeGaeaaakeaaimaacqWFZestaaa@3790@_4_, SMARTFINDER ran out of memory.

**Table 8:** Search Time for Several Real Motifs

ℳ MathType@MTEF@5@5@+=feaafiart1ev1aaatCvAUfKttLearuWrP9MDH5MBPbIqV92AaeXatLxBI9gBamrtHrhAL1wy0L2yHvtyaeHbnfgDOvwBHrxAJfwnaebbnrfifHhDYfgasaacH8akY=wiFfYdH8Gipec8Eeeu0xXdbba9frFj0=OqFfea0dXdd9vqai=hGuQ8kuc9pgc9s8qqaq=dirpe0xb9q8qiLsFr0=vr0=vr0dc8meaabaqaciaacaGaaeqabaWaaeGaeaaakeaaimaacqWFZestaaa@3790@	|O MathType@MTEF@5@5@+=feaafiart1ev1aaatCvAUfKttLearuWrP9MDH5MBPbIqV92AaeXatLxBI9gBamrtHrhAL1wy0L2yHvtyaeHbnfgDOvwBHrxAJfwnaebbnrfifHhDYfgasaacH8akY=wiFfYdH8Gipec8Eeeu0xXdbba9frFj0=OqFfea0dXdd9vqai=hGuQ8kuc9pgc9s8qqaq=dirpe0xb9q8qiLsFr0=vr0=vr0dc8meaabaqaciaacaGaaeqabaWaaeGaeaaakeaaimaacqWFoe=taaa@383D@|	SMARTFINDER	SMOTIF-1	SMOTIF-2
1	27	17.44s	3.98s	3.80s
2	446	85.68s	4.98s	19.73s
3	507911	84.11s	4.69s	21.98s
4	283676	Out of memory	10.61s	50.86s

##### Searching motifs with long gaps

One application of SMOTIF is to find structured motifs with long gaps. For example, we extracted the motif DNNNNDRYW [2578, 4202]RNNGVHVY, from the same 36 LTRs of *A. thaliana *mentioned above. Here we retain only the first and last conserved components in the resulting alignment of the 36 sequences. Note the relatively long gap ranges, with minimum gap *l *= 2578 and maximum gap *u *= 4202. SMOTIF was able to extract the approximately 83 million full positions, corresponding to 3 million start positions, in just 35.75s (SMOTIF-1) and 15.72s (SMOTIF-2).

##### Comparison on random motifs

Here we used chromosome 20 of *Homo Sapiens *as the sequence; it has length 61M base pairs. We generate 100 random structured motifs in the Σ_IUPAC _alphabet, with *k *∈ [3,8] simple motifs of length *l *∈ [5,10] (*k *and *l *are selected uniformly at random within the given ranges). The gap range between each pair of simple motifs is a random sub-interval of [-5, 100]. Note that the negative minimum gap shows that SMOTIF can mine overlapping simple motifs. Here we also compare the structured profile search approach SMOTIF-P, as follows: for each random motif, we form a profile by first expanding the IUPAC symbols into their corresponding DNA symbols and assign them a random probability of occurrence which accounts for 90% of the share, whereas the other DNA symbols randomly share in the remaining 10%. We use SMOTIF-P to search for the profiles with 2 as the number of core positions in each simple motif, λ^*c *^= 0.1 as the core score threshold and λ = 0.6 as the total score threshold. We use random motifs mainly to demonstrate the effects of various parameters on SMOTIF and SMARTFINDER.

**Figure 13:**
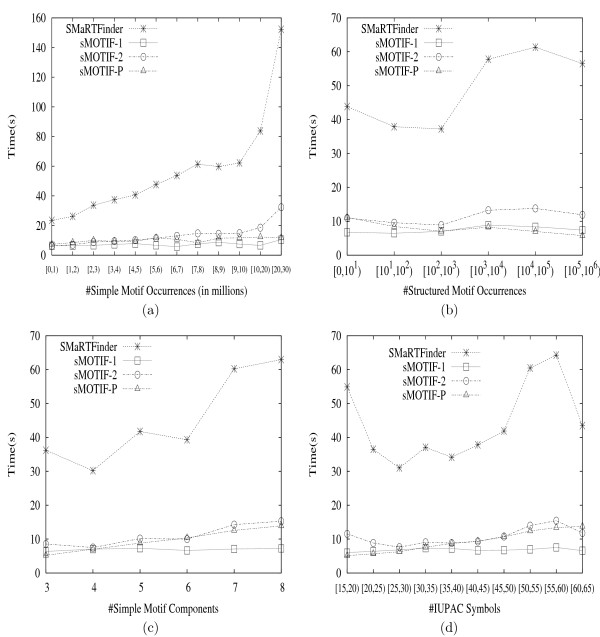
**SMOTIF and SMARTFINDER Comparison: Random Motifs**. The figure compares SMOTIF-1, SMOTIF-2 and SMARTFINDER, when searching for 100 randomly generated structured motifs in chromosome 20 of *Homo sapiens*. (a) shows how the running time varies with the sum of the number of occurrences of the simple motifs in each of the 100 random motifs. (b) shows the time with respect to the number of occurrences of the whole structured motif. (c) shows the effect of the number of simple motif components in the structured motif. (d) shows the impact of the number of IUPAC symbols in the structured motifs.

Figure 13(a)–(d) show the results. Here we do not allow missing components. As noted before, we may find overlapping occurrences if a negative gap is present in a motif. Figure 13(a) shows how the running time varies with the sum of the number of occurrences of the simple motifs in each of the 100 random motifs. For clarity, each point reflects the average time for the number of occurrences in the given range on the x-axis. For example, the first point on the x-axis [0, 1) corresponds to the case when there are between 0 and 1 million occurrences found. The general trend is that it takes more time as the number of occurrences increases. Figure 13(b) shows the time with respect to the number of occurrences of the whole structured motif (again, for clarity, only average times are plotted for occurrences in the given ranges in the x-axis). We observe that the time increases slightly with increase in the occurrences. In general, the times are more sensitive to the number of intermediate (simple) occurrences. Figure 13(c) shows the effect of the number of simple components in the structured motif. Each point shows the average time over all motifs having the given number of simple motifs. Here again the time increases with increasing components. Finally Figure 13(d) shows the impact of the number of IUPAC symbols in the structured motifs; the trend being that the more the symbols the more time it takes to search. We also observe that the approaches scale linearly, on average, with respect to the different parameters. Also SMOTIF remains about 5–10 times faster than SMARTFINDER over all these experiments.

Table 9 shows the mean and variance of the search times over all the 100 structured motifs. It also shows the time for finding only the start positions or the full positions for SMOTIF. Overall, for these random motifs, we find that on average SMOTIF-1 is the fastest, SMOTIF-2 and SMOTIF-P are comparable, and all three outperform SMARTFINDER by a factor of 4 to 6.

**Table 9:** Random Motifs: Mean and Variance

Algorithm	Mean(s)	Variance (s)
SMARTFINDER	44.42	24.85
SMOTIF-1 (full)	6.97	1.45
SMOTIF-1 (start)	6.93	1.46
SMOTIF-2 (full)	10.83	5.07
SMOTIF-2 (start)	10.81	5.07
SMOTIF-P (full)	9.67	2.95
SMOTIF-P (start)	9.66	2.97

full gives the time for full position recovery, whereas start gives the time for reporting only the start positions.

It is interesting to note that SMOTIF is more stable than SMARTFINDER: SMOTIF-P has around 8 times less variance than SMARTFINDER, SMOTIF-2 has around 5 times less variance than SMARTFINDER, whereas SMOTIF-1 has around 17 times less variance than SMARTFINDER. Note also that the overhead in recovering the full positions from the start positions is negligible.

### Application: composite regulatory patterns

The complex transcriptional regulatory network in Eukaryotic organisms usually requires interactions of multiple transcription factors. A potential application of SMOTIF is to search for such composite regulatory binding sites in DNA sequences. We took two such transcription factors, URS1H and UASH, that are known to cooperatively regulate 11 yeast genes [28]. These 11 genes are also listed in SCPD [1], the promoter database of *Saccharomyces cerevisiae*. In 10 of those genes the URS1H binding site appears downstream from UASH; in the remaining one (HOP1) the binding sites are reversed. We took the binding sites for the 10 genes, and after their multiple alignment, we obtained the composite motif NNDTBNGDWGDNNDH[5,179]WBRGCSGCYVW, where we represent each column in the alignment with the IUPAC symbol corresponding to the bases that appear at that position. We also extracted the profile for these 10 binding sites. Table 10 shows the binding sites for the 10 genes, their alignment, and the start positions and the distances between the sites (the difference of start positions). The smallest distance is 20 and the largest is 194. Since these are start positions, the variable gap range is obtained by subtracting the length (15) of UASH to obtain *l *= 20 – 15 = 5 and *u *= 194 – 15 = 179. Notice also how the alignment preserves the highly conserved GCSGC region in URS1H.

**Table 10:** UASH and URS1H Binding Sites

**Genes**	**UASH**		**URS1H**		**Distance**
		
	Site	Pos	Site	Pos	
ZIP1	GATTCGGAAGTAAAA	-42	==TCGGCGGCTAAAT	-22	20
MEI4	TCTTTCGGAGTCATA	-121	==TGGGCGGCTAAAT	-98	23
DMC1	TTGTGTGGAGAGATA	-175	AAATAGCCGCCCA==	-143	32
SPO13	TAATTAGGAGTATAT	-119	AAATAGCCGCCGA==	-100	19
MER1	GGTTTTGTAGTTCTA	-152	TTTTAGCCGCCGA==	-115	37
SPO16	CATTGTGATGTATTT	-201	==TGGGCGGCTAAAA	-90	111
REC104	CAATTTGGAGTAGGC	-182	==TTGGCGGCTATTT	-93	89
RED1	ATTTCTGGAGATATC	-355	==TCAGCGGCTAAAT	-167	188
REC114	GATTTTGTAGGAATA	-288	==TGGGCGGCTAACT	-94	194
MEK1	TCATTTGTAGTTTAT	-233	==ATGGCGGCTAAAT	-150	83

Motif	NNDTBNGDWGDNNDH		==WBRGCSGCYVW==		[5,179]

We then searched for the structured motif in the upstream regions of all 5873 genes in the yeast genome. We used the -800 to -1 upstream regions, and truncated the segment if it overlaps with an upstream open reading frame (ORF). As a result, 5794 sequences with average length of 497 bases are left. By searching for the IUPAC pattern, we found 65 occurrences, including the 10 originally known sites, within 1 second. By searching for the profile with 5 as the number of core positions in each simple motif, λ_*c *_= 0.6 as the core score threshold and λ = 0.8 as the total score threshold, we found 56 occurrences in 0.18 seconds. For each occurrence, we then extracted its actual sequence segment in the matching upstream regions. Since the structured motif represented by IUPAC symbols may be too general, for each matching segment, we calculated its hamming distance to one of the 10 known binding sites. We then selected an occurrence as a possibly new binding site if the minimum hamming distance to any of the 10 known sites is within a given maximum threshold value. Table 11 lists the 12 newly found occurrences in the entire yeast genome (upstream regions) using a hamming distance threshold of 5. The sites discovered using both pattern and profile search are listed. These new occurrences could be putative binding sites for the two transcription factors UASH and URS1H.

**Table 11:** Potential Binding Sites

**Genes**	**UASH**		**URS1H**		**Hamming Distance**
		
	Site	Pos	Site	Pos	
MES1	GATTTTGAAGTAGGA	-438	TTAGCCGCCGA	-246	5:MER1
YJL045W	TTTTGTGAAGAGATA	-407	TTAGCCGCTCA	-273	4:DMC1
HSP60	GTTTTTGTAGGTATA	-329	ATAGCCGCCCA	-252	5:MER1
SPO1	ATTTTTGAAGTTAAC	-192	TCAGCGGCTAT	-90	5:RED1
MEK1	TCATTTGTAGTTTAT	-233	TCGGCGGCTAT	-136	3:MEK1
YIG1	ATTTCCGGAGTTTTC	-183	TCGGCGGCTAT	-140	5:RED1

^†^AGP1	CCTTTTGATGACTTT	-786	TCGGCGGCTAA	-699	5:SPO16
^†^AGPl	CCTTTTGATGACTTT	-786	TCGGCGGCTAA	-668	5:SPO16
^†^REC114	CATTTTGGTGGGTTC	-158	TGGGCGGCTAA	-94	5:SPO16
^†^GNTl	TCATTTGGAGAATAT	-340	ATAGCCGCCAT	-299	5:SPO13

^‡^MEK1	TTATATGCAGTATAT	-276	ATGGCGGCTAA	-150	4:MEK1
^‡^MMS1	AACTCTGTAGTTATA	-643	TGGGCGGCTAA	-497	5:REC114

For each occurrence we give the gene names corresponding to the upstream region, the sites and positions for UASH and URS1H, and also the hamming distance and the closest known gene with the cooperative binding sites. For example, 5:SPO16 in the first row means that the hamming distance between AGP1 and SPO16 was 5. ^†^: found only by IUPAC pattern search, ^‡^: found only by profile search.

Upon further analysis, we found that in fact, the new occurrence in MEK1 (at positions -233,-136) that we found is also listed in the SCPD database as a binding site. SCPD lists one site for UASH at position -233, and two sites for URS1H at positions -136 and -150. To construct the motif, we had used -150 as the site for URS1H, without knowledge of the other site. SMOTIF was thus able to automatically find the other site based on the extracted motif! For REC114, we also found another occurrence at positions -158 (UASH) and -94 (URS1H). However, this is not reported in SCPD.

To further analyze the remaining new occurrences, we consulted the SGD (*Saccharomyces *Genome Database) Gene Ontology Term Finder [29] to find the inter-relationships between the genes. The first three rows of Table 12 show the significant GO terms (biological process or molecular function) that are common to the genes corresponding to a new occurrence and its closest (known) gene. The rest of the table shows the significant terms among the 18 genes. These results indicate that at least some of the new occurrences (such as SPO1, HSP60, MES1, and GNT1) have a potential to be binding sites since they share some significant processes with the known sites' genes. Out of these SPO1 has the highest potential to be a new binding site, since it is known that UASH and URS1H are involved in early meiotic expression, during sporulation [28]. Table 12 shows that SPO1 shares *meiosis *and *M phase of meiotic cell cycle *with the rest of the genes. After searching for SPO1 in SGD database, we found that SPO1 is a transcriptional regulator involved in sporulation, and required for middle and late meiotic expression. This increases our confidence that SPO1 has high potential to be a previously unknown binding site.

**Table 12:** Genes and Significant Gene Ontology (GO) Terms

**Genes**	**Significant GO Terms**	**p-value**
**MES1**, MER1	RNA metabolism	5.7*e*^-3^
**HSP60**, MER1	nucleic acid binding	7.9*e*^-3^
**SPO1**, RED1	M phase-meiotic cell cycle, meiotic cell cycle, meiosis, M phase	1.1*e*^-3^
^‡ ^**MMS1**, REC114	DNA recombination, DNA metabolism	6.0*e*^-3^
**MES1**, REC114, ^† ^**GNT1**, MEK1, MEI4, DMC1, MER1, REC104	biopolymer metabolism, macromolecule metabolism	2.3*e*^-4^
REC114, **SPO1**, MEK1, ZIP1, MEI4, DMC1, SPO13, MER1, REC104, RED1, HOP1	meiosis, M phase of meiotic cell cycle, meiotic cell cycle, M phase, cell cycle	1.6*e*^-14^
**HSP60**, DMC1, RED1, HOP1	DNA binding	6.7*e*^-7^
**HSP60**, DMC1, RED1	structure-specific DNA binding	3.1*e*^-8^
**HSP60**, DMC1	single-stranded DNA binding	3.7*e*^-6^
MEI4, DMC1, REC104, REC114, ^‡ ^**MMS1**	DNA recombination, DNA metabolism	2.8*e*^-6^
MEI4, DMC1, MER1, REC104, REC114, MEK1, **MES1**, ^‡ ^**MMS1**	biopolymer metabolism, macromolecule metabolism	2.3*e*^-4^

^† ^: found only by IUPAC pattern. search, ^‡ ^: found only by profile search.

Finally, since we knew that in gene HOP1, the URS1H binding site appears upstream from UASH, we wanted to see if we could extract the "reversed" binding site. We search for the original and the reversed motifs using a hamming threshold of 6. We found 34 new binding sites where UASH can appear either up-or down-stream from URS1H. Among these we found two possible potential binding sites for the gene HOP1, with UASH at position -201 and URS1H at positions -534 and -175. The former pair (-201,-534) is in fact a known binding site as reported in the SCPD database [1]. This once again showcases the ability of SMOTIF to find potential new binding sites.

## Conclusion

We introduced SMOTIF, a fast and efficient algorithm to search structured pattern and profile motifs in biological sequences. We showed its applications in searching for composite regulatory patterns, long terminal repeat retrotransposons, and for searching long range motifs. SMOTIF is also computationally more efficient than previous state-of-the-art methods like SMARTFINDER [6].

In biosequence analysis there are four related structured motif problems depending on whether the simple motifs and gap ranges in the structured motif are known or not: (i) *Structured motif search *[4-6]: all simple motifs and gap ranges are known; (ii) *Structured motif extraction *[30-32]: all simple motifs are unknown and all gap ranges are known; (iii) *Extended structured motif search*: all simple motifs are known and all gap ranges are unknown; and (iv) *Extended structured motif extraction*: all simple motifs and gap ranges are unknown. In this paper we tackled problem (i). A companion paper [33] tackles problem (ii). In the future, we plan to develop efficient algorithms for the other two motif problems as well.

## Authors' contributions

All authors contributed equally to this work.
